# The Synthesis of 2′-Hydroxychalcones under Ball Mill Conditions and Their Biological Activities

**DOI:** 10.3390/molecules29081819

**Published:** 2024-04-17

**Authors:** Imen Abid, Wassim Moslah, Sandrine Cojean, Nicolas Imbert, Philippe M. Loiseau, Alain Chamayou, Najet Srairi-Abid, Rachel Calvet, Michel Baltas

**Affiliations:** 1 LCC (Laboratoire de Chimie de Coordination), UPR CNRS 8241, Université de Toulouse, UPS, INPT, Inserm ERL 1289, 205 Route de Narbonne, BP 44099, CEDEX 4, F-31077 Toulouse, France; imen.abid@lcc-toulouse.fr; 2 Centre RAPSODEE (Recherche d’Albi en génie des Procédés des SOlides Divisés, de l’Energie et de l’Environnement), IMT Mines Albi, UMR CNRS 5302, Université de Toulouse, Campus Jarlard, Allée des Sciences, CEDEX 09, F-81013 Albi, France; alain.chamayou@mines-albi.fr; 3 Laboratoire des Biomolecules, Venins et Applications Théranostiques (LR20IPT01), Institut Pasteur de Tunis, Université Tunis-ElManar, Tunis 1002, Tunisia; wassim.moslah@pasteur.utm.tn (W.M.); najet.abidsrairi@pasteur.tn (N.S.-A.); 4 BioCIS (Biomolécules: Conception, Isolement et Synthèse), UMR CNRS 8076, Université Paris-Saclay, F-91400 Orsay, France; sandrine.cojean@universite-paris-saclay.fr (S.C.); nicolas.imbert@universite-paris-saclay.fr (N.I.); philippe.loiseau@universite-paris-saclay.fr (P.M.L.); 5 National Malaria Reference Centre, AP-HP, Hôpital Bichat—Claude Bernard, 46 Rue Henri Huchard, F-75018 Paris, France

**Keywords:** ball mill mechanochemistry, sustainable synthesis, chalcones, phenolics, antiparasitic, antitumoral

## Abstract

Chalcones are polyphenols that belong to the flavonoids family, known for their broad pharmacological properties. They have thus attracted the attention of chemists for their obtention and potential activities. In our study, a library of compounds from 2′-hydroxychalcone’s family was first synthesized. A one-step mechanochemical synthesis via Claisen–Schmidt condensation reaction under ball mill conditions was studied, first in a model reaction between a 5′-fluoro-2′-hydroxyacetophenone and 3,4-dimethoxybenzaldehyde. The reaction was optimized in terms of catalysts, ratio of reagents, reaction time, and influence of additives. Among all assays, we retained the best one, which gave the highest yield of 96% when operating in the presence of 1 + 1 eq. of substituted benzaldehyde and 2 eq. of KOH under two grinding cycles of 30 min. Thus, this protocol was adopted for the synthesis of the selected library of 2′-hydroxychalcones derivatives. The biological activities of 17 compounds were then assessed against *Plasmodium falciparum*, *Leishmania donovani* parasite development, as well as *IGR-39* melanoma cell lines by inhibiting their viability and proliferation. Compounds **6** and **11** are the most potent against *L. donovani,* exhibiting IC_50_ values of 2.33 µM and 2.82 µM, respectively, better than the reference drug Miltefosine (3.66 µM). Compound **15** presented the most interesting antimalarial activity against the 3D7 strain, with IC_50_ = 3.21 µM. Finally, chalcone **12** gave the best result against IGR-39 melanoma cell lines, with an IC_50_ value of 12 µM better than the reference drug Dacarbazine (IC_50_ = 25 µM).

## 1. Introduction

**Figure 1:**
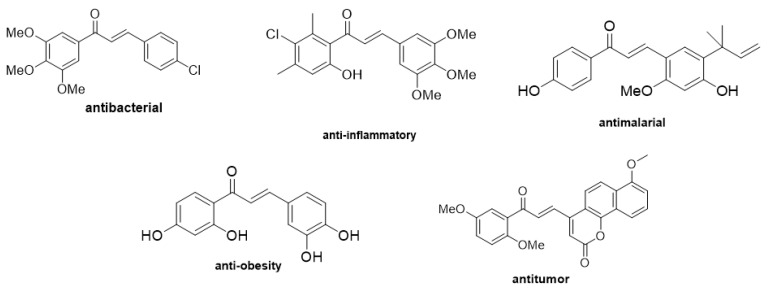
Examples of chalcones with pharmacological properties.

Chalcones are plant-based polyphenols of the flavonoids family, along with flavones, flavonols, isoflavonoids, flavanols, flavanones, and anthocyanins [1]. The chalcone synthase is the enzyme responsible for their biosynthesis and is considered the first step in the production of flavonoids [2]. In the medicinal chemistry domain, chalcones are effective templates for drug development. Several biological and pharmacological properties are attributed to natural or synthetic chalcones, such as antibacterial [3], antifungal [4], antimalarial [5], anti-inflammatory [6], anti-obesity [7], and antitumor [8] (Figure 1). Chalcones are also reported for inhibiting oxidative radical formation in different in vitro and in vivo models [9] and enhancing antioxidant defense in animals [10]. Chalcones have also been reported to reduce cytokine production and nitric oxide synthase activity in macrophages [6,11] and to regulate prostaglandin synthesis and the NF-kB pathway [12].

**Scheme 1:**
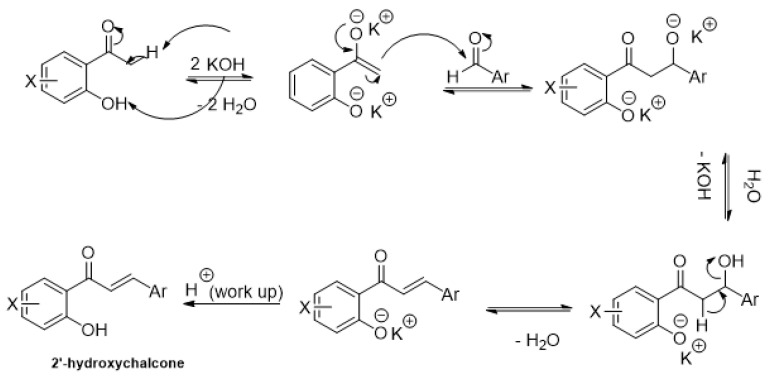
Mechanism of Claisen–Schmidt reaction.

While studying chalcones in plants is an important issue for deciphering their biological activities and their mode of action, in the medicinal chemistry domain, their extraction from plants presents some disadvantages. In fact, they are not well represented in plants, and their purification could be difficult, affording generally low final yields. In that respect, synthetic approaches for their obtention have gained considerably much importance, particularly elaborating bio-inspired families of chalcones for QSAR studies [13,14,15]. Chemically, the chalcone’s structure consists of two aromatic rings joined with an *α*,*β*-unsaturated bond and a carbonyl group. Many techniques and processes for their synthesis have been reported, such as Heck coupling, Suzuki–Miyaura coupling, Friedel–Crafts acylation, Wittig reaction, and Claisen–Schmidt condensation [16,17]. The latter is the easiest and most common method, with a proposed mechanism consisting of four steps (Scheme 1).

Despite that, this reaction usually has some disadvantages, typically requiring a long reaction time, long work-up, and sometimes reflux conditions. The conversion may vary from 10% to 100%, depending on different parameters such as reactants and catalysts. The reaction in a solution can often generate complex mixtures.

One of the main goals in the area of organic synthesis oriented toward biologically active compounds is the research and development of efficient and environmentally safe methods in terms of efficiency, waste management, and energy input, issues now addressed and termed «Green Chemistry» [18]. Alternative energy sources that appeared and developed in the last two decades are photochemistry via light excitation, microwave, sonochemistry irradiation, and mechanochemistry [19]. According to IUPAC, a mechanochemical reaction is a “Chemical reaction that is induced by the direct absorption of mechanical energy”. Mechanochemistry for organic compounds started to be developed after the pioneering work reported by Toda in the 1980s [20] and Kaupp [21]. Nowadays, mechanochemical synthesis has emerged as an efficient approach applicable in different fields [22,23,24,25], such as catalysis, polymers, nanomaterials, and organic synthesis used for creating carbon–carbon, carbon–heteroatom, and metal–ligand coordination bonds. In the last decade, mechanochemical synthesis, as an eco-friendly approach, was studied and assessed by using green metrics in comparison to conventional syntheses, for example, in the synthesis of 1,2-4-annulated triazoles [26] or Active Pharmaceutical Ingredients (API) for the pharmaceutical industry [27].

In terms of the experimental method, traditional grinding by using a mortar and a pestle has been replaced by more sophisticated ball milling or mechano-milling techniques that are generally conducted in vibration or planetary mills. Kudličková et al. [28] adopted this methodology to apply it in the Claisen–Schmidt reaction in order to create a library of chalcones with antiproliferative activity by condensing 1-methylindole-3-carboxaldehyde with 4′-bromoacetophenone [29].

They investigated the effect of the equivalents of catalysts, milling times, type of milling materials, and mainly, the effect of solvent addition (liquid-assisted grinding, LAG) on the issue of the reaction.

Several years ago, we launched a research program focused on the mechanochemical synthesis of various families of small organic molecules with potential biological activities. Among them were hydrazones and 1,2,4-triazoles [26,29].

In continuation of this research work, we focused our attention on 2′-hydroxychalcones, which are also organic compounds well known for their health benefits. Herein, we report our first findings in the construction of a library of this latter by coupling a variety of 2′-hydroxyacetophenones with benzaldehydes under ball mill conditions. Thus, 17 compounds were synthesized and evaluated for their antiparasitic properties against *Plasmodium falciparum*, *Leishmania donovani*, and for their cytotoxic properties, as well as for their antitumoral effects against human melanoma-derived cell line IGR-39.

## 2. Results and Discussion

### 2.1. Research of Optimal Conditions in a Model Reaction

**Figure 2:**
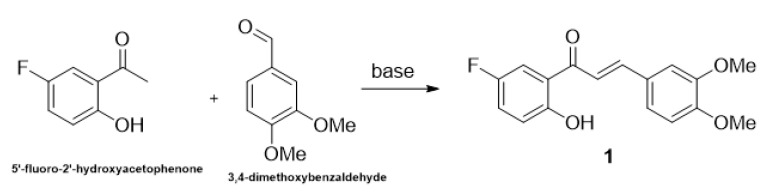
Model Claisen–Schmidt reaction studied.

In order to establish a protocol that can be adopted for the synthesis of our series of 2′-hydroxychalcones, we first studied the operating conditions when reacting 5′-fluoro-2′-hydroxyacetophenone with 3,4-dimethoxybenzaldehyde. The reaction was conducted in a vibratory ball mill MM400, with a working frequency of 30 Hz, equipped with two zirconium dioxide 10 mL jars (internal Ø 20 mm); each jar was equipped with two balls of 10 mm Ø. The choice of the reagents was based on the values of melting points between various benzaldehydes and 2′-hydroxyacetophenones, and we chose for the model study the 5′-fluoro-2′-hydroxyacetophenone (mp 56–58 °C) and 3,4-dimethoxybenzaldehyde (mp 40–43 °C). In addition, the fluorine atom can be used as another NMR spectroscopic indicator for the issue of the reaction. Thus, for the reaction model (Figure 2), the results obtained are shown in Table 1.

All reactions were studied for 1.2 mmol of 5′-fluoro-2′-hydroxyacetophenone. For all reactions, we maintained the same work-up: the obtained powders after grinding were powered in cold MeOH (5–10 mL) acidified with 2 mL of a cold HCl (1 M) and then filtrated and washed with water before drying under a vacuum. The powders obtained were weighted and underwent all spectroscopic analyses, showing the sole chalcones. The filtrate was also analyzed after ethyl acetate extraction, showing only residual starting materials.

Three different bases were used, namely NaOH, LiOH, and KOH. When operating in the presence of NaOH and in a 1:1:1 ratio, no reaction occurred after two cycles of 15 min grinding (Table 1, entry 1). The same reaction afforded low yields of chalcone (yields 20 and 23%) when operating in the presence of 2 or 3 eq., respectively (Table 1, entries 2 and 3). Under the same conditions (3 eq. of base and 2 × 15 min grinding), no reaction occurred when operating with LiOH (Table 1, entry 4).

Potassium hydroxide as a base gave much better results. When operating in the presence of 2 or 3 eq. of KOH (Table 1, entries 5 and 6) and after grinding of 2 × 15 min, the desired chalcone **3** was obtained in 40 and 43% yield, respectively. The yield of the reaction increased to 78% when operating for a 2 × 30 min grinding time under a 1:1:2 ratio (Table 1, entry 7) chosen for atom economy as the yield was not significantly increased between 2 and 3 eq. of the base. Most gratifyingly, the yield of chalcone obtained was excellent (96%) when operating in the equimolar medium in the presence of 2 eq. KOH for the first 30 min cycle and adding another eq. of aldehyde for the second cycle (Table 1, entry 8). These conditions were the best obtained when no additive was used.

We also looked at the reaction when an additive is present. Two basic or neutral additives (K_2_CO_3_ and alumina) were tested. Potassium carbonate can be considered a mild dehydrating agent so it can adsorb water issued from the reaction. Alumina was reported by Kakati et al. [30] as an additive in the obtention of chalcones under microwave-assisted solvent-free conditions. When K_2_CO_3_ (1 or 3 eq.) was used in the presence of KOH, the same trends were observed (Table 1, entries 9, 10, and 11), no matter what the equivalents of K_2_CO_3_ were. The best result (95% yield) was obtained when operating under a 1:2:2 ratio of reactants and after 1 h of grinding. Finally, alumina was used as an additive. No reaction was obtained when using neutral or basic alumina (1 or 3 eq.) and operating in the presence of KOH, in a 1:1:2 ratio and after 2 × 15 min of grinding (Table 1, entry 12).

Finally, according to all these optimization tests of the Claisen–Schmidt reaction for the formation of chalcone, we retained the best protocol, which gave the best yield without using an additive when operating in the presence of 1 + 1 eq. of substituted benzaldehyde and 2 eq. of KOH (Table 1, entry 8), and this latter was used for the synthesis of all 2′-hydroxychalcone derivatives.

The molecular structure of this compound was first confirmed by spectral methods. The ^1^H and ^13^C chemical shifts of compound **1** fluorine decoupled in CDCl_3_ are given in Table 2, along with its structure indicating the evidenced correlations. The decoupled experiments were conducted on a 400 MHz Bruker Avance II probe TXO ^13^C observe decoupling ^1^H; ^19^F (see Supporting Information S1 and S2). The attribution of chemical shifts for ^1^H and ^13^C was made with NMR 2D cosy and NMR 2D HSQC (see Supporting Information S3 and S4 respectively).

The most important signals identifying the chalcone are those of the formation of the ethylenic system, which appears as a doublet at 7.42 ppm and 7.91 ppm, resulting from the condensation between the 2′-hydroxyacetophenone and the substituted benzaldehyde and showing a coupling constant *J* = 15.6 Hz, which corresponds to the conformation *E* (trans), the stable conformation of the *α*,*β*-unsaturated ketone. The scaffold of this molecule was identified with the ^13^C chemical shifts of the carbonyl function at 193.2 and the ethylenic function at 117.16 ppm and 146.6 ppm. The ^19^F NMR spectra showed the characteristic signal of fluorine of compound **1** at 124.26 ppm (see Supporting Information S72).

The analysis of Hight Resolution mass spectrometry shows the molecular signal [MH]^+^ = 303.1033, accompanied by adduct [MC_2_H_5_]^+^ = 332.1405 and [MC_3_H_5_]^+^ = 343.1410 (see Supporting Information S7).

### 2.2. Reactions of 5′-Halogenated-2′-Hydroxychalcones and Methoxylated Benzaldehydes

**Figure 3:**
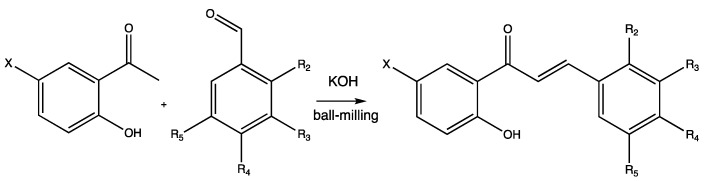
Reaction of chalcones synthesis under ball milling conditions.

Based on the best reaction conditions found (Table 1, entry 8) we evaluated the synthesis of a small library by varying the halogen in the 5′-position of the 2′-hydroxyacetophenone and various methoxylated benzaldehydes (Figure 3). For all reactions, we operated in the presence of 2 eq. of KOH, 1 eq. of ketone and 1 + 1 eq. of substituted benzaldehyde, the second equivalent added after the first 30 min of grinding. The total time of the reaction was 60 min. The results are presented in Table 3.

For all acetophenones, five aldehydes were tested, possessing all methoxy substituents at different positions of ring B.

Concerning reactions with the 5′-fluoro-2′-hydroxyacetophenone, the three different disubstituted benzaldehydes afforded high to excellent yields. The 3,4 and 3,5-dimethoxybenzaldehydes led to the corresponding chalcones **1** and **5** in 96% and 88% yield, respectively while the 2,3-dimethoxybenzaldehyde afforded chalcone **2** in 74% yield (Table 3, entries 1, 5 and 2, respectively). Among the two trimethoxy substituted benzaldehydes, the 3,4,5-trimethoxy and the 2,4,5-trimethoxybenzaldehyde afforded both a very good yield of 72% and 84%, respectively (Table 3, entries 3 and 4). All compounds possess the characteristic unique signal of fluorine at the ^19^F NMR (in CDCl_3_) spectra at 124.26 ppm. Classical synthesis of compound **4** was reported by Kamble et al. in 2011 [31]. The compound was obtained in 80% yield after 6–8 h of reaction in ethanol/KOH solution then acidic work-up followed by silica gel column chromatography. No melting point nor fluorine spectra is presented. 

Concerning reactions with 5′-chloro-2′-hydroxyacetophenone, we obtained excellent yields for all disubstituted methoxy benzaldehydes, i.e., 92%, 95%, and 94% for chalcones **6**, **7**, and **9**, respectively (Table 3, entries 6, 7, and 10). For the trisubstituted benzaldehydes, while the 3,4,5-trimethoxybenzaldehyde led to the corresponding chalcone **8** in 88% yield, surprisingly, no reaction was observed for the 2,4,5-trimethoxybenzaldehyde under the optimal conditions (Table 3, entry 9). No reaction was obtained either under the other experimental conditions reported in Table 1. Concerning this subfamily of chalcones, Albogami et al. reported in 2012 [32] the synthesis of compound **8** in 86% yield. The reaction was carried in methanol in the presence of a catalytic amount of aq. KOH by using a microwave irradiations for 2 min then and by precipitating in an acidic solution overnight. On the other hand, Detsi et al. reported in 2021 [33] a synthesis of compounds **6** and **7** obtained after a 24 h reaction in ethanol/aq. KOH solution and at room temperature (or 4 h under reflux) with yields of 72% and 50%, respectively.

Concerning reactions with 5′-bromo-2′-hydroxyacetophenone, we obtained good yields for all disubstituted dimethoxybenzaldehydes, i.e., 85%, 79%, and 94% for chalcones **10**, **11**, and **13**, respectively (Table 3, entries 11, 12, and 15). For the trisubstituted benzaldehydes, the 3,4,5-trimethoxybenzaldehyde led to the corresponding chalcone **12** in 86% yield, while again, no reaction was observed for the 2,4,5-trimethoxybenzaldehyde under the optimal conditions (Table 3, entry 14).

Concerning this subfamily of chalcones, Albogami et al. [32] synthesized compound **13** in an 84% yield according to the previously mentioned procedure, while Szabados-Furjesi et al. [34] in 2018 and Detsi et al. in 2021 synthesized compound **11** in 88% and 50% yield, respectively [33]. Szabados-Furjesi et al. used a MeOH/aq. NaOH solution; after 1 h of reaction and one day at r.t. followed by acidic work-up, compound **11** was obtained in 88% yield.

### 2.3. Miscellaneous Synthesized Chalcones

**Figure 4:**
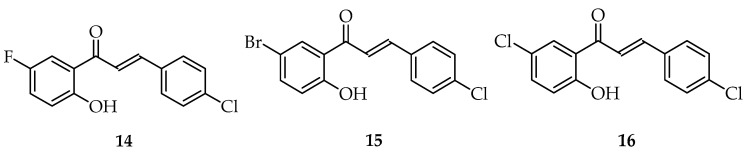
Chalcones **14**–**16** issued from 4-chlorobenzaldehyde.

In addition to this series of chalcones, we also examined the coupling between 5′-halogen-2′-hydroxy-substituted acetophenones and 4-chlorobenzaldehyde under ball mill conditions. Chalcones **14**, **15**, and **16** were obtained after 1 h of grinding in 85%, 81%, and 90% of yield when operating under the optimal conditions previously described (Figure 4). Compounds **15** and **16** were obtained in solution and under microwaveconditions by Albogami et al., with a reported yield of 89% and 92%, respectively [32].

**Figure 5:**
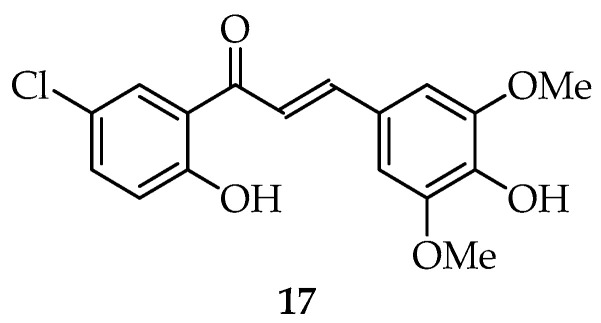
Chalcone **17** issued from selective deprotection of compound **8**.

Finally, for chalcone **8**, we investigated the selective deprotection of the 4-methoxy group. When operating in the presence of 6 eq. AlCl_3_ in methylene chloride and after 6 h of reaction, we exclusively obtained after purification the 4-hydroxy derivative **17** (Figure 5) in 72%.

### 2.4. Green Chemistry Parameters

In order to compare certain green chemistry parameters between reactions in solution and by mechanochemical means, we carried out the Claisen–Schmidt condensation reaction for the obtention of compound **12**, under ball milling, and in solution. Table 4 depicts our findings.

While the energy consumption was not greatly modified in favor of the ball milling conditions (30 W × h vs. 38 in solution), the yield of the reaction and the reaction time were much improved. The obtained yield was 86% under ball milling while only 47% when synthesizing compound **12** in solution, and the reaction time was two-fold less under mechanochemical conditions.

Finally, the E-factor developed by Sheldon [35] was calculated for both reactions. It is defined as follows:

E factor = total mass of waste/mass of product obtained

The synthetic route under our ball milling conditions afforded an E-factor almost half less than when operating in solution.

The mechanochemical synthesis of chalcones that we explored allowed us to obtain 16 chalcones in 60 min of grinding and 1 h of work-up in yields varying between 72% and 96%. Among them, eight chalcones (one 5′-fluoro-; three 5′-chloro-; two 5′-bromo-; and two 5-chloro-) have been reported in the literature to be obtained under conventional methods (solution) in yields varying between 50% and 92%. Thus, comparing the mechanochemical synthesis of 2′-hydroxychalcones we explored, we can assume that it is competitive with reactions in solution in terms of the time of reaction, time for work-up, E-factor parameter, yields and, subsequently, should be considered another option for their elaboration.

## 3. Biological Activities

The biological activities of all synthesized compounds were investigated regarding the development of two parasites, namely *Plasmodium falciparum* and *Leishmania donovani*, and the viability of IGR-39 human melanoma-derived cells, belonging to the most lethal and resistant cancer cell lines (RCCL) collection.

All biological results are presented in Table 5.

### 3.1. Antileishmanial Activities

#### 3.1.1. Effect of 2′-Hydroxychalcones on RAW 264.7 Cells

The cytotoxicity of the compounds was evaluated on the RAW 264.7 cells, and the CC_50_ values show these series are not toxic, with the lower CC_50_ value at 48 µM.

#### 3.1.2. Effect of 2′-Hydrochalcones on *L. donovani* LV9

The synthesized compounds were tested against MHOM/ET/67/HU3 (called LV9), *Leishmania donovani*, axenic amastigote forms, and intramacrophage amastigote forms.

Activities against the axenic forms

All compounds were first evaluated in vitro on the axenic form of *L. donovani*, the parasite responsible for visceral leishmaniasis in humans, to check their intrinsic antileishmanial activity. The reference drug is miltefosine (IC_50_ = 3.66 ± 0.73 µM). Except for compound **12**, which was completely inactive, the activities of the compounds were rather homogeneous with IC_50_ values in a range from 2 to 17 µM, so less than one log of difference. Considering chalcones **1**, **6**, and **10** possessing the 3,4-dimethoxy substitution pattern, the 5′-chloro derivative (compound **6**) possesses the best IC_50_ value (2.33 ± 0.53 µM), which is almost two-fold better than the 5′-bromo derivative **10**, and 8 times better than the fluorine one **1**. Concerning the 2,3-dimethoxy analogs (compounds **2**, **7**, and **11**), again, the 5′-fluoro derivative is less active than the 5′-chloro and 5′-bromo; the latter has the better IC_50_ value (2.82 ± 0.77 µM). The 3,5-dimethoxy analogues **5**, **9**, and **13** present the same activities; the same is true for the trimethoxy derivatives **3**, **4**, and **8,** while the 5′-bromo one (compound **12**) is inactive. The activities of the miscellaneous compounds **14**–**17** are less interesting, with IC_50_ values varying between 9.44 µM and 24.24 µM.

Activities against the intramacrophage amastigote forms

The compounds were then evaluated using the *L. donovani* intramacrophage amastigote model, which is closer to the pathological conditions. The IC_50_ value of miltefosine was 5.78 ± 1.02 µM, and except for compound **12**, the activities of the compounds ranged from 1 to 23 µM. Again, the most active compounds were the di-methoxylated derivatives **6**, **7**, and **11** (IC_50_ = 1.36 ± 0.51, 2.48 ± 0.42 and 3.29 ± 1.99 µM, respectively). It is important to point out that the di-chlorinated chalcone **15** also presented a potent IC_50_ value (2.87 ± 0.36 µM). The same compounds **6**, **7**, **11**, and **15** (Table 5) also have the highest selectivity indexes within this series (SI > 73; 40; 30; and 35, respectively).

### 3.2. Antiplasmodial Activities

All compounds were evaluated against the *P. falciparum* 3D7 strain. Some of the synthesized compounds showed interesting activity. Four of them (**2**, **6**, **7**, and **15**) have IC_50_ values < 10 µM meaning a three-log less activity than the reference mefloquine compound (12.27 ± 2.44 nM). It is interesting to point out that **6** and **7** are the most active against *L. donovani*, while compound **15** presents the best antiplasmodial activity (IC_50_ = 3.21 ± 0.61 µM) (Table 5).

### 3.3. Antimelanoma Activities

**Figure 6:**
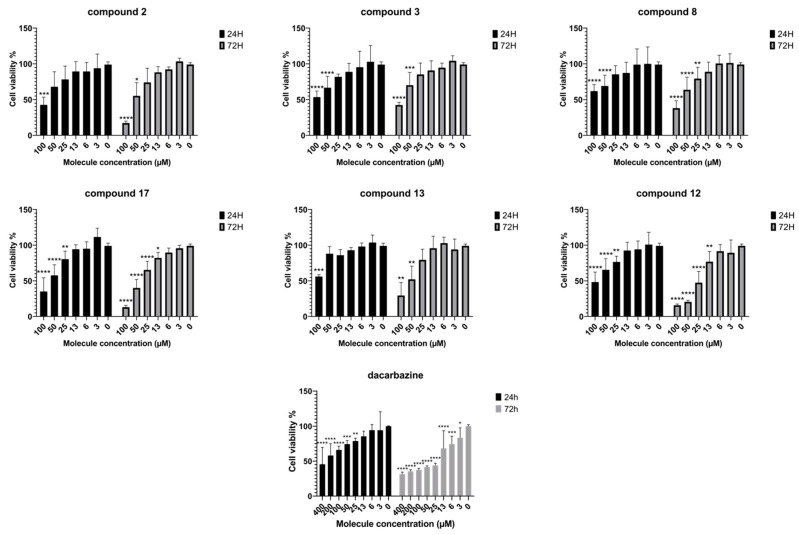
Concentration-dependent effects of 2′-hydroxychalcones and Dacarbazine on the viability of IGR-39 cells. SD values from three independent experiments performed in triplicate. A *p*-value < 0.05 (*), <0.01 (**), <0.001 (***), and <0.0001 (****) are considered to be statistically significant.

The synthesized compounds were evaluated by MTT test for their effects against the viability and proliferation of the IGR-39 human melanoma-derived cell line. The effects of the compounds were measured after treatment for 24 h and 72 h, respectively. Thus, different concentrations (3.125, 6.25, 12.5, 25, 50, and 100 µM) were used for each chalcone compound, and for the reference molecule dacarbazine (3.125, 6.25, 12.5, 50, 100, 200, and 400 µM), which is the chemotherapeutic molecule, clinically used against melanoma, the most invasive skin cancer. Among the 17 tested compounds (Table 5), 6 of them (**2**, **3**, **8**, **12**, **13**, and **17**) showed activities against melanoma cell viability (Figure 6). Interestingly, the effects of these chalcones were more important than dacarbazine after 24 h treatment. Indeed, at 100 µM, the chalcones displayed inhibition ranging from 65% to 39%, while the dacarbazine inhibited only 34% of IGR-39 cell viability (Figure 6), showing that these chalcones have a higher cytotoxic effect than the chemotherapeutic molecule. After 72 h treatment, the chalcones **12**, **13**, and **17** showed the best activities on the IGR-39 cells proliferation, with IC_50_ values of 24.7, 51.5 µM, and 38.3 µM, respectively, showing that they are potent molecules against IGR-39 melanoma cells development (Table 5). Interestingly, the most active compound, **12** (IC_50_ = 24.7 µM), showed almost the same effect as the dacarbazine (IC_50_ = 25.0 µM).

When compared to other reported chalcones tested on different melanoma cell lines, we found that compound **12** is two times more active than those of the ethoxy-chalcones class, studied by Harshitha et al. [36], where the best derivative showed an IC_50_ value of 53.47 µM for A-375 metastatic melanoma cell line. On the other hand, compound **12** was found to be less active than the synthesized chalcone–sulfonamide hybrids reported by Castaño et al. [37], most of them possessing activities against LOX IMVI melanoma cell line, with IC_50_ values between 0.34 and 0.54 μM.

Compound **12** has also shown a more important effect than the acridine chalcone 1C, reported by Gazdova et al. [38], on the proliferation of MCF-10A melanoma cell line (IC_50_ = 36.54 µM). Yet, the acridine chalcone 1C displayed a more important effect on other melanoma cell lines, like A2058 (IC_50_ = 7.96 µM) or BLM (IC_50_ = 17.93 µM), showing that its effect depends on the tested cell line. Thus, these results suggest that a comparison of chalcone’s effect should be carried out on the same cell line for an efficient QSAR study.

## 4. Conclusions

In this study, we highlighted the synthesis for the first time under mechanochemical conditions of a series of 2′-hydroxychalcones and their biological activities. The Claisen–Schmidt reaction was first chemically optimized in an MM400 apparatus in terms of base, additives, equivalents, time of reaction, and work-up. All compounds were obtained in very good to excellent yields.

A comparison of the synthesis of compound **12** was carried out in solution and under ball mill conditions, showing the interest of mechanochemistry in terms of green chemistry for the production of pharmaceutical molecules.

Thus, 17 compounds were synthesized and evaluated against *P. falciparum L. donovani* and human melanoma-derived cell line IGR-39, showing that some of them have promising therapeutic activity in the µM range.

Indeed, three compounds were active against *P. falciparum* 3D7 strain. Seven 2′-hydroxychalcones compounds showed important activities against *L. donovani*; four of them were very potent possessing also much higher selectivity indexes than the reference compound, miltefosine. Six compounds showed antiproliferative effects against the human melanoma-derived cell line IGR-39; and one of them was more active than the reference drug Dacarbazine.

Thus, considering our first very promising results, an extension of the library of 2′-hydroxychalcones by mechanochemical means and a fundamental study concerning their mechanochemical synthesis is underway. In addition, we will explore the signaling pathways involved in their activities, their potential in vivo effects, and their eventual synergic or additive effects with available drugs.

## 5. Materials and Methods

### 5.1. Synthesis and Characterization

All starting products were used in the solid phase, purchased from Sigma-Aldrich (St. Louis, MO, United States of America), Tokyo Chemical Industry (Tokyo, Japan) and Alfa Aesar (Haverhill, MA, United States of America) and were used as received without any further purification. The grinding device used was a Retsch Mixer Mill MM400 (Haan, Germany) composed of two jars in zirconium oxides with a volume of 10 mL each, containing two balls of 10 mm diameter with the same material. The grinding movement was a horizontal movement with a frequency of 30 Hz. The compounds were characterized by NMR spectroscopy performed in CDCl_3_, using the Bruker Avance III Nanobay 400 MHz (Billerica, MA, United States of America) 400 MHz for ^1^H and 101 MHz for ^13^C, with TMS as the internal standard. Chemical shifts (*δ*) are reported in parts per million (ppm) with respect to TMS. Data are represented as follows: chemical shifts, multiplicity (s = singlet, d = doublet, t = triplet, dd= doublet dedoublet, and m = multiplet), coupling constant (*J*, Hz), and integration. Mass spectrometry (MS) analyses were carried out on DSQII (Thermo Fisher Scientific, Waltham, MA, USA) using a DCI/ NH_3_ source. High-resolution mass spectrometry (HRMS) analyses were carried out on GCT Premier Waters (Milford, MA, United States of America) using a DCI/ NH4 source with a precision of 5 ppm. Melting points (mp) were obtained on a Stuart SMP3 melting point apparatus.

### 5.2. Biological Tests

#### 5.2.1. Evaluation of the Antileishmanial Activity

*L. donovani* (MHOM/ET/67/HU3, also called LV9) promastigotes and axenic amastigotes were maintained according to the protocols previously described [39,40].

In vitro antileishmanial evaluation on *L. donovani* axenic amastigotes. This evaluation was performed using the SYBR Green method, as previously described. IC_50_ values were calculated using the ICEstimator version 1.2 software (http://www.antimalarial-icestimator.net/ accessed on 10 December 2023). Miltefosine was used as the reference drug from Sigma-Aldrich (Merck, France).

In vitro antileishmanial evaluation on intramacrophage amastigotes. RAW 264.7 macrophages were infected with *L. donovani* axenic amastigotes according to a ratio of 10 parasites per macrophage. In these conditions, the percentage of infected macrophages was around 80%, and the mean number of amastigotes per infected macrophage was 4 to 5 in the untreated controls. The in vitro treatment was applied 24 h post-infection, and the treatment duration was 72 h. The results of the effect of the compounds are given as a percentage of parasite growth reduction, measured using the SYBR Green incorporation method. The activity of the compounds is expressed as IC_50_, calculated using the ICEstimator version 1.2 software. Miltefosine was used as the reference drug.

#### 5.2.2. Evaluation of the Antimalarial Activity on P. 3D7 Strain

The P. 3D7 strain obtained by Malaria Research and reference reagent center (MR4) was maintained in O^+^ human erythrocytes inGibco™ RPMI 1640 medium (Life technologies, France) complemented with 2.5 mM HEPES, 2.5 mM NaHCO_3_, 10% AB Human serum and incubated at 37 °C and 5% CO_2_ and 10% O_2_. The parasites were synchronized to the ring stage by repeated sorbitol treatment to obtain at least 85% ring stage. A suspension with 2.5% hematocrit and 1% parasitemia was incubated with the test compounds dissolved in DMSO. Parasites were also incubated with culture medium (growth control) or with different concentrations of compounds in 96-well culture plates. The compounds and mefloquine (drug control) were evaluated at concentrations between 97.6 nM and 100 µM for compounds and 0.5 nM and 1 µM for mefloquine by making a serial dilution. The mefloquine was provided by the Worldwide Antimalarial Resistance Network (WWARN Network). After 44 h incubation at 37 °C, the hemolysis was controlled to eliminate the molecules causing lysis of the red blood cells. The results of the effect of the compounds are given as a percentage of parasite growth reduction, measured using the SYBR Green incorporation method as previously.

#### 5.2.3. Antimelanoma Tests against IGR-39 Cell Culture

IGR-39 melanoma cells were generously provided by Pr. José Luis from CNRS UMR 7051, Institut de Neuro-Physiopathologie, Faculty of Medecine (La Timone, Marseille). Cells were cultured on DMEM (Dulbelcco’s Modified Eagle Medium) supplemented by 10% FBS (Fetal Bovine Serum), 1% antibiotic (Penicillin and Streptomycin 100 UI/mL), and 1% L-Glutamine and maintained on humidified incubator at 37 °C (5% CO_2_).

Cell viability and proliferation assay

IGR-39 cells were seeded in two 96 well-plates at densities of 10,000 or 5000 cells per well and incubated for 18 h at 37 °C. Then, the cells were treated with different concentrations of 2′-hydroxychalcones or dacarbazine (as positive control) and incubated for 24 and 72 h (proliferation), respectively. At the end of incubation, new culture media containing 0.5 mg/mL of MTT (3-(4,5-dimethylthiazol-2-yl)-2,5-diphenyltetrazolium bromide) were added, and the plates were re-incubated for 3 h. Afterward, the culture media were replaced by a DMSO solution to dissolve the formazon crystal, and the optical density (OD) was measured at 560 nm.

Statistical analysis

Data were analyzed by GraphPad Prism program 8.4.3, using two-way ANOVA. Results were represented as mean ± SD values from three independent experiments performed in triplicate. A *p*-value < 0.05 (*), <0.01 (**), <0.001 (***), and <0.0001 (****) were considered to be statistically significant.

### 5.3. Experimental Results in Chemistry

#### 5.3.1. General Procedure for the 2′-Hydroxychalcone Derivatives Synthesis

Different chalcones were prepared, starting with an equimolar medium from a substituted 2′-hydroxyacetophenone and a substituted benzaldehyde (1.2 mmol) in the presence of 2 eq. KOH. Two grinding cycles of 30 min, with the addition of one equivalent of the benzaldehyde derivative between cycles, allowed a red powder to be obtained, which was dissolved in 10 mL cold MeOH and acidified with 2 mL cold HCl (1 M) till pH = 3. The formed yellow precipitate was then filtered, washed, and analyzed by ^1^H NMR, ^13^C NMR, and mass spectroscopy.

#### 5.3.2. General Procedure for the Synthesis of Compound **12** in the Conventional Method

Compound **12** was synthesized in solution in order to compare the green chemistry parameters between this method and the mechanochemistry synthesis method. For this, we prepared an equimolar mixture of the starting material, diluted in 50 mL Ethanol on which 2 eq. of KOH were added. The mixture was stirred for 2 h under a reflux of 50 °C. The mixture was then cooled progressively until room temperature, 30 mL of ethanol was added, and then the solution was acidified with 2 mL of HCl (1 M), forming a precipitate that was filtered and washed with water. The crud was analyzed by ^1^H and ^13^C NMR, showing compound **12**.

*5′-fluoro-2′-hydroxy-3*,*4-dimethoxy-Chalcone* (**1**). Yellow powder, 96% yield; mp 127.5 °C; ^1^H NMR (400 MHz, CDCl_3_; *δ*, ppm) 12.65 (s, OH), 7.91 (d, *J* = 15.4 Hz, 1H), 7.60 (dd, *J* = 9.2, 3 Hz, 1H), 7.40 (d, *J* = 15.4 Hz, 1H), 7.27–7.30 (m, 1H), 7.21–7.24 (m, 1H), 7.18 (s, 1H), 7.00 (dd, *J* = 9.1, 4.6 Hz, 1H), 6.93 (d, *J* = 8.3 Hz, 1H), 3.98 (s, 3H), 3.95 (s, 1H); ^13^C {^1^H} NMR (101 MHz, CDCl_3_; *δ*, ppm) 192.69 (C=O), 159.7 (C-OH), 154.85 (d, *J* = 238.44 Hz, C-F), 152.10, 149.4, 146.6, 127.35, 124, 123.66 (d, *J* = 23.6 Hz), 119.83 (d, *J* = 7.3 Hz), 119.62 (d, *J* = 6.2 Hz), 117.16, 114.48 (d, *J* = 23.3 Hz), 111.2, 110.23, 56.08 (C-OMe), 56.07 (C-OMe); MS (DCI-NH_3_) *m*/*z* calculated for C_17_H_15_FO_4_, theoretical for [M]^+^: 302.1, found 302.9. HRMS (DCI-CH_4_) *m*/*z* calculated for C_17_H_15_FO_4_, theoretical for [M + H]^+^: 303.1033, found: 303.1033, theoretical for [M + C_2_H_5_]^+^: 331.1346, found: 332.1405, theoretical for [M + C_3_H_5_]^+^: 343.1346, found: 343.1410.

*5′-fluoro-2′-hydroxy-2,3-dimethoxy-Chalcone* (**2**). Yellow powder, 74% yield; mp 120.2 °C; ^1^H NMR (400 MHz, CDCl_3_; δ, ppm) 12.57 (s, OH), 8.23 (d, *J* = 15.6 Hz, 1H), 7.64 (d, *J* = 15.6 Hz, 1H), 7.58 (dd, *J* = 9.1, 3.1 Hz, 1H), 7.29 (dd, *J* = 8, 1.5 Hz, 1H), 7.30–7.22 (m, 1H), 7.13 (t, *J* = 8 Hz, 1H), 7.03–7.01 (m, 1H), 7.00–6.98 (m, 1H), 3.93 (s, 3H), 3.91 (s, 3H); ^13^C {^1^H} NMR (101 MHz, CDCl_3_; δ, ppm) 193.26 (C=O), 159.76 (C-OH), 154.85 (d, *J* = 238.1 Hz, C-F), 153.28, 149.32, 141.34, 128.53, 124.30, 123.82 (d, *J* = 23.7 Hz), 121.07, 119.94, 119.82 (d, *J* = 7.1 Hz), 119.64, 114.87, 114.60 (d, *J* = 23.7 Hz), 65.95 (C-OMe), 61.41 (C-OMe); MS (DCI-NH_3_) *m*/*z* calculated for C_17_H_15_FO_4_, theoretical for [M]^+^: 302.1, found 303.0. HRMS (DCI-CH_4_) *m*/*z* calculated for C_17_H_15_FO_4_, theoretical for [M + H]^+^: 303.1033, found: 303.1033.

*5′-fluoro-2′-hydroxy-3,4,5-trimethoxy-Chalcone* (**3**). Yellow powder, 84% yield; mp 123.2 °C; ^1^H NMR (400 MHz, CDCl_3_; *δ*, ppm) 12.56 (s, OH), 7.87 (d, *J* = 15.3 Hz, 1H), 7.60 (dd, *J* = 9.1, 3.1 Hz, 1H), 7.41 (d, *J* = 15.3 Hz, 1H), 7.28–7.23 (m, 1H), 7.01 (dd, *J* = 9.2, 4.6 Hz, 1H), 6.89 (s, 2H), 3.95 (s, 6H), 3.92 (s, 3H); ^13^C {^1^H} NMR (101 MHz, CDCl_3_; *δ*, ppm) 192.64 (C=O), 159.75 (C-OH), 154.86 (d, *J* = 238.2 Hz, C-F), 153.58, 146.58, 141.12, 129.77, 128.87 (d, *J* = 23.6 Hz), 119.52 (d, *J* = 6.1 Hz), 118.64, 114.51 (d, *J* = 23.6 Hz), 106.09, 61.07 (2 × C-OMe), 56.23 (C-OMe); MS (DCI-NH_3_) *m*/*z* calculated for C_18_H_17_FO_5_, theoretical for [M]^+^: 332.1, found: 333.0. HRMS (DCI-CH_4_) *m*/*z* calculated for C_18_H_17_FO_5_, theoretical for [M + H]^+^: 333.1138, found: 333.1140, theoretical for [M + C_2_H_5_]^+^: 361.1451, found: 361.1454, theoretical for [M + C_3_H_5_]^+^: 373.1451, found: 373.1443.

*5′-fluoro-2′-hydroxy-2,4,5-trimethoxy-Chalcone* (**4**). Yellow powder, 72% yield; mp 143 °C; ^1^H NMR (400 MHz, CDCl_3_; *δ*, ppm) 12.81 (s, OH), 8.24 (d, *J* =15.5 Hz, 1H), 7.59 (dd, *J* = 9.2, 3.1 Hz, 1H), 7.51 (d, *J* = 15.5 Hz, 1H), 7.22 (ddd, *J* = 9.1, 7.8, 3.1 Hz, 1H), 7.12 (s, 1H), 6.98 (dd, *J* = 9.1, 4.6 Hz, 1H), 6.53 (s, 1H), 3.97 (s, 3H), 3.95 (s, 3H), 3.93 (s, 3H); ^13^C {^1^H} NMR (101 MHz, CDCl_3_; *δ*, ppm), 193.16 (C=O), 159.67 (C-OH), 154.81 (d, *J* = 241.74 Hz, C-F), 155.37, 153.31, 143.36, 141.90, 123.3 (d, *J* = 23.6 Hz), 119.68 (d, *J* = 7.3 Hz), 117.23, 114.96, 114.49 (d, *J* = 23.2 Hz),118.83, 96.60, 56.62 (C-OMe), 56.34 (C-OMe), 56.14 (C-OMe); ^19^F {^1^H} NMR (400 MHz, CDCl_3_; *δ*, ppm) 124.37; MS (DCI-NH_3_) *m*/*z* calculated for C_18_H_17_FO_5,_ theoretical for [M]^+^: 332.1, found 333.0. HRMS (DCI-CH_4_) *m*/*z* calculated for C_18_H_17_FO_5_, theoretical for [M + H]^+^: 333.1138, found: 333.1133, theoretical for [M + C_2_H_5_]^+^: 361.1451, found 361.1494; theoretical for [M + C_3_H_5_]^+^: 373.1451, found: 373.1425.

*5′-fluoro-2′-hydroxy-3,5-dimethoxy-Chalcone* (**5**). Yellow powder, 88% yield; mp 130 °C; ^1^H NMR (400 MHz, CDCl_3_; *δ*, ppm) 12.50 (s, OH), 7.86 (d, *J* = 15.4 Hz, 1H), 7.58 (dd, *J* = 9.1, 3.1 Hz, 1H), 7.49 (d, *J* = 15.4 Hz, 1H), 7.28–7.22 (m, 1H), 7.00 (dd, *J* = 9.2,4.6 Hz, 1H), 6.8 (d, *J* = 2.3 Hz, 2H), 6.57 (t, *J* = 2.3 Hz, 1H), 3.86 (s, 6H); ^13^C {^1^H} NMR (101 MHz, CDCl_3_; *δ*, ppm) 192.81 (C=O), 161.16, 159.76 (C-OH), 154.86 (d, *J* = 238,1 Hz, C-F), 146.39, 136.18, 123.97 (d, *J* = 23.7 Hz), 120.02, 119.98 (d, *J* = 7.3 Hz), 114.57 (d, *J* = 23.3 Hz), 106.67, 103.38, 55.54 (2 × C-OMe); MS (DCI-NH_3_) *m*/*z* calculated for C_17_H_15_FO_4_, theoretical for [M]^+^: 302.1, found 303.0. HRMS (DCI-CH_4_) *m*/*z* calculated for C_17_H_15_FO_4_, theoretical for [M + H]^+^: 303.1033, found: 303.1027, theoretical for [M + C_2_H_5_]^+^: 331.1346, found: 331.1348, theoretical for [M + C_3_H_5_]^+^: 343.1346, found: 343.1371.

*5′-chloro-2′-hydroxy-3,4-dimethoxy-Chalcone* (**6**). Yellow powder, 92% yield; mp 149.4 °C; ^1^H NMR (400 MHz, CDCl_3_; *δ*, ppm) 12.84 (s, OH), 7.92 (d, *J* = 15.6 Hz, 1H), 7.88 (d, *J* = 3.3 Hz, 1H), 7.44 (t, *J* = 4.4 Hz, 1H), 7.41 (d, *J* = 7.4 Hz, 1H), 7.30 (dd, *J* = 8.4, 2 Hz, 1H), 7.19 (d, *J* = 2 Hz, 1H), 6.99 (d, *J* = 8.9 Hz, 1H), 6.93 (d, *J* = 8.3 Hz, 1H), 3.99 (s, 3H), 3.96 (s, 3H); ^13^C {^1^H} NMR (101 MHz, CDCl_3_; *δ*, ppm) 192.63 (C=O), 162.04 (C-OH), 152.16, 149.40, 146.81, 135.94, 128.69, 127.34, 124.04, 123.39, 120.72, 120.22, 117.02, 111.19, 110.37, 56.12 (C-OMe), 56.07 (C-OMe); MS (DCI-NH_3_) *m*/*z* calculated for C_17_H_15_ClO_4_, theoretical for [M]^+^: 318.1, found 317.9. HRMS (DCI-CH_4_) *m*/*z* calculated for C_17_H_15_ClO_4_, theoretical for [M + H]^+^: 319.0737, found: 319.0730, theoretical for [M + C_2_H_5_]^+^: 347.1050, found: 347.1039, theoretical for [M + C_3_H_5_]^+^: 359.1050, found: 359.1202.

*5′-chloro-2′-hydroxy-2,3-dimethoxy-Chalcone* (**7**). Yellow powder, 95% yield; mp 104 °C; ^1^H NMR (400 MHz, CDCl_3_; δ, ppm) 12.75 (s, OH), 8.24 (d, *J* = 15.6 Hz, 1H), 8.87 (d, *J* = 2.6 Hz, 1H), 7.66 (d, *J* = 15.6 Hz, 1H), 7.44 (dd, *J* = 8.9, 2.5 Hz, 1H), 7.31 (dd, *J* = 8.0, 1.5 Hz, 1H), 7.13 (t, *J* = 8.0 Hz, 1H), 6.99 (d, *J* = 8.8 Hz, 1H), 7.03 (d, *J* = 1.5 Hz, 1H), 3.93 (s, 3H), 3.91 (s, 3H);^13^C {^1^H} NMR (101 MHz, CDCl_3_; δ, ppm) 193.20 (C=O), 162.06 (C-OH), 153.28, 149.33, 141.47, 136.10, 128.91, 128.52, 124.32, 123.50, 120.96, 120.72, 120.21, 119.90, 114.91, 61.45 (C-OMe), 55.95 (C-OMe); MS (DCI-NH_3_) *m*/*z* calculated for C_17_H_15_ClO_4_, theoretical for [M]^+^: 318.1, found 317.8. HRMS (DCI-CH_4_) *m*/*z* calculated for C_17_H_15_ClO_4_, theoretical for [M + H]^+^: 319.0737, found: 319.0737, theoretical for [M + C_2_H_5_]^+^: 347.1050, found: 347.1048, theoretical for [M + C_3_H_5_]^+^: 359.1050, found: 359.1079.

*5′-chloro-2′-hydroxy-3,4,5-trimethoxy-Chalcone* (**8**). yellow powder, 88% yield; mp 147.2 °C; ^1^H NMR (400 MHz, CDCl_3_; *δ*, ppm) 12.76 (s, OH), 7.89 (d, *J* = 4.7 Hz, 1H), 7.87 (d, *J* = 8.1 Hz, 1H), 7.44–7.47 (m, 1H), 7.43 (d, *J* = 9.4 Hz, 1H), 7.00 (d, *J* = 8.9 Hz, 1H), 6.9 (s, 2H), 3.96 (s, 6H), 3.93 (s, 3H); ^13^C {^1^H} NMR (101 MHz, CDCl_3_; *δ*, ppm) 192.60 (C=O), 162.09 (C-OH), 153.59, 146.81, 136.14, 129.74, 128.73, 123.46, 120.29, 120.22, 118.49, 117.71, 106.21, 61.06 (C-OMe), 56.37 (2 × C-OMe); MS (DCI-NH_3_) *m*/*z* calculated for C_18_H_17_ClO_5_, theoretical for [M]^+^: 348.1; found 347.8; HRMS (DCI-CH_4_) *m*/*z* calculated for C_18_H_17_ClO_5_, theoretical for [M + H]^+^: 349.0843, found: 349.0833, theoretical for [M + C_2_H_5_]^+^: 377.1156, found: 377.1147; theoretical for [M + C_3_H_5_]^+^: 389.1156, found: 389.1089.

*5′-chloro-2′-hydroxy-3,5-dimethoxy-Chalcone* (**9**). Yellow powder, 94% yield; mp 125.5 °C; ^1^H NMR (400 MHz, CDCl_3_; *δ*, ppm) 12.69 (s, OH), 7.88 (d, *J* = 4 Hz, 1H), 7.85 (d, *J* = 8.8 Hz, 1H), 7.52 (d, *J* = 15.4 Hz, 1H), 7.45 (dd, *J* = 8.9, 2.5 Hz, 1H); 7.00 (d, *J* = 8.9 Hz, 1H), 6.81 (d, *J* = 2.2 Hz, 2H), 6.57 (t, *J* = 2.2 Hz, 1H), 3.87 (s, 6H); ^13^C {^1^H} NMR (101 MHz, CDCl_3_; *δ*, ppm) 192.79 (C=O), 162.07 (C-OH), 161.17, 146.59, 136.25, 136.16, 128.83, 120.26, 119.93, 106.75, 103.40, 55.17 (2 × C-OMe); MS (DCI-NH_3_) *m*/*z* calculated for C_17_H_15_ClO_4_ theoretical for [M]^+^: 318.1; found 318.9. HRMS (DCI-CH_4_) *m*/*z* calculated for C_17_H_15_ClO_4_, theoretical for [M + H]^+^: 319.0737, found: 319.0734, theoretical for [M + C_2_H_5_]^+^: 347.1050, found: 347.1056, theoretical for [M + C_3_H_5_]^+^: 359.1050, found: 359.1053.

*5′-bromo-2′-hydroxy-3,4-dimethoxy-Chalcone* (**10**). yellow powder, 85% yield; mp 147.9 °C; ^1^H NMR (400 MHz, CDCl_3_; *δ*, ppm) 12.87 (s, OH), 8.01 (s, *J* = 2.32 Hz, 1H), 7.91 (d, *J* = 15.2 Hz, 1H), 7.56 (dd, *J* = 8.9, 2.4 Hz, 1H), 7.41 (d, *J* = 15.3 Hz, 1H), 7.30 (dd, *J* = 8.4, 2 Hz, 1H), 7.18 (d, *J* = 4 Hz, 1H), 6.94 (dd, *J* = 8.6, 3 Hz, 1H), 3.99 (s, 3H), 3.95 (s, 3H); ^13^C {^1^H} NMR (101 MHz, CDCl_3_; *δ*, ppm) 192.56 (C=O), 162.49 (C-OH), 152.18, 149.41, 146.87, 138.71, 131.69, 127.34, 124.04, 121.38, 120.64, 117.01, 111.19, 110.48, 110.32, 56.15 (C-OMe), 56.08 (C-OMe); MS (DCI-NH_3_) *m*/*z* calculated for C_17_H_15_BrO_4_, theoretical for [M]^+^: 362.0 and 364.0 found 361.8 and 363.8. HRMS (DCI-CH_4_) *m*/*z* calculated for C_17_H_15_BrO_4_, theoretical for [M + H]^+^: 363.0232 and 365.0211 found: 363.0233 and 365.0217, theoretical for [M + C_2_H_5_]^+^: 391.0545 and 393.0524, found: 391.0567 and 393.0513.

*5′-bromo-2′-hydroxy-2,3-dimethoxy-Chalcone* (**11**). yellow powder, 79% yield; mp 122.6 °C; ^1^H NMR (400 MHz, CDCl_3_; *δ*, ppm) 12.78 (s, OH), 8.23 (d, *J* = 15.6 Hz, 1H), 8.00 (d, *J* = 2.4 Hz, 1H), 7.65 (d, *J* = 15.6 Hz, 1H), 7.57 (dd, *J* = 8.9, 2.4 Hz, 1H), 7.31 (dd, *J* = 7.9, 1.1 Hz, 1H), 7.13 (t, *J* = 8 Hz, 1H), 7.02 (dd, *J* = 8.2, 1.5 Hz, 1H), 6.94 (d, *J* = 8.9 Hz, 1H), 3.93 (s, 3H), 3.91 (s, 3H); ^13^C {^1^H} NMR (101 MHz, CDCl_3_; *δ*, ppm) 193.13 (C=O), 162.49 (C-OH), 153.27, 149.32, 141.49, 138.86, 131.94, 128.51, 124.31, 121.36, 120.95, 120.61, 119.90, 114.91, 110.41, 61.45 (C-OMe), 55.94 (C-OMe);MS (DCI-NH_3_) *m*/*z* calculated for C_17_H_15_BrO_4_, theoretical for [M]^+^: 362.0 and 364.0, found 361.8 and 363.9. HRMS (DCI-CH_4_) *m*/*z* calculated for C_17_H_15_BrO_4_, theoretical for [M + H]^+^: 363.0232 and 365.0211 found: 363.0234 and 365.0215, theoretical for [M + C_2_H_5_]^+^: 391.0545 and 393.0524, found: 391.0533 and 393.0529, theoretical for [M + C_3_H_5_]^+^: 403.0545 and 405.0524 found: 403.0604 and 405.0453.

*5′-bromo-2′-hydroxy-3,4,5-trimethoxy-Chalcone* (**12**). yellow powder, 86% yield; mp 125.9 °C; ^1^H NMR (400 MHz, CDCl_3_; *δ*, ppm) 12.79 (s, OH), 8.01 (d, *J* = 2.4 Hz, 1H), 7.87 (d, *J* = 15.3 Hz, 1H), 7.57 (dd, *J* = 8.9, 2.4 Hz, 1H), 7.42 (d, *J* = 15.3 Hz, 1H), 6.95 (d, *J* = 8.9 Hz, 1H), 6.90 (s, 2H), 3.96 (s, 6H), 3.93 (s, 3H); ^13^C {^1^H} NMR (101 MHz, CDCl_3_; *δ*, ppm) 192.54 (C=O), 162.52 (C-OH), 153.58, 146.87, 141.22, 138.91, 131.72, 129.73, 121.28, 120.69, 118.47, 110.39, 106.25, 61.06 (C-OMe), 56.38 (2 × C-OMe); MS (DCI-NH_3_) *m*/*z* calculated for C_18_H_17_BrO_5_, theoretical for [M]^+^: 394.0 and 392.0; found 391.8 and 393.8; HRMS (DCI-CH_4_) *m*/*z* calculated for C_18_H_17_BrO_5_, theoretical for [M + H]^+^: 393.0338 and 395.0317 found: 393.0327 and 395.0322, theoretical for [M + C_2_H_5_]^+^: 421.0651 and 423.0630, found: 421.0667 and 423.0651, theoretical for [M + C_3_H_5_]^+^: 433.0651 and 435.0630, found: 433.0654 and 435.0612.

*5′-bromo-2′-hydroxy-3,5-dimethoxy-Chalcone* (**13**). yellow powder, 94% yield; mp 129 °C; ^1^H NMR (400 MHz, CDCl_3_; *δ*, ppm) 12.72 (s, OH), 8.00 (d, *J* = 2.4 Hz, 1H), 7.86 (d, *J* = 15.4 Hz, 1H), 7.58 (dd, *J* = 8.9, 2.4 Hz, 1H), 7.50 (d, *J* = 15.4 Hz, 1H), 6.94 (d, *J* = 8.9 Hz, 1H), 6.81 (d, *J* = 2.3 Hz, 2H), 6.57 (t, *J* = 2.2 Hz, 1H), 3.87 (s, 6H); ^13^C {^1^H} NMR (101 MHz, CDCl_3_; *δ*, ppm); 192.72 (C=O), 162.51 (C-OH), 161.17, 146.63, 139.01, 136.16, 131.84, 121.23, 120.66, 119.91, 110.46, 106.78, 103.38, 55.58 (2 × C-OMe); MS (DCI-NH_3_) *m*/*z* calculated for C_17_H_15_BrO_4_, theoretical for [M]^+^: 362.0 and 364.0, found 361.8 and 363.8. HRMS (DCI-CH_4_) *m*/*z* calculated for C_17_H_15_BrO_4_, theoretical for [M + H]^+^: 363.0232 and 365.0211 found: 363.0221 and 365.0208, theoretical for [M + C_2_H_5_]^+^: 391.0545 and 393.0524, found: 391.0517 and 393.0517, theoretical for [M + C_3_H_5_]^+^: 403.0545 and 405.0524 found: 403.0560 and 405.0560.

*5-Chloro-5′-fluoro-2′-hydroxy-Chalcone* (**14**). yellow powder, 85% yield; mp 171.3 °C; ^1^H NMR (400 MHz, CDCl_3_; *δ*, ppm) 12.47 (s, OH), 8.03 (d, *J* = 8.6 Hz, 1H), 7.90 (d, *J* = 15.5 Hz, 1H), 7.61 (d, *J* = 8.4 Hz, 2H), 7.57 (dd, *J* = 9, 3.1 Hz, 1H), 7.52 (d, *J* = 15.5 Hz, 2H), 7.43 (d, *J* = 8.5 Hz, 2H), 7.01 (dd, *J* = 9.01, 4.6 Hz, *1H*); ^13^C {^1^H} NMR (101 MHz, CDCl_3_; *δ*, ppm) 192.64 (C=O), 159.76 (C-OH), 156.06, 153.69, 144.81, 137.22, 132.82, 129.91, 129.43, 124.22, 123.99, 119.92, 114.37; MS (DCI-NH_3_) *m*/*z* calculated for C_15_H_10_FClO_2_, theoretical for [M]^+^: 276.0; found 277.1; HRMS (DCI-CH_4_) *m*/*z* calculated for C_15_H_10_FClO_2_, theoretical for [M + H]^+^: 277.0432, found: 277.0416, theoretical for [M + C_2_H_5_]^+^: 305.0745, found: 305.0751, theoretical for [M + C_3_H_5_]^+^: 317.0745, found: 317.0845.

*5′-bromo-5-chloro-2′-hydroxy-Chalcone* (**15**). yellow powder, 90% yield; mp 123.3 °C; ^1^H NMR (400 MHz, CDCl_3_; *δ*, ppm) 12.68 (s, OH), 7.99 (d, *J* = 2.4 Hz, 1H), 7.89 (d, *J* = 15.4 Hz, 1H), 7.63 (d, *J* = 8.5 Hz, 2H), 7.58 (dd, *J* = 8.9, 2.4 Hz, 1H), 7.54 (d, *J* = 15.4 Hz, 1H), 7.43 (d, *J* = 8.5 Hz, 2H), 6.95 (d, *J* = 8.9 Hz, *1H*); ^13^C {^1^H} NMR (101 MHz, CDCl_3_; *δ*, ppm) 192.56 (C=O), 162.10 (C-OH), 144.98, 137.27, 136.34, 132.79, 129.97, 129.44, 128.76, 123.61, 120.51, 120.32, 119.88 (C-Br); MS (DCI-NH_3_) *m*/*z* calculated for C_15_H_10_BrClO_2_, theoretical for [M]^+^: 336.0 and 338.0, found 337.0 and 339.0. HRMS (DCI-CH_4_) *m*/*z* calculated for C_15_H_10_BrClO_2,_ theoretical for [M + H]^+^: 336.9631 and 338.9610, found: 336.0921 and 338.9606, theoretical for [M + C_2_H_5_]^+^: 364.9944 and 366.9923, found: 364.9953 and 366.9926.

*5,5′-dichloro-2′-hydroxy-Chalcone* (**16**). yellow powder, 81% yield; mp 167.7 °C; ^1^H NMR (400 MHz, CDCl_3_; *δ*, ppm) 12.65 (s, OH), 7.89 (d, *J* = 15.4 Hz, 1H), 7.85 (d, *J* = 2.6 Hz, 1H), 7.62 (d, *J* = 8.5 Hz, 2H), 7.54 (d, *J* = 15.4 Hz, 1H), 7.46 (d, *J* = 2.6 Hz, 1H), 7.43 (d, *J* = 6.8 Hz, 2H), 7.00 (d, *J* = 8.9 Hz, *1H*); ^13^C {^1^H} NMR (101 MHz, CDCl_3_; *δ*, ppm) 192.48 (C=O), 162.53 (C-OH), 145.03, 139.11, 137.29, 132.78, 131.78, 129.99, 129.44, 121.16, 120.72, 119.86, 110.50; MS (DCI-NH_3_) *m*/*z* calculated for C_15_H_10_Cl_2_O_2_, theoretical for [M]^+^: 292.0, found 293.0; HRMS (DCI-CH_4_) *m*/*z* calculated for C_15_H_10_Cl_2_O_2_, theoretical for [M + H]^+^: 293.0136, found: 293.0126, theoretical for [M + C_2_H_5_]^+^: 321.0449, found: 321.0450; theoretical for [M + C_3_H_5_]^+^: 333.0449, found: 333.0443.

*5′-chloro-2′,4-dihydroxy-3,5-dimethoxy-Chalcone* (**17**). yellow powder, 72% yield; mp 187.4 °C; ^1^H NMR (400 MHz, CDCl_3_; *δ*, ppm) 12.82z (s, OH), 7.89 (d, *J* = 15.4 Hz, 1H), 7.85 (d, *J* = 2.6 Hz, 1H), 7.62 (d, *J* = 8.5 Hz, 2H), 7.54 (d, *J* = 15.4 Hz, 1H), 7.46 (d, *J* = 2.6 Hz, 1H), 7.43 (d, *J* = 6.8 Hz, 2H), 7.00 (d, *J* = 8.9 Hz, *1H*); ^13^C {^1^H} NMR (101 MHz, CDCl_3_; *δ*, ppm) 192.52 (C=O), 162.05 (C-OH), 147.36, 147.23, 138.32, 135.96, 128.66, 125.88, 123.38 (2 × C-OMe), 120.71, 120.24, 117.02, 106.09 (2 × CH), 56.54 (2 × H_3_C-O); MS (DCI-NH_3_) *m*/*z* calculated for C_17_H_15_ClO_5_, theoretical for [M]^+^: 334.1, found 335.1; HRMS (DCI-CH_4_) *m*/*z* calculated for C_17_H_15_ClO_5_, theoretical for [M + H]^+^: 335.0686, found: 335.0677, theoretical for [M + C_2_H_5_]^+^: 363.0999, found: 363.0998; theoretical for [M + C_3_H_5_]^+^: 375.0999, found: 375.0948.

## Figures and Tables

**Table 1:** Conditions for the model mechanochemical Claisen–Schmidt reaction.

Entry	Base	Eq.: Ketone: Aldehyde: Base	Time Cycle × min	Additive (1 or 3 eq.)	Yields (%)
1	NaOH	1:1:1	2 × 15	-	No reaction
2	NaOH	1:1:2	2 × 15	-	20
3	NaOH	1:1:3	2 × 15	-	23
4	LiOH	1:1:3	2 × 15	-	No reaction
5	KOH	1:1:2	2 × 15	-	40
6	KOH	1:1:3	2 × 15	-	43
7	KOH	1:1:2	2 × 30	-	78
8	KOH	1:1 + 1:2	2 × 30	-	96
9	KOH	1:1:2	2 × 15	K_2_CO_3_	54
10	KOH	1:1:2	1 × 60	1 eq. K_2_CO_3_ 3 eq. K_2_CO_3_	74 76
11	KOH	1:2:2	1 × 60	K_2_CO_3_	95
12	KOH	1:1:2	2 × 15	1 eq. Alumina 3 eq. Alumina	-

**Table 2:**
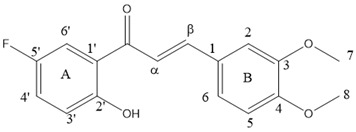
^1^H and ^13^C NMR chemical shifts (ppm) of compound **1**, fluorine decoupled in CDCl_3_.

^1^H and/or ^13^C	^1^H Chemical Shift	^13^C Chemical Shift
C=O		192.69
CH-α	7.40	117.16
CH-β	7.91	146.60
C-1		127.35
CH-2	7.18	110.23
C-3		149.40
C-4		152.10
CH-5	6.93	111.2
CH-6	7.28	124.0
C-1′		119.62
C-2′		159.7
CH-3′	7.00	119.83
CH-4′	7.23	123.66
C-5′		154.85
CH-6′	7.60	114.48
CH_3_-7	3.98	56.07
CH_3_-8	3.95	56.09

**Table 3:** Reagents used with chalcones and yields obtained. For positions of residues R_2_–R_5_, see Figure 3.

Entry	Ketone X = F, Cl, Br	Benzaldehyde	Chalcone	Yield (%)
1	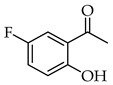	R_3_, R_4_ = OMe; R_2_, R_5_ = H	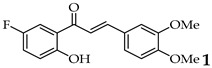	96
2		R_2_, R_3_ = OMe R_4_, R_5_ = H	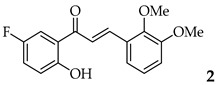	74
3		R_3_, R_4_, R_5_ = OMe R_2_ = H	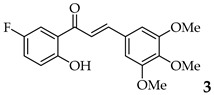	84
4		R_2_, R_4_, R_5_ = OMe R_3_ = H	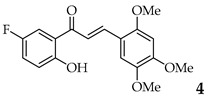	72
5		R_3_, R_5_ = OMe R_2_, R_4_ = H	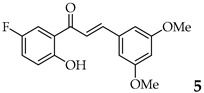	88
6	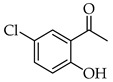	R_3_, R_4_ = OMe R_2_, R_5_ = H	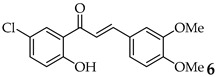	92
7		R_2_, R_3_ = OMe R_4_, R_5_ = H	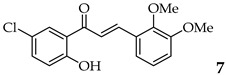	95
8		R_3_, R_4_, R_5_ = OMe R_2_ = H	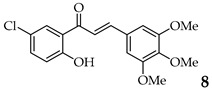	88
9		R_2_, R_4_, R_5_ = OMe	--	No reaction
10		R_3_, R_5_ = OMe R_2_, R_4_ = H	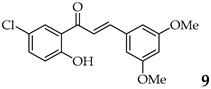	94
11	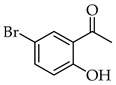	R_3_, R_4_ = OMe R_2_, R_5_ = H	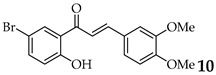	85
12		R_2_, R_3_ = OMe R_4_, R_5_ = H	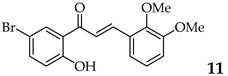	79
13		R_3_, R_4_, R_5_ = OMe R_2_ = H	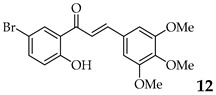	86
14		R_2_, R_4_, R_5_ = OMe	--	No reaction
15		R_3_, R_5_ = OMe R_2_, R_4_ = H	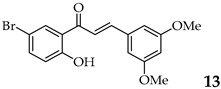	94

**Table 4:** Some green chemistry parameters.

Settings	In Solution	Ball Mill
Yield (%)	47	86
Reaction time (min)	120	60
Energy consumption (W × h)	38	30
E-factor	560	293

**Table 5:** Biological effects of the synthesized chalcones.

Compound	*L. donovani* Axenic Amastigote	*L. donovani* Intramacrophage Amastigote	SI * (Selectivity Index)	3D7 *P.* *falciparum*	IGR-39 Cells Viability (24 h)	IGR-39 Cells Viability (72 h)
	IC_50_ ± SD (µM)	IC_50_ ± SD (µM)		IC_50_ ± SD (µM)	IC_50_ (µM)	IC_50_ (µM)
**1**	17.37 ± 2.64	5.42 ± 1.48	10.7	>100	>>100	>>100
**2**	14.24 ± 2.96	12.80 ± 1.61	>7.81	7.59 ± 1.46	82.7	54.1
**3**	5.59 ± 1.12	5.18 ± 1.68	>19.3	14.12 ± 1.99	>100	83.4
**4**	6.67 ± 1.52	1.59 ± 0.09	39	15.01± 0.08	>>100	>>100
**5**	8.68 ± 0.67	ND	ND	>100	>>100	>>100
**6**	2.33 ± 0.53	1.36 ± 0.51	>73.5	5.26 ± 0.05	>>100	>>100
**7**	4.29 ± 0.82	2.48 ± 0.42	>40.3	8.05 ± 0.03	>>100	>>100
**8**	9.82 ± 2.47	9.82 ± 2.47	>10.2	10.21 ± 2.56	>100	70.5
**9**	8.59 ± 1.08	ND	ND	13.41 ± 1.54	>>100	>>100
**10**	5.87 ± 2.11	10.03 ± 2.14	>9.97	36.84 ± 2.54	>>100	>>100
**11**	2.82 ± 0.77	3.29 ± 1.99	>30.4	51.35 ± 2.54	>>100	>>100
**12**	>100	>100	ND	17.51 ± 2.18	91	24.7
**13**	11.52 ± 0.47	15.22 ± 1.43	>6.57	15.11 ± 1.29	>100	51.5
**14**	11.38 ± 2.52	22.91 ± 3.91	>4.36	>100	>>100	>>100
**15**	9.44 ± 2.20	2.87 ± 0.36	>34.8	3.21	>>100	>>100
**16**	11.23 ± 2.06	13.49 ± 2.99	>7.41	53.43 ± 4.16	>>100	>>100
**17**	24.24 ± 2.56	18.41 ± 2.33	2.62	6.13 ± 0.51	66.3	38.6
Reference	3.66 ± 0.73 ^a^	5.78 ± 1.02 ^a^	9 ^a^	0.012 ± 0.002 ^b^	372.0 ^c^	25.0 ^c^

* Selectivity index (SI) corresponds to CC_50_/IC_50_, CC50 being defined for macrophage RAW 264.7 and IC_50_ being defined for the intramacrophage form of *L. donovani*; ^a^ Miltefosine (also called hexadecylphosphorylcholine as HePC), ^b^ Mefloquine, ^c^ Dacarbazine.

## Data Availability

All the data supporting reported results are presented in the manuscript and Supplementary Materials.

## Supplementary Materials

The following supporting information can be downloaded at https://www.mdpi.com/article/10.3390/molecules29081819/s1. All recorded NMR (in CDCl_3_), MS, and HRMS spectra in Figures S1–S72.
