# Supplementary material for: The Synthesis of 2′-Hydroxychalcones under Ball Mill Conditions and Their Biological Activities
Source: Molecules. 2024 Apr 17;29(8):1819. doi: 10.3390/molecules29081819 (PMC11054009; doi:10.3390/molecules29081819)
Supplement: Supplementary file 1 [file molecules-29-01819-s001.zip › molecules-2793299-supplementary.pdf]

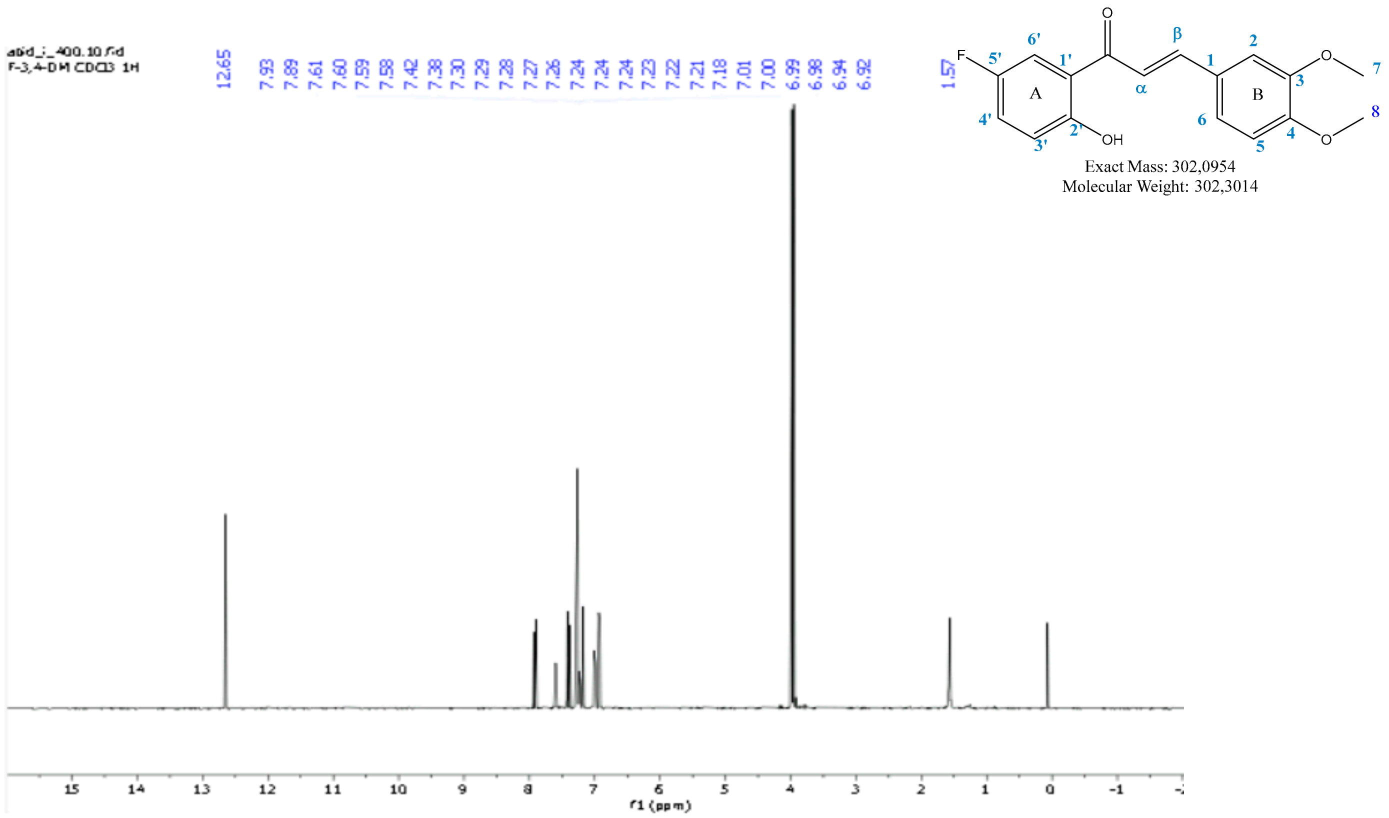

Figure S1 : ^{1}H NMR of compound (1)

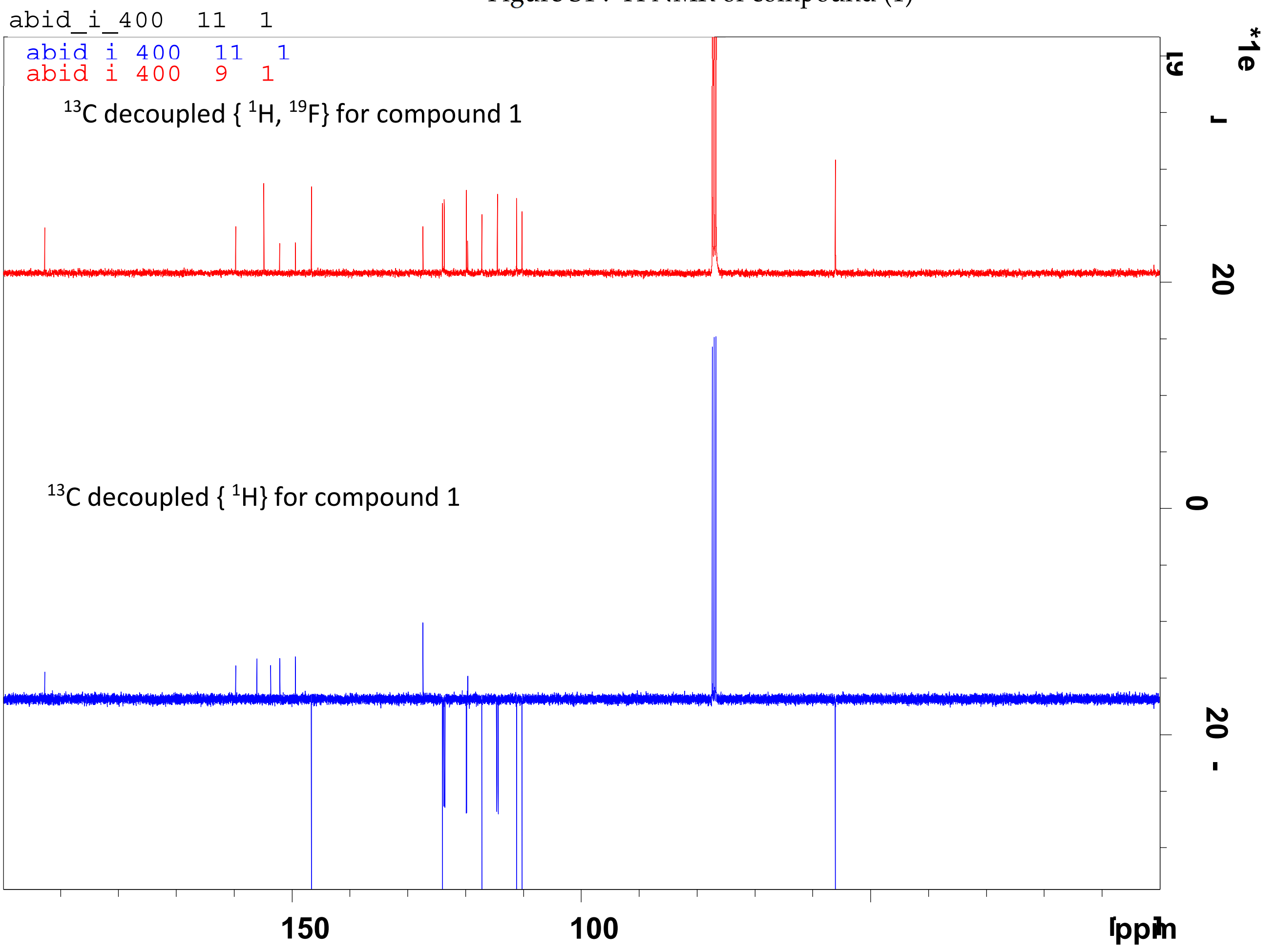

Figure S2 : ^{13}C NMR of compound (1)

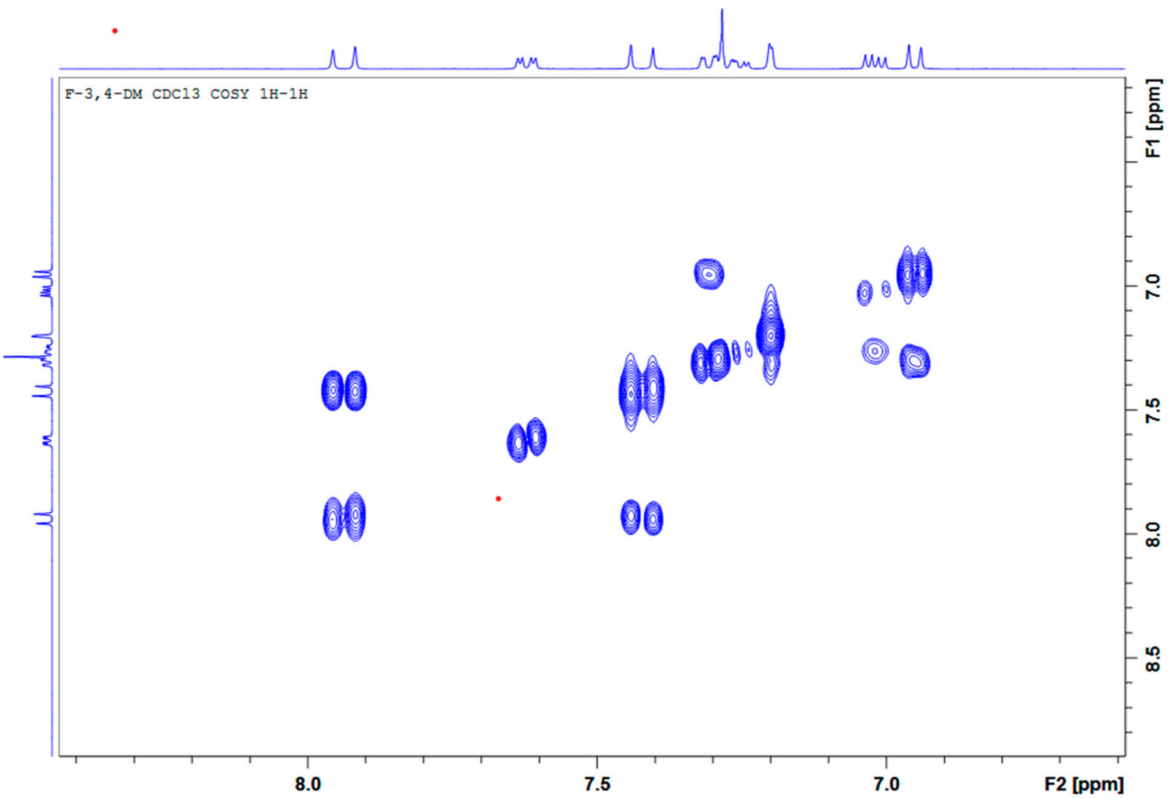

Figure S3 : NMR 2D cosy of compound (1)

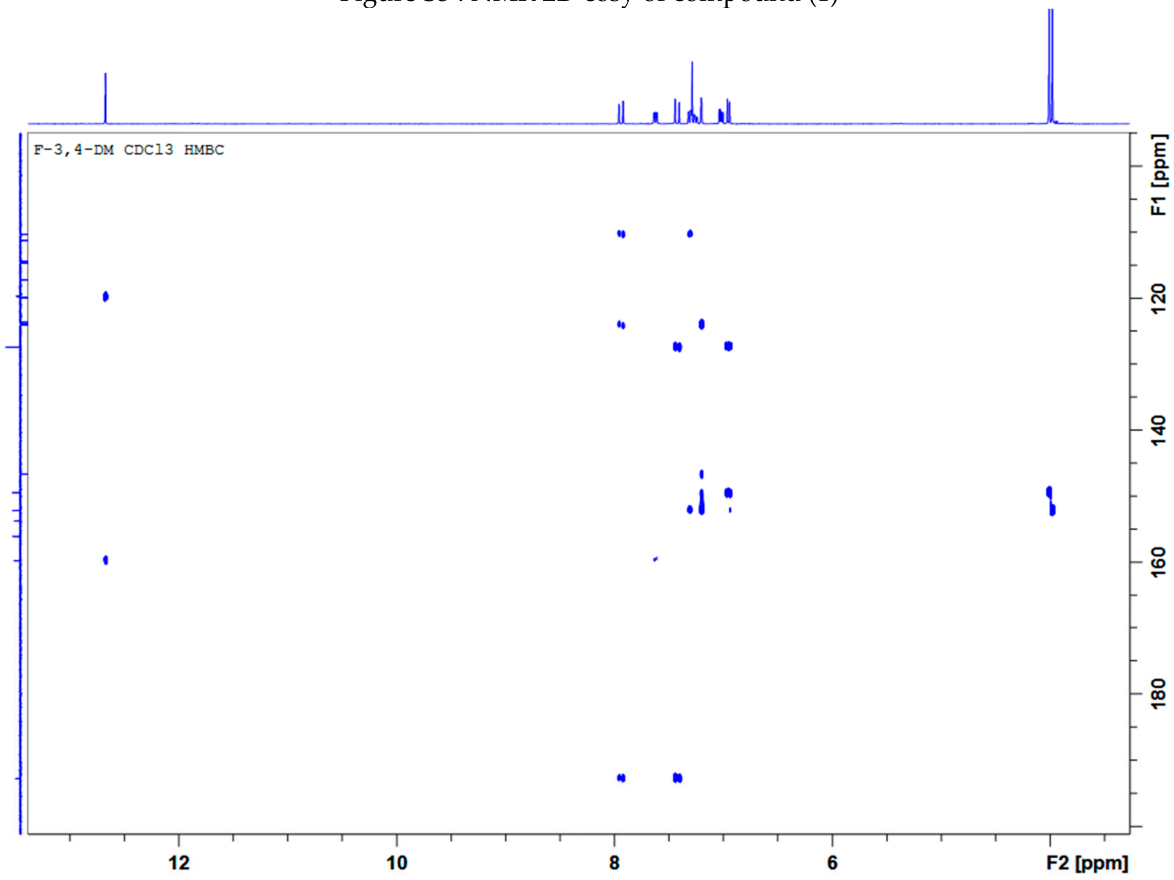

Figure S4 : NMR 2D HMBC of compound (1)

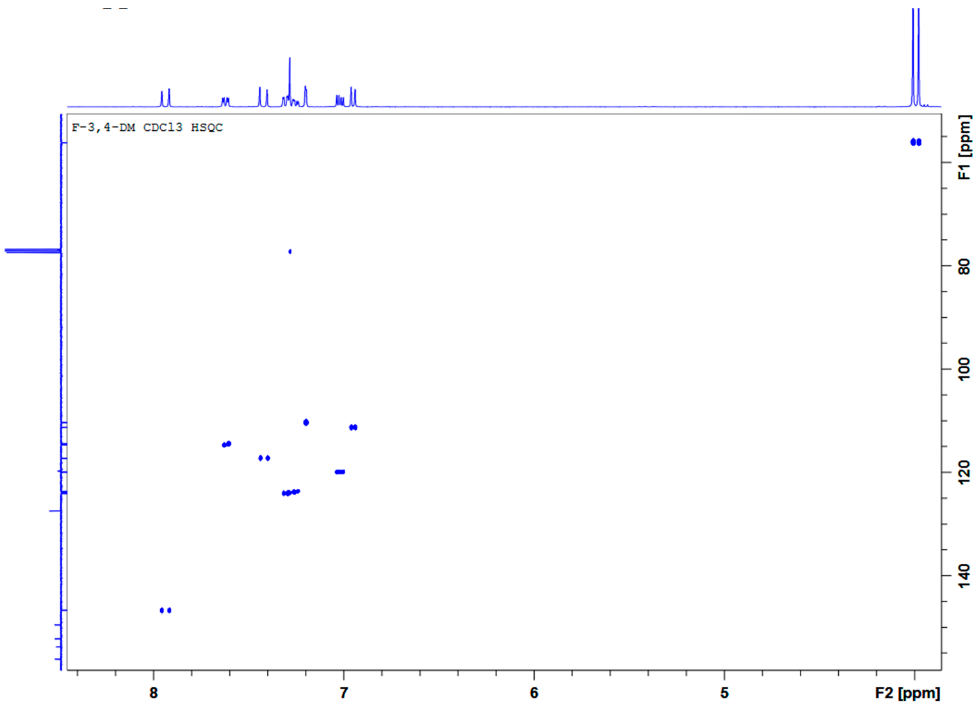

Figure S5 : NMR 2D HSQC of compound (1)

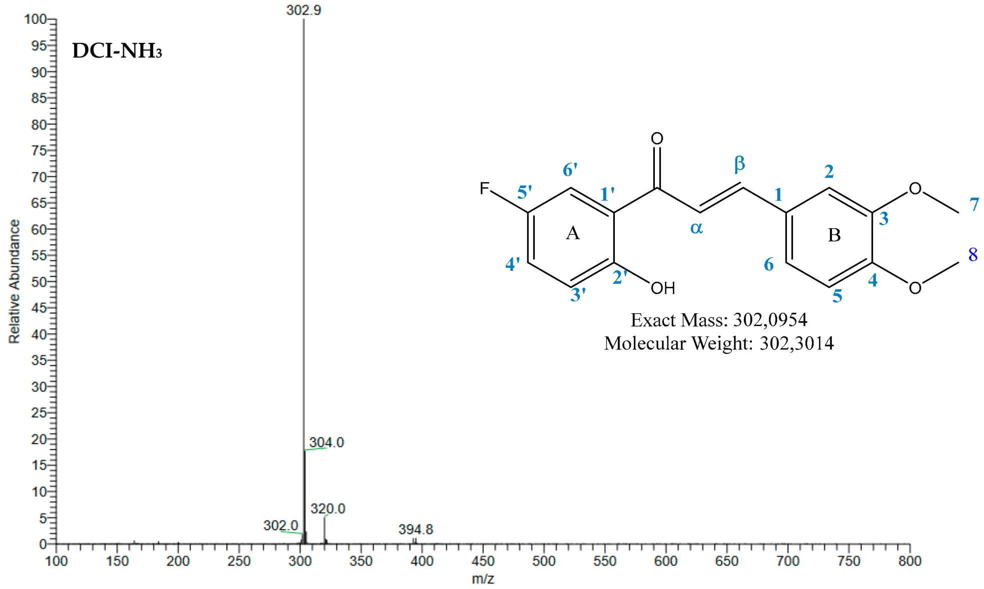

Figure S6 : MS of compound (1)

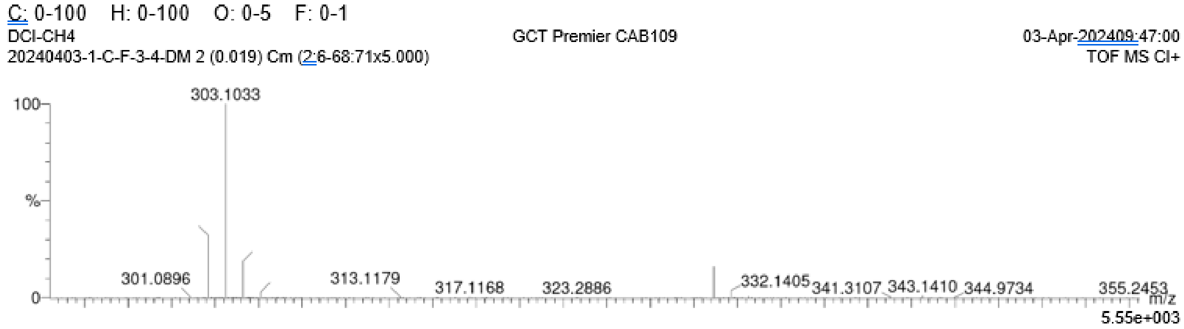

Figure S7 : HRMS of compound (1)

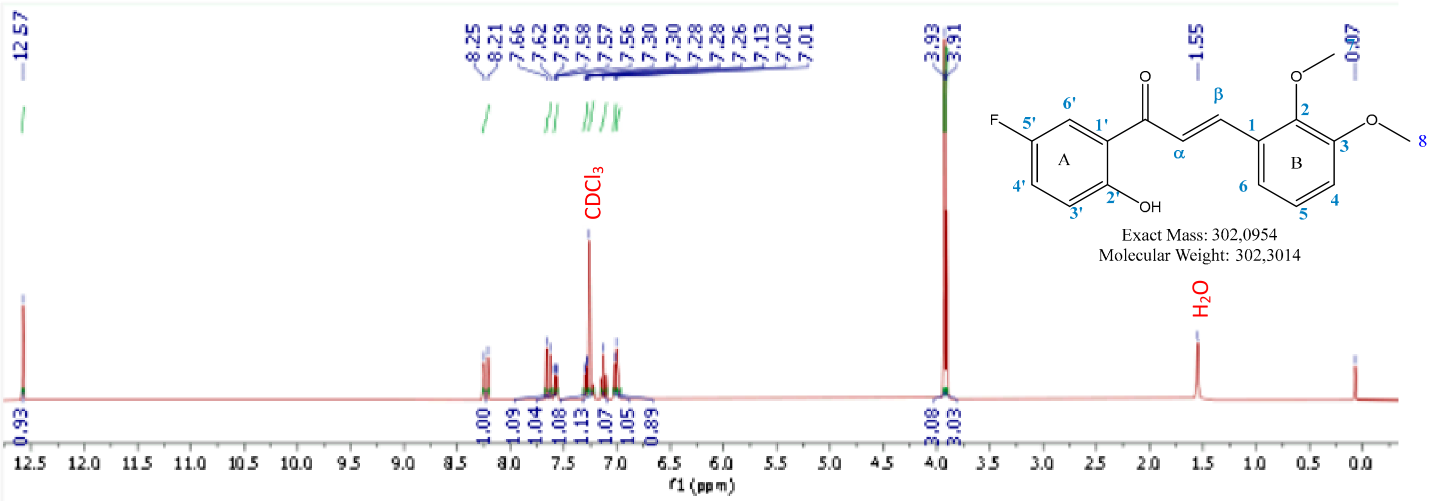

Figure S8 : ^{1}H NMR of compound (2)

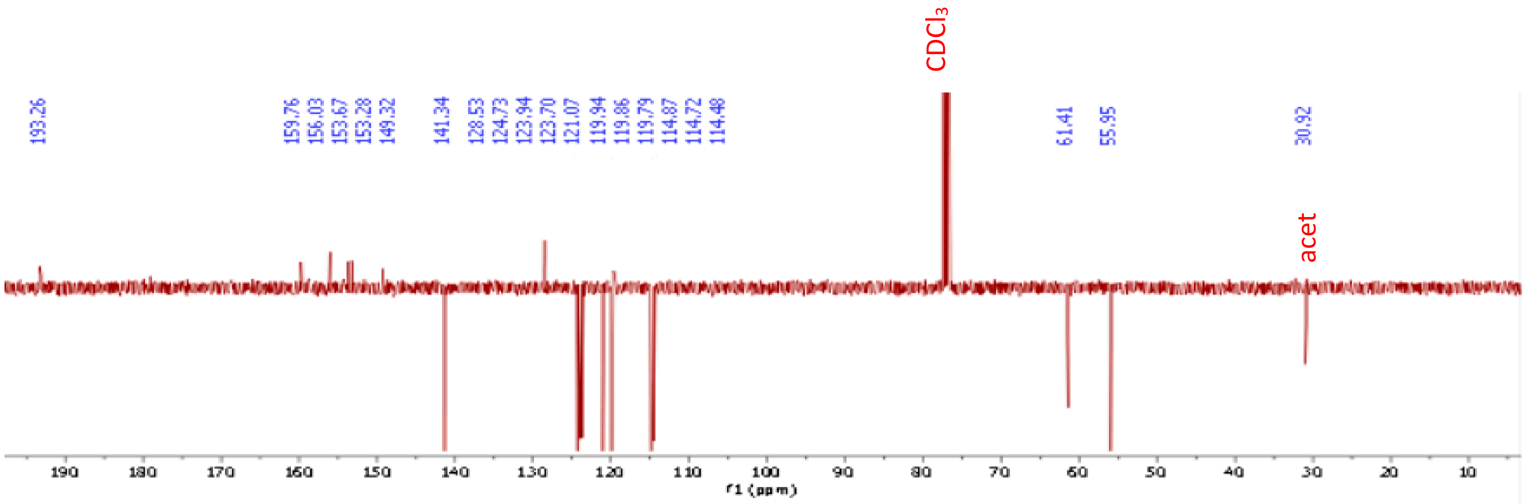

Figure S9 : ^{13}C NMR of compound (2)

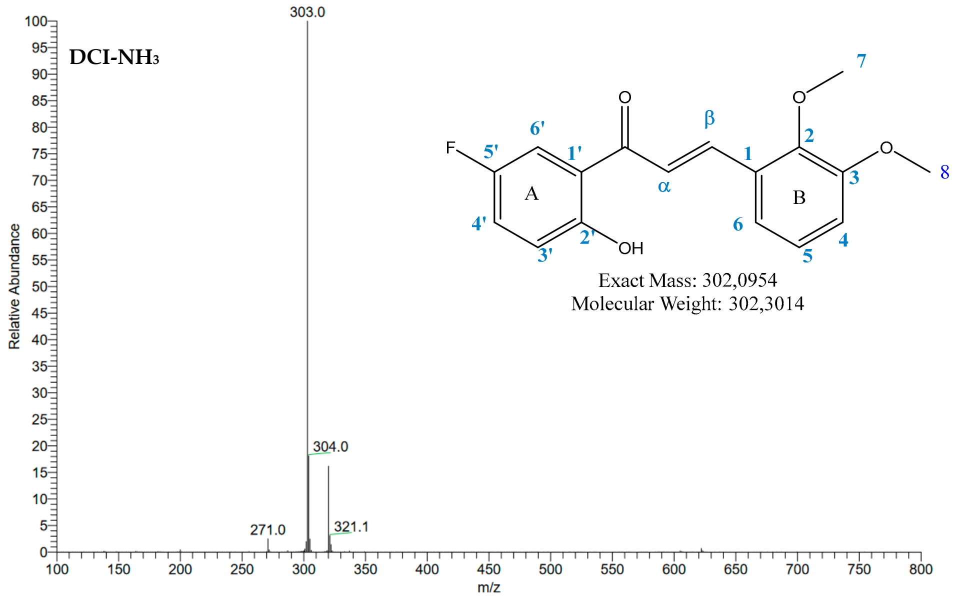

Figure S10 : MS of compound (2)

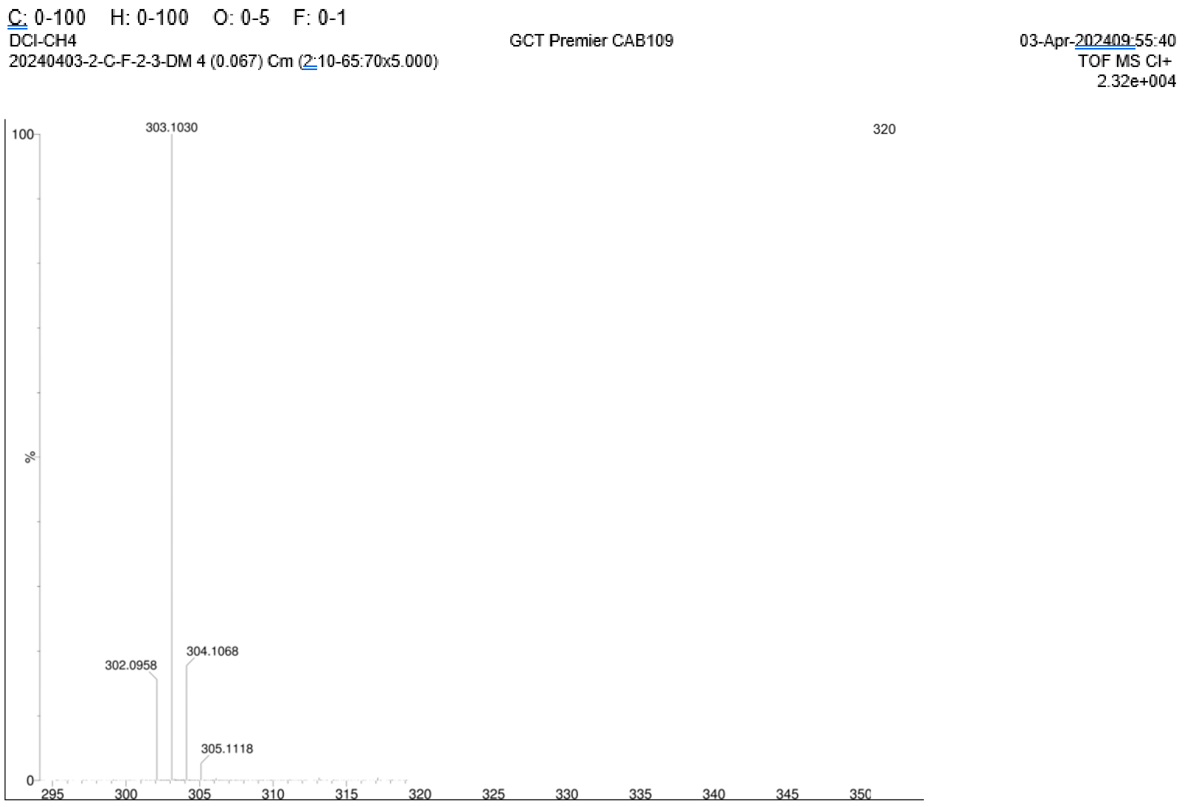

Figure S11 : HRMS of compound (2)

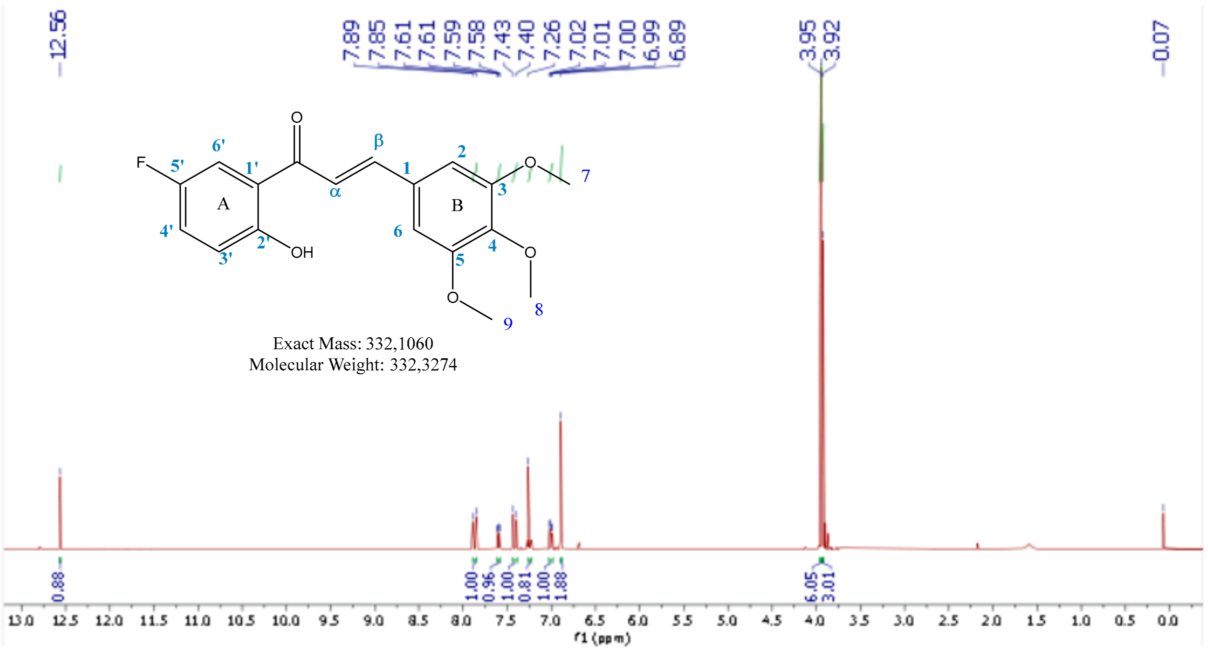

Figure S12 : ^{1}H NMR of compound (3)

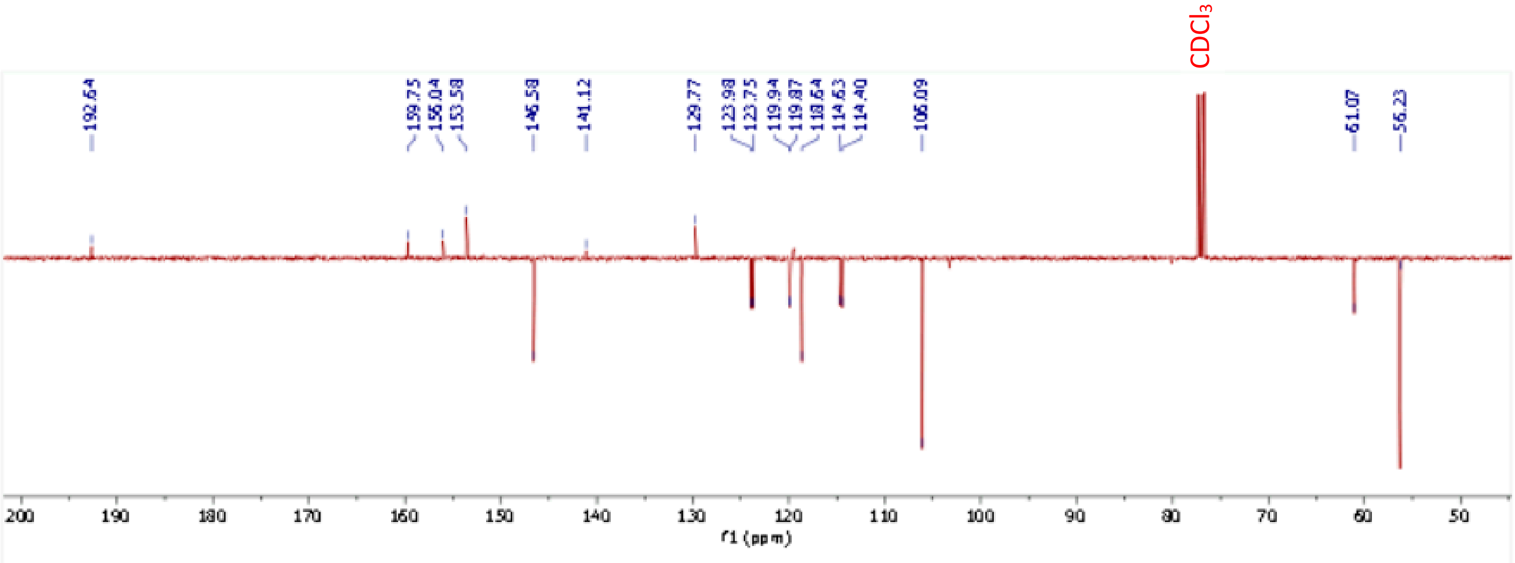

Figure 13 : ^{13}C NMR of compound (3)

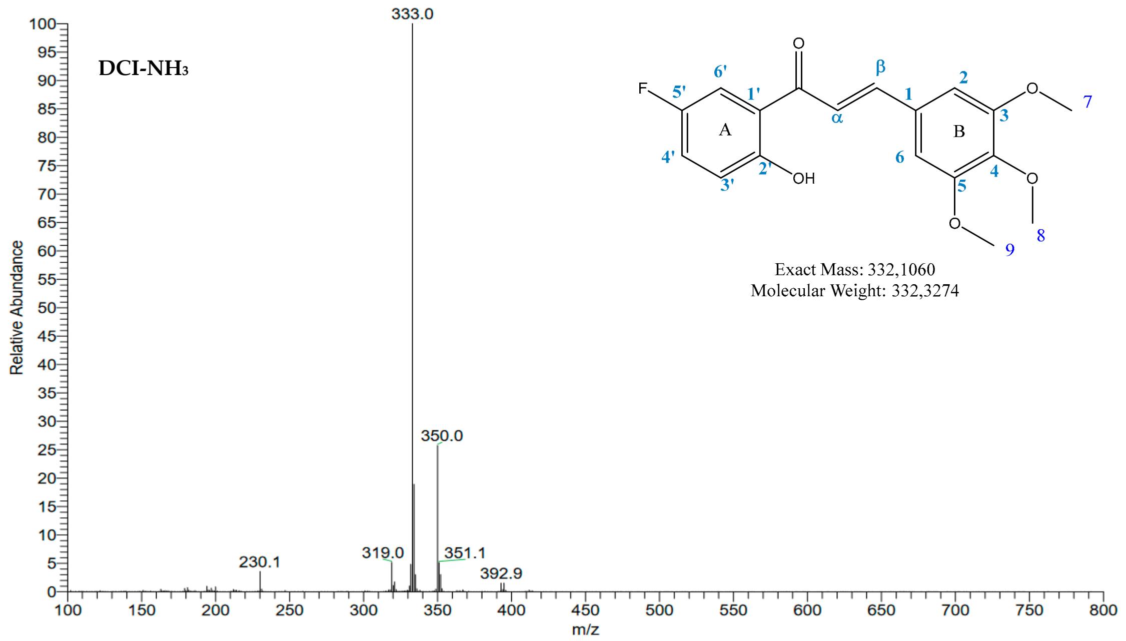

Figure S14 : MS of compound (3)

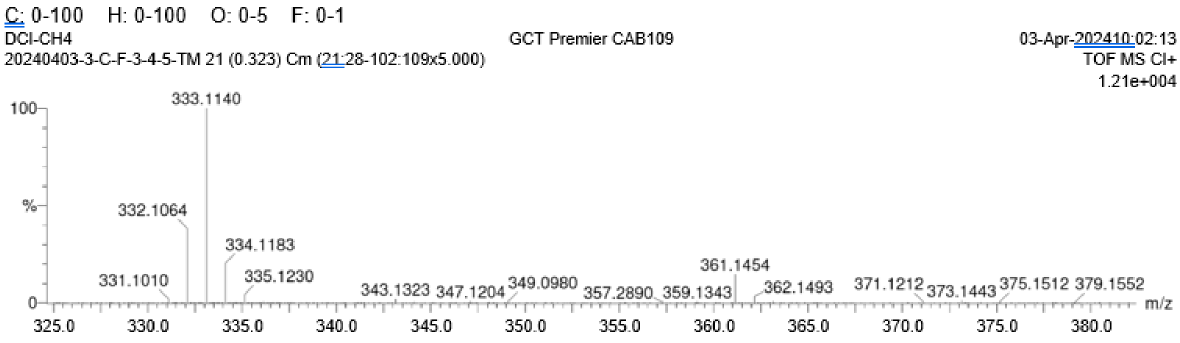

Figure S15 : HRMS of compound (3)

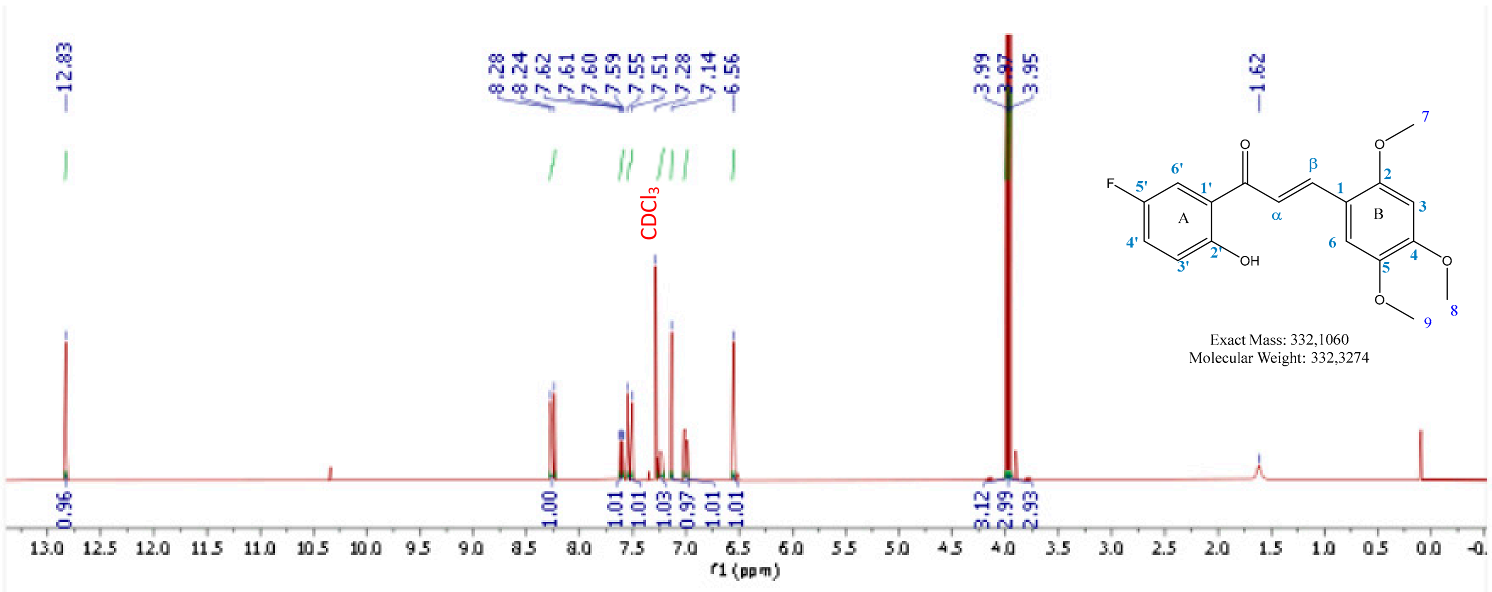

Figure S16 : ^{1}H NMR of compound (4)

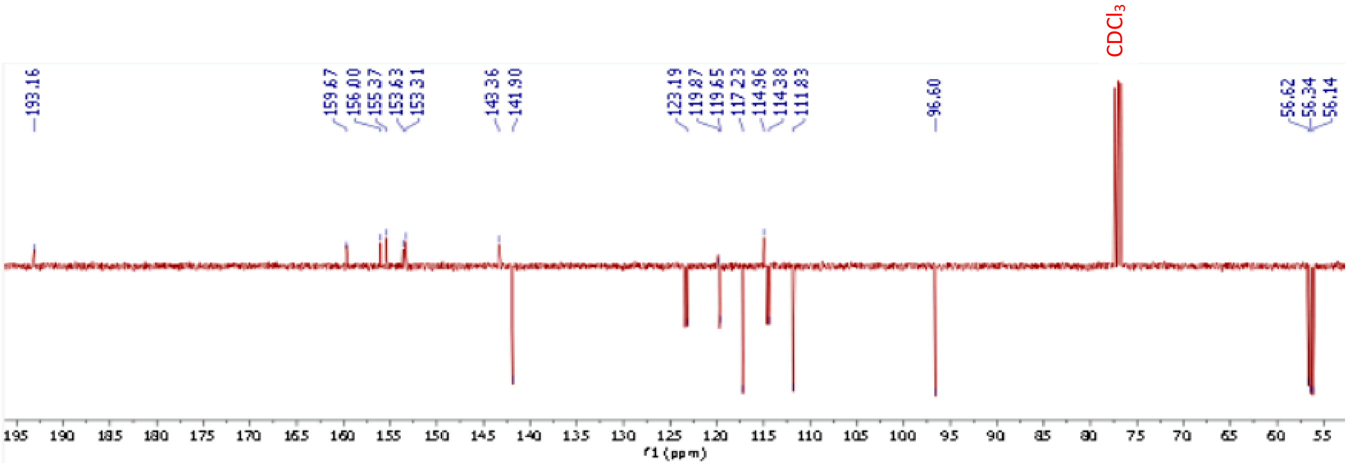

Figure S17 : ^{13}C NMR of compound (4)

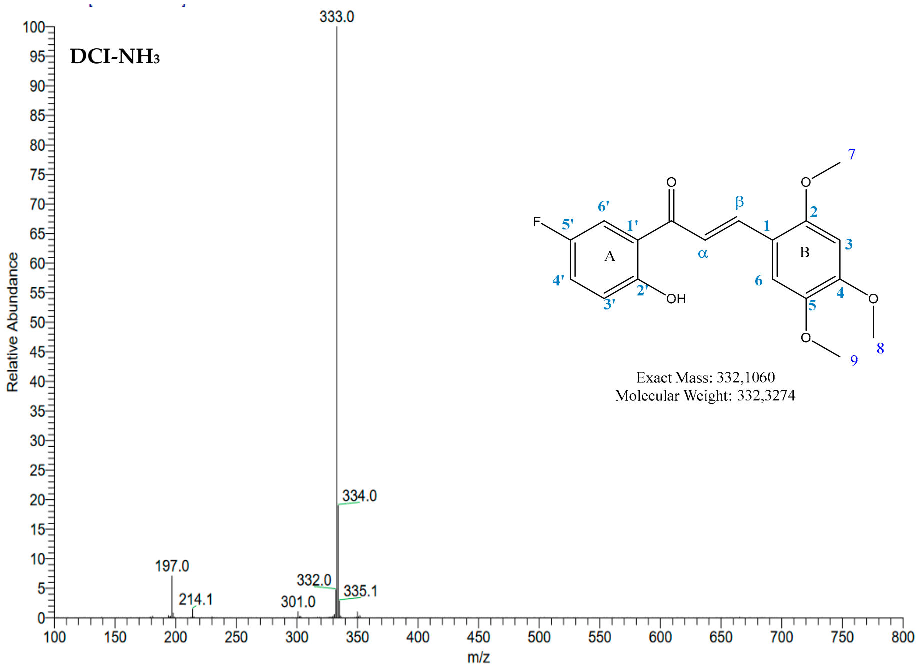

Figure S18 : MS of compound (4)

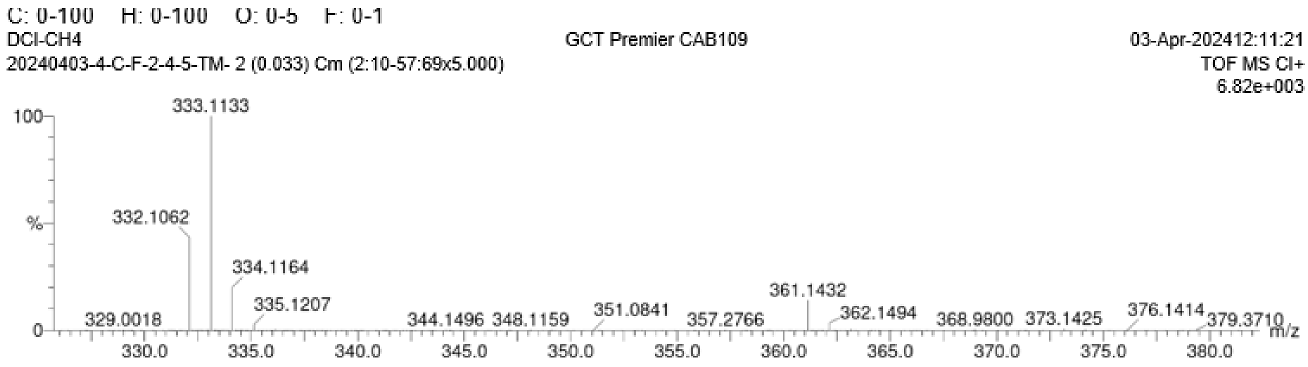

Figure S19 : HRMS of compound (4)

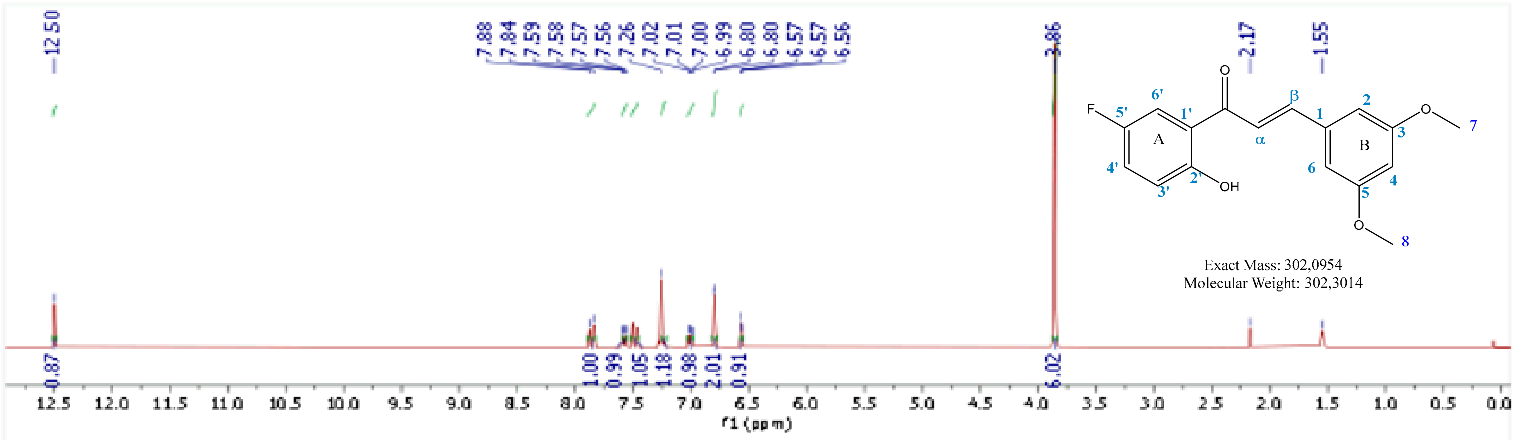

Figure S20 : ^{1}H NMR of compound (5)

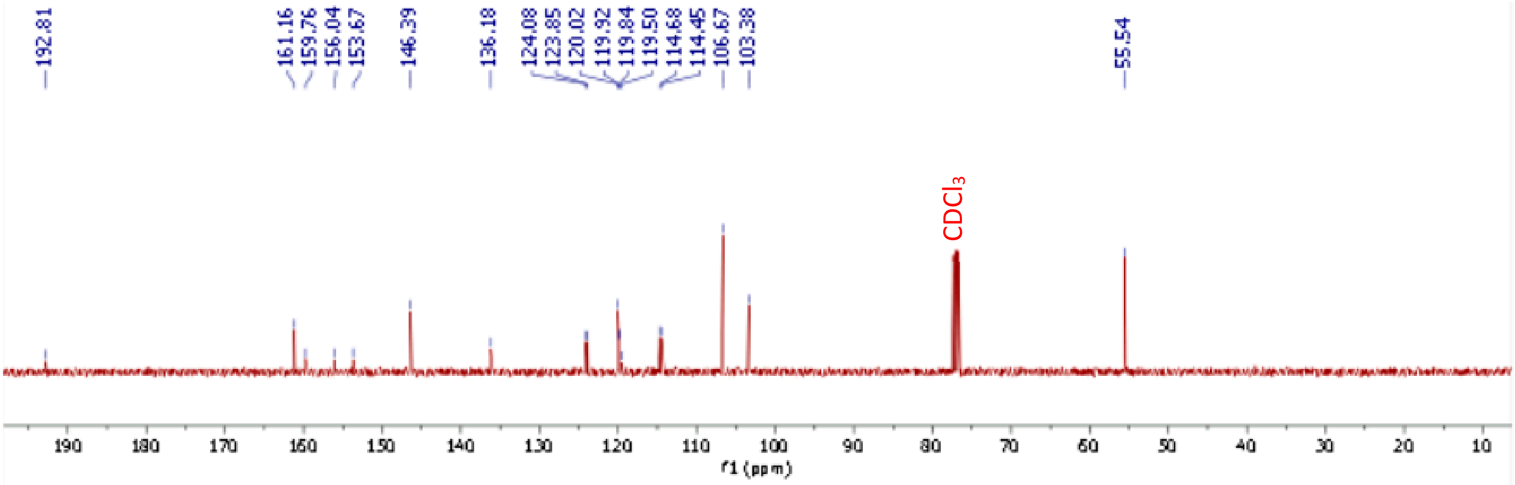

Figure S21 : ^{13}C NMR of compound (5)

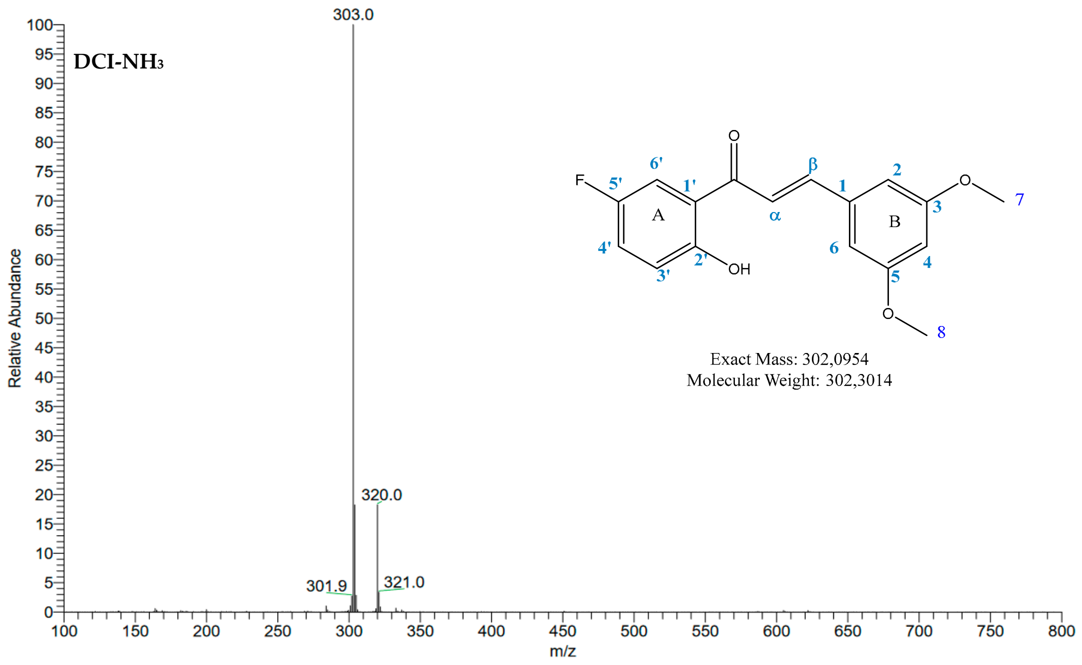

Figure S22 : MS of compound (5)

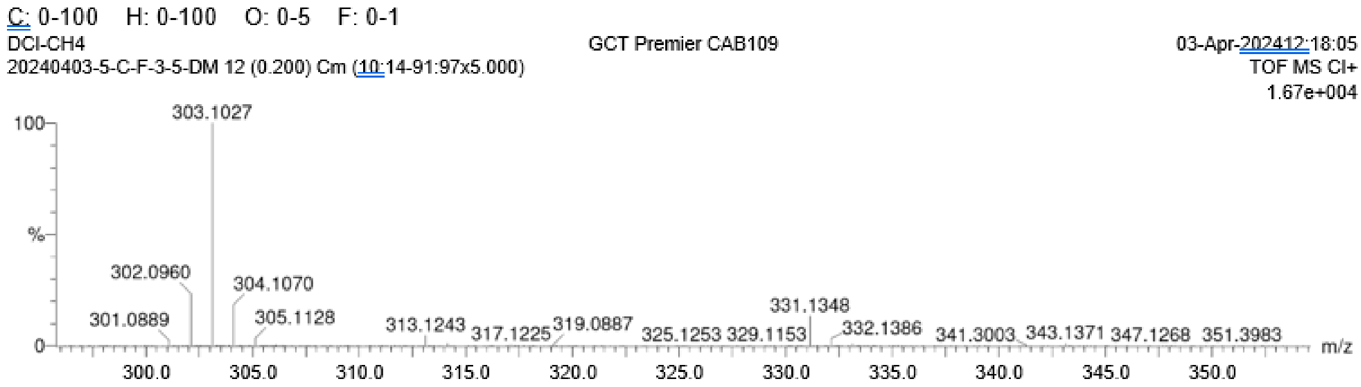

Figure S23 : HRMS of compound (5)

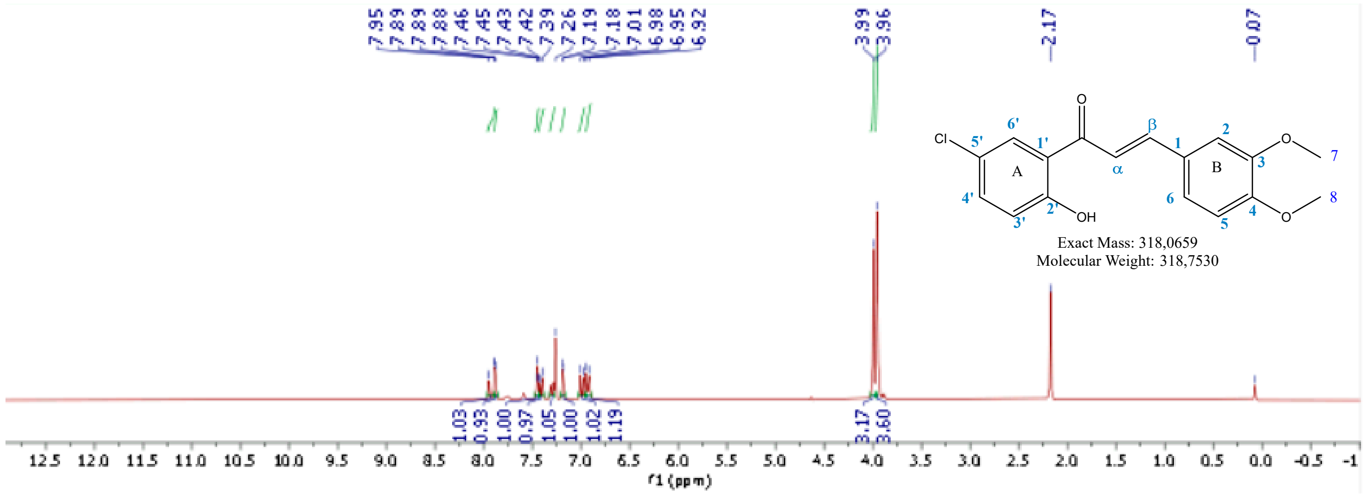

Figure S24 : ^{1}H NMR of compound (6)

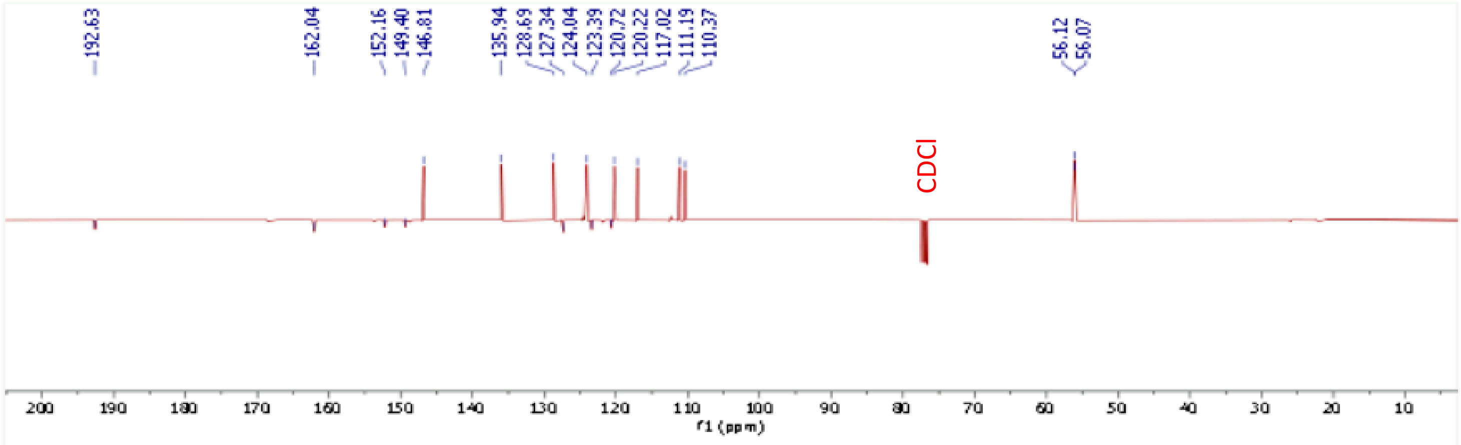

Figure S25 : ^{13}C NMR of compound (6)

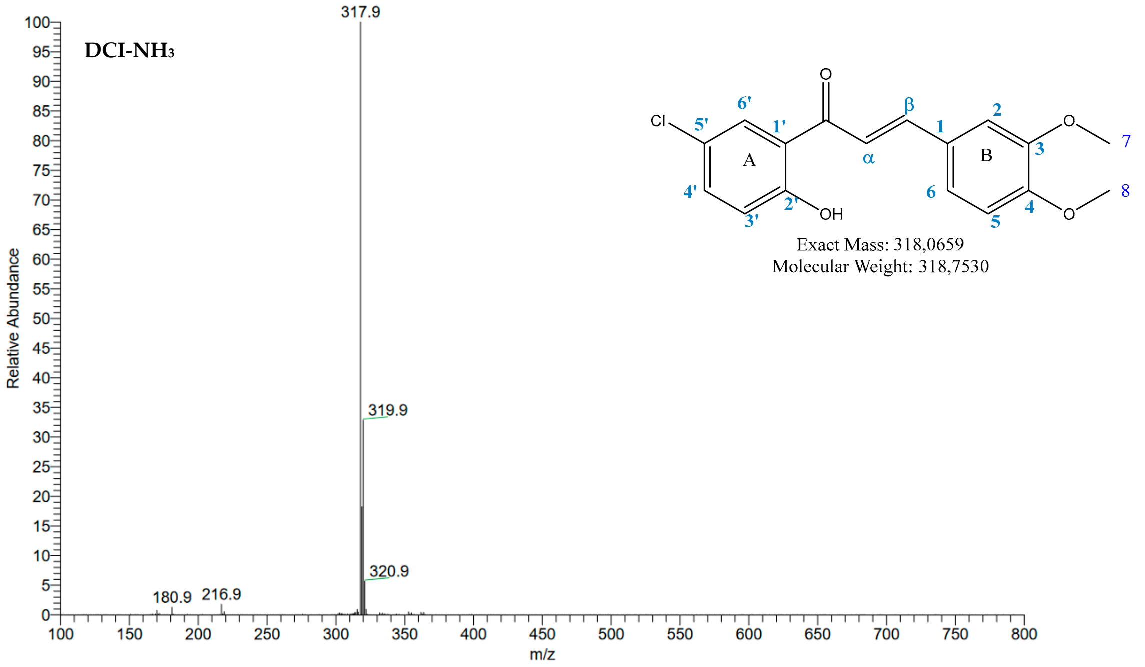

Figure S26 : MS of compound (6)

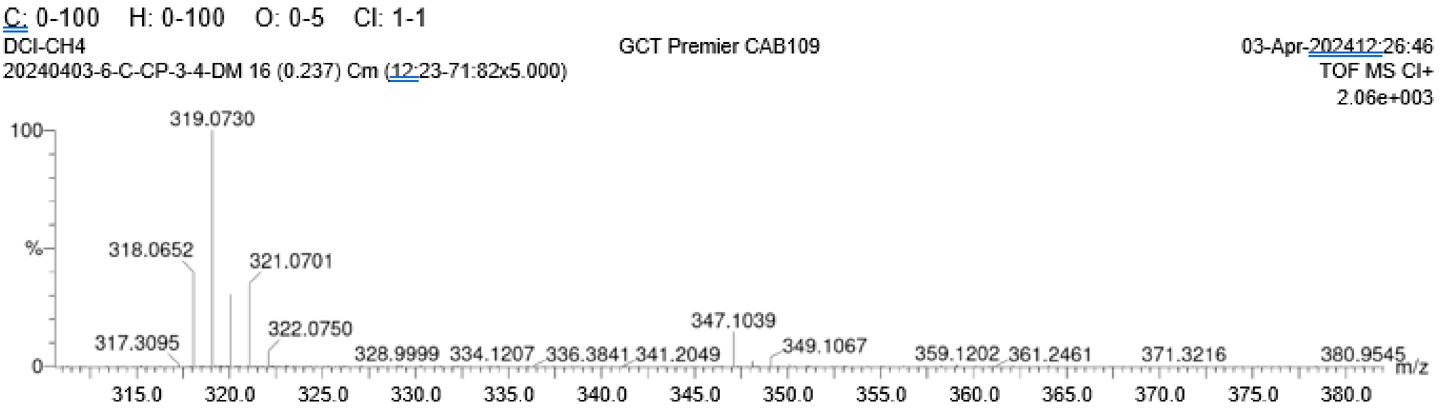

Figure S27 : HRMS of compound (6)

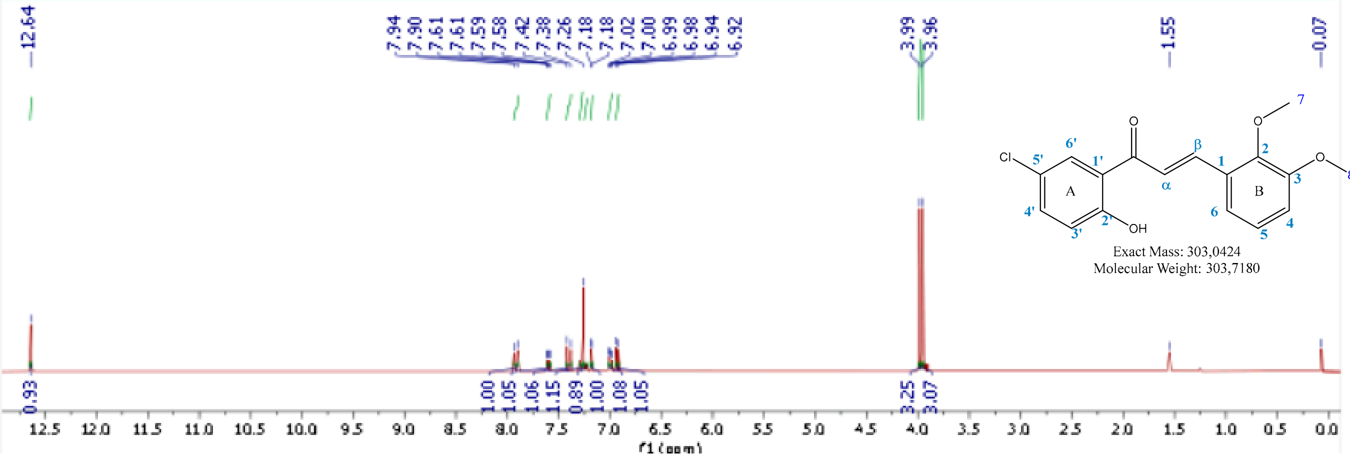

Figure S28 : ^{1}H NMR of compound (7)

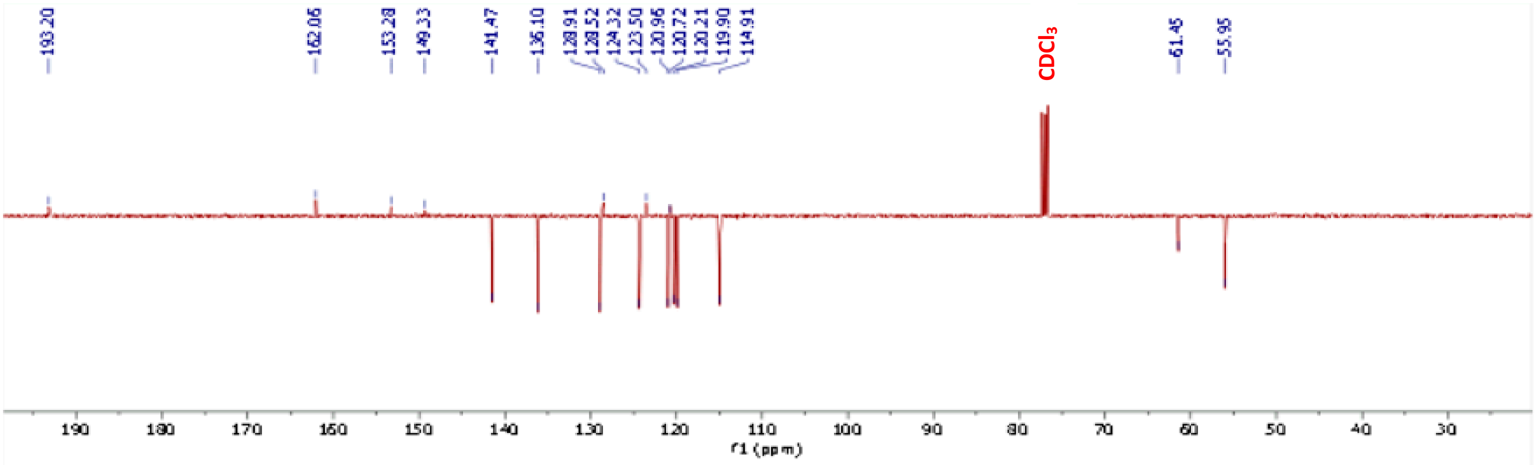

Figure S29 : ^{13}C NMR of compound (7)

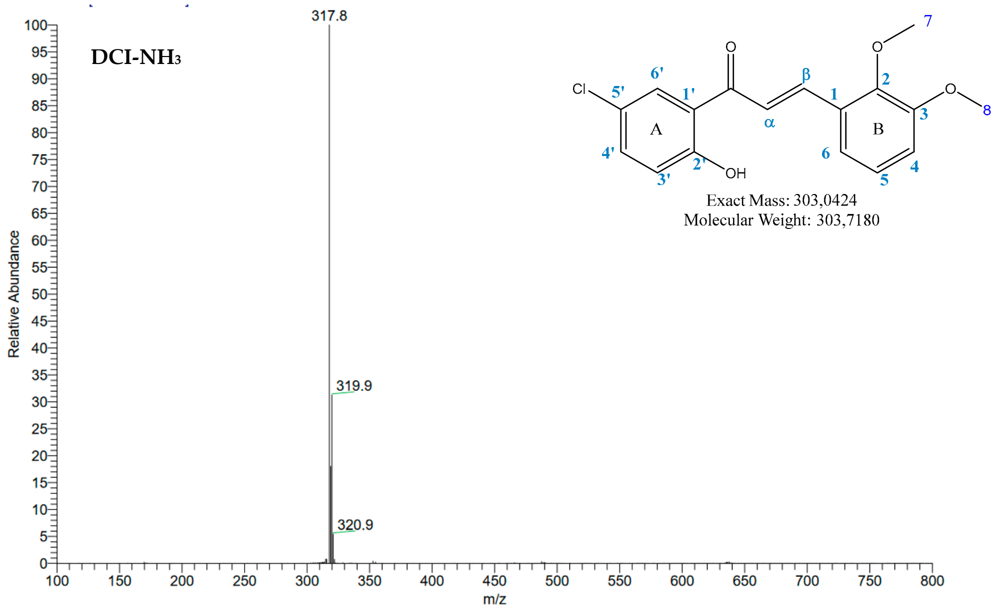

Figure S30 : MS of compound (7)

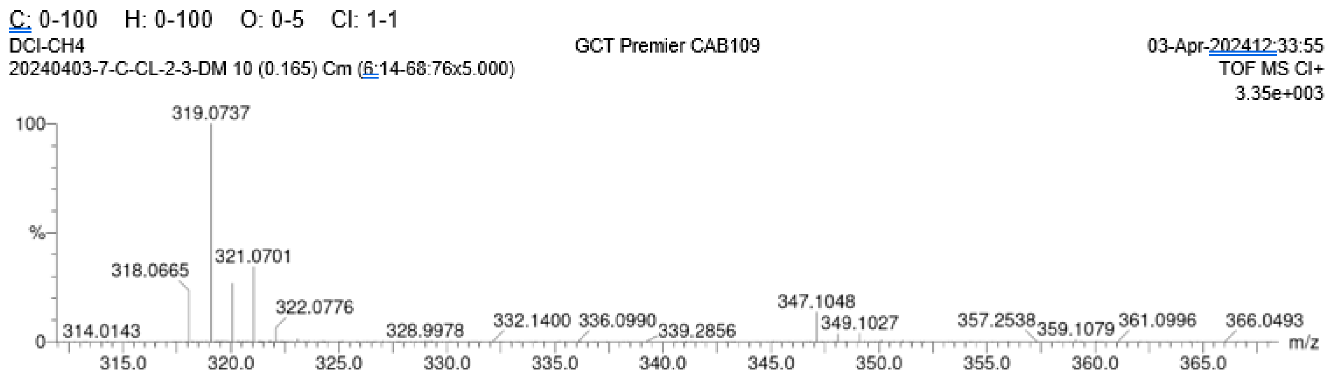

Figure S31 : HRMS of compound (7)

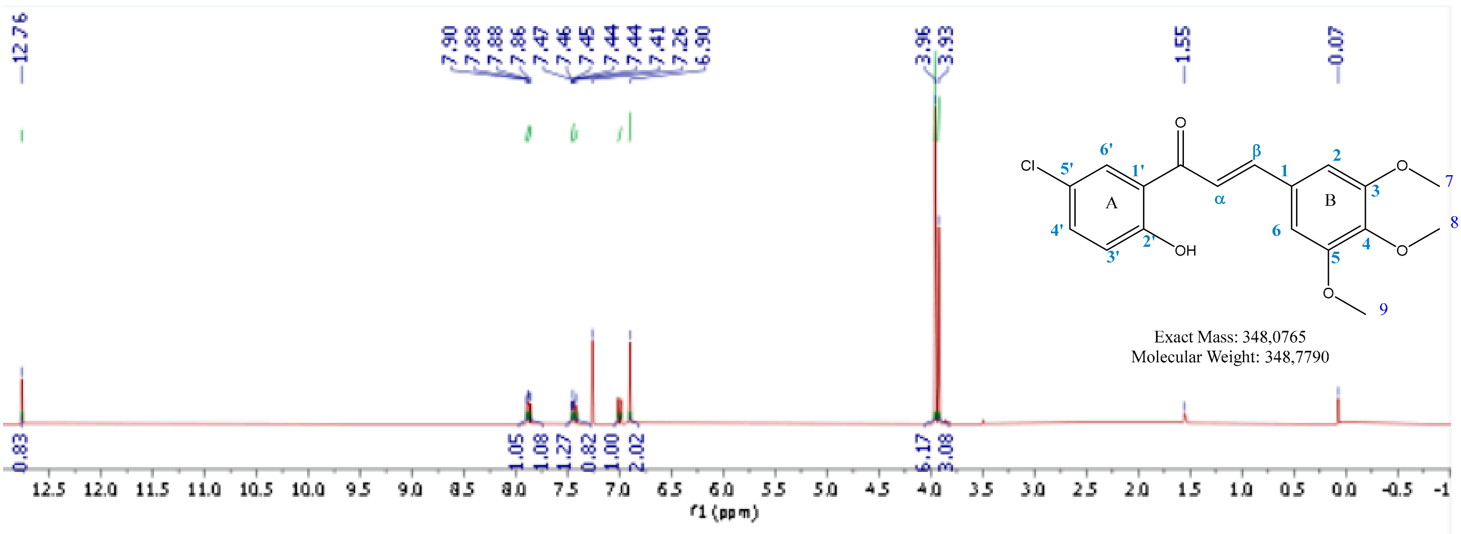

Figure S32 : ^{1}H NMR of compound (8)

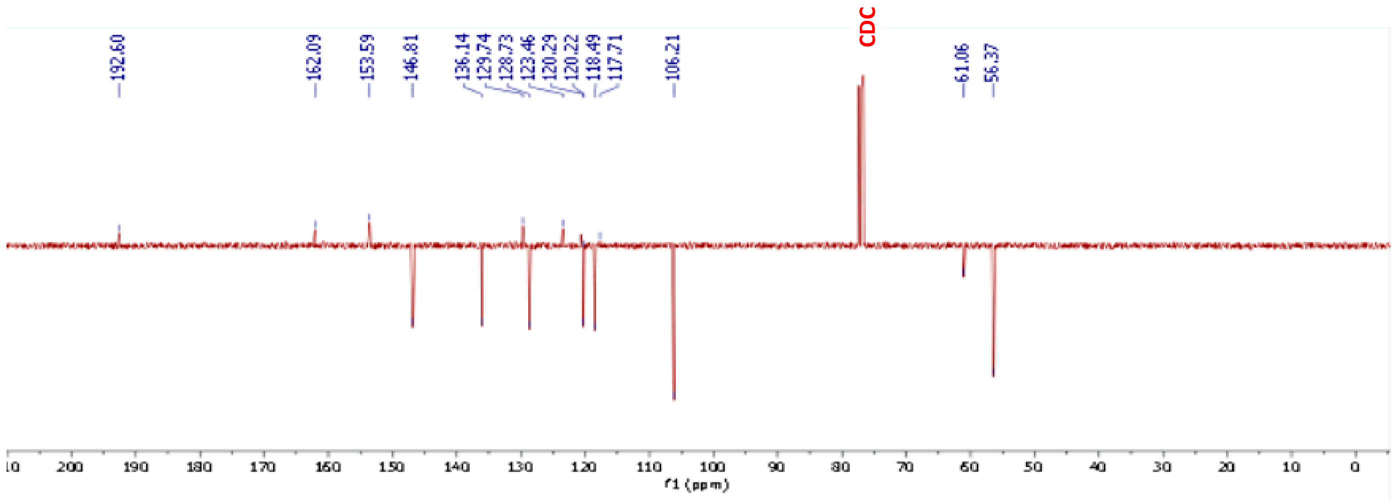

Figure S33 : ^{13}C NMR of compound (8)

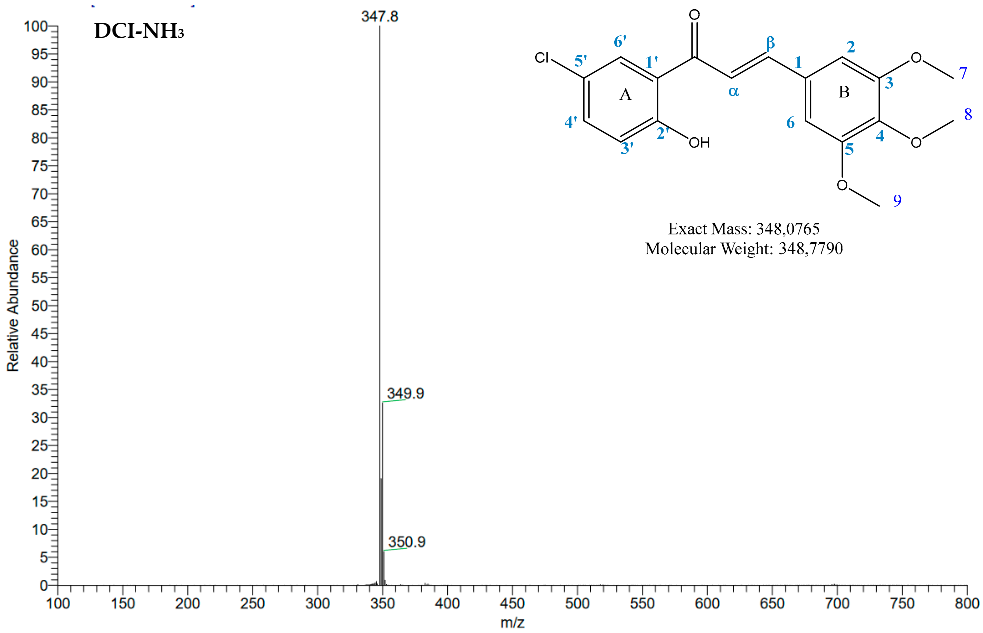

Figure S34 : MS of compound (8)

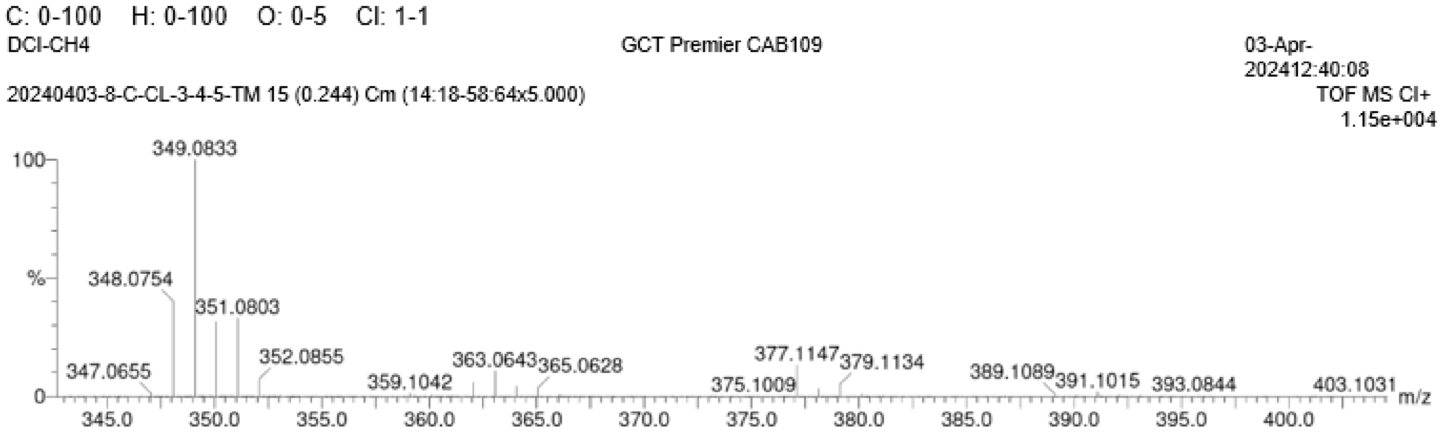

Figure S35 : HRMS of compound (8)

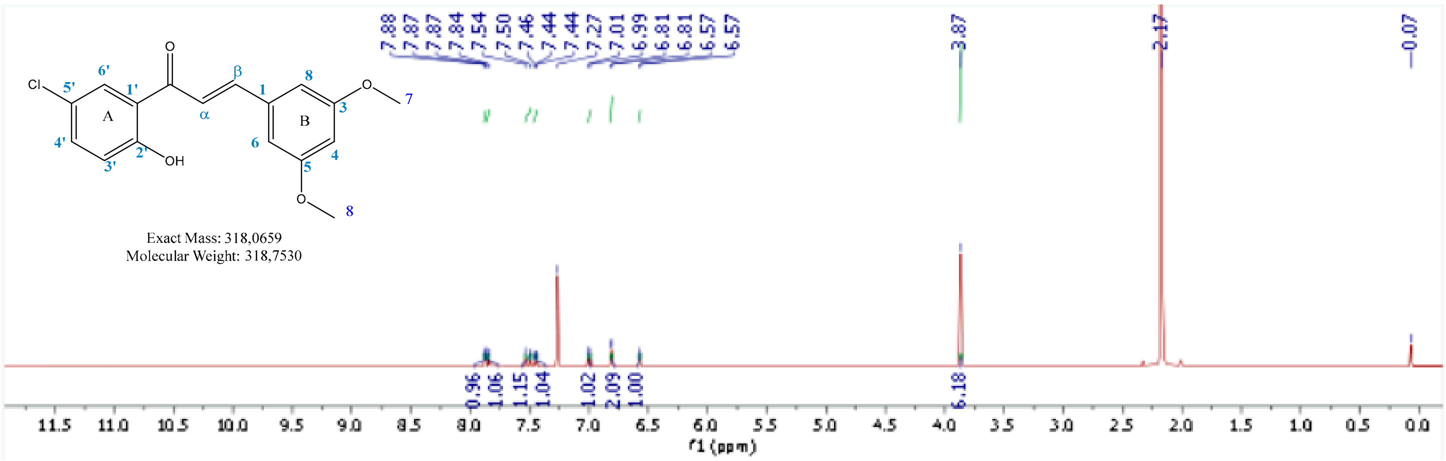

Figure S36 : ^{1}H NMR of compound (9)

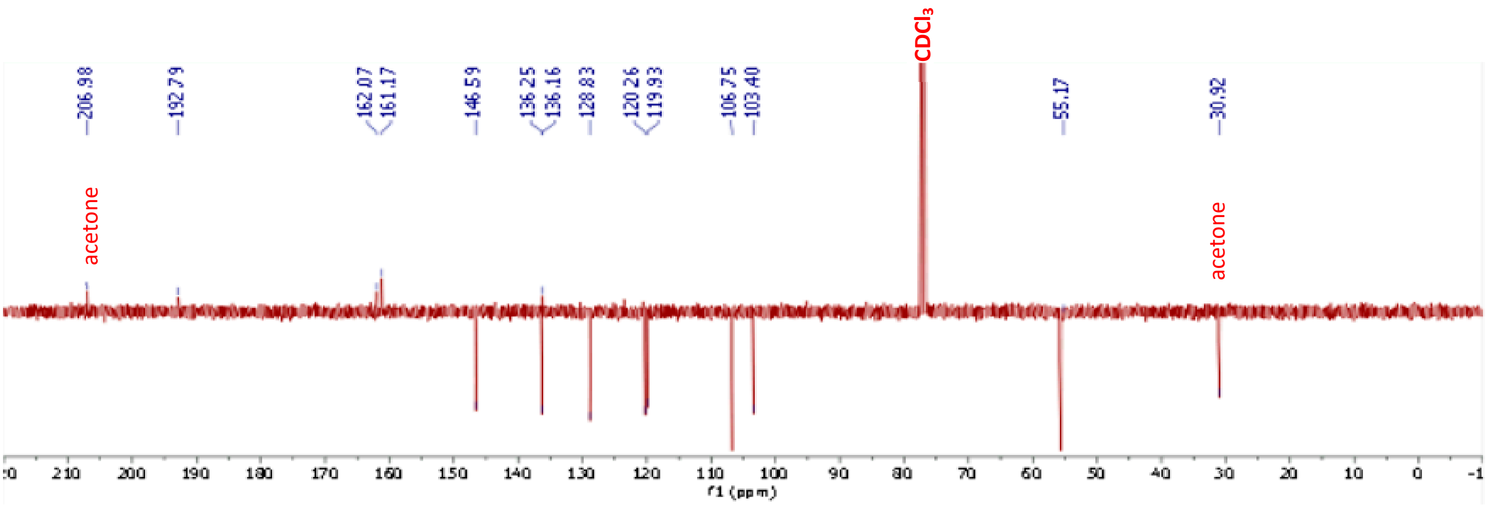

Figure S37 : ^{13}C NMR of compound (9)

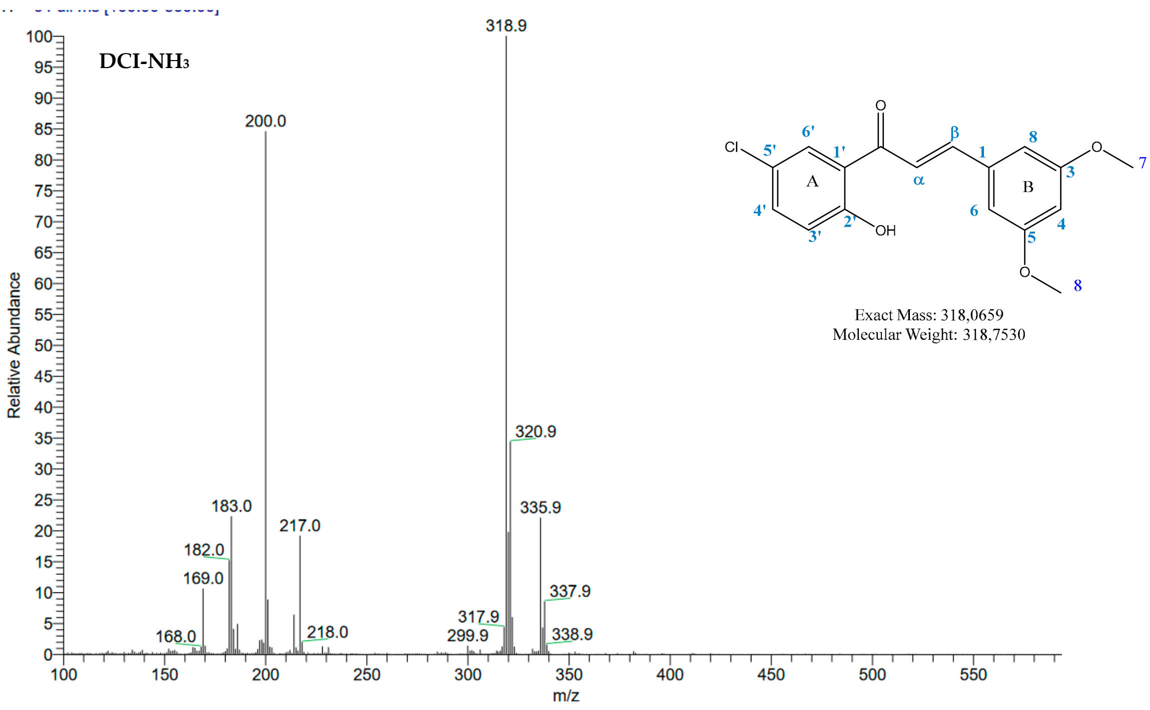

Figure S38 : MS of compound (9)

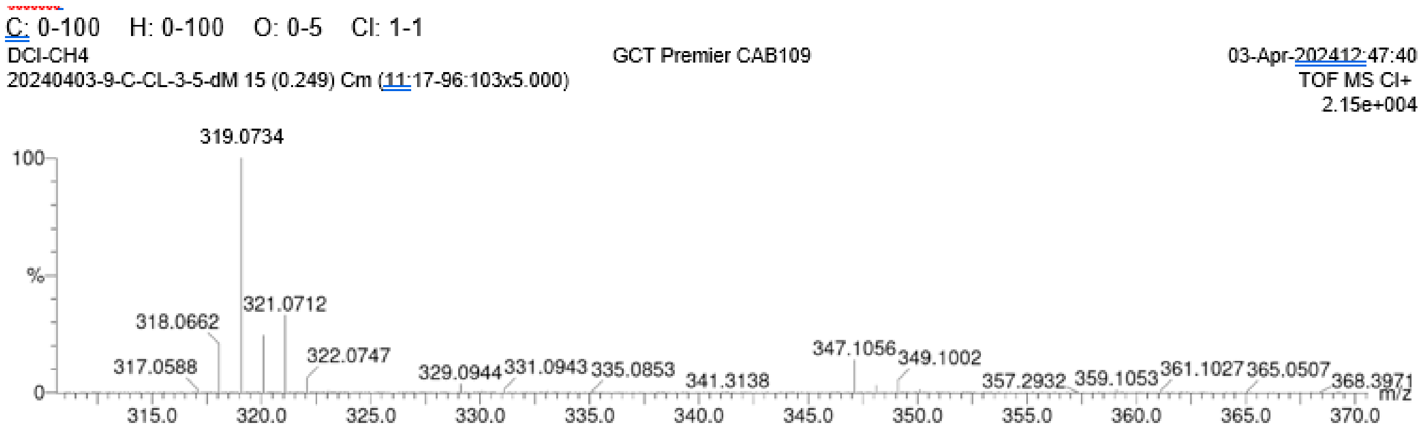

Figure S39 : HRMS of compound (9)

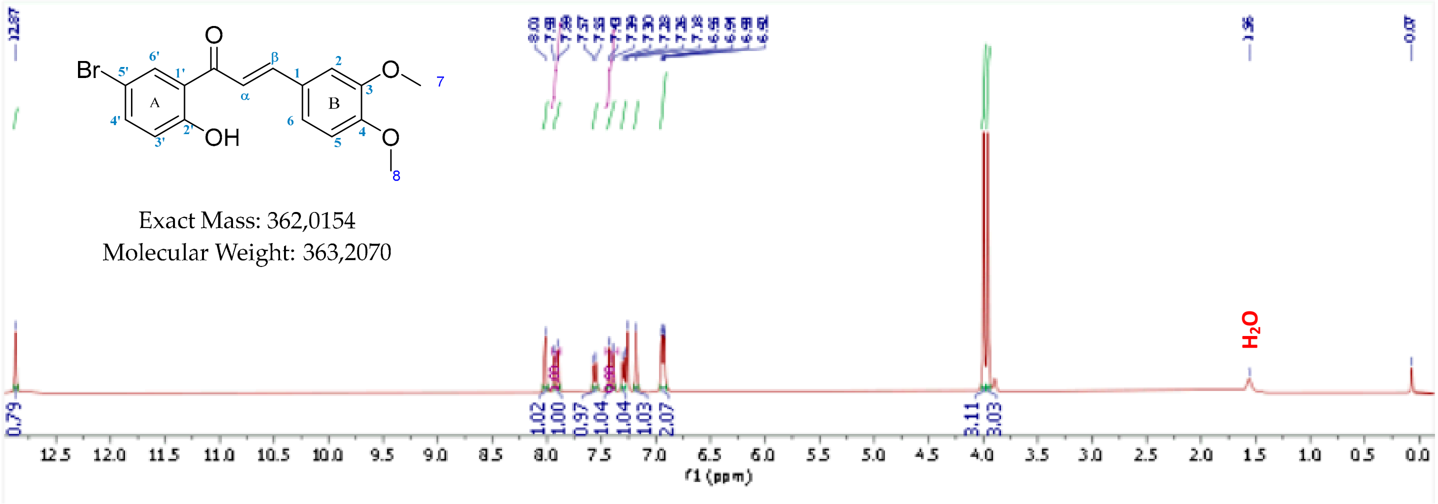

Figure S40 : ^{1}H NMR of compound (10)

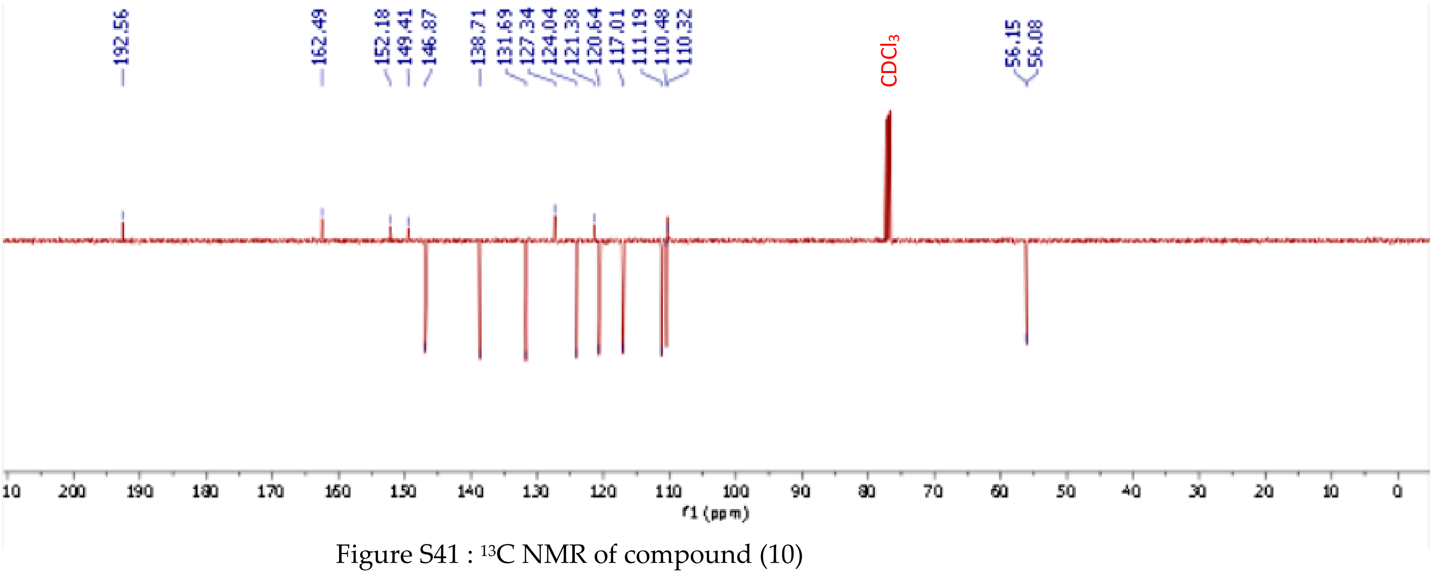

Figure S41 : ^{13}C NMR of compound (10)

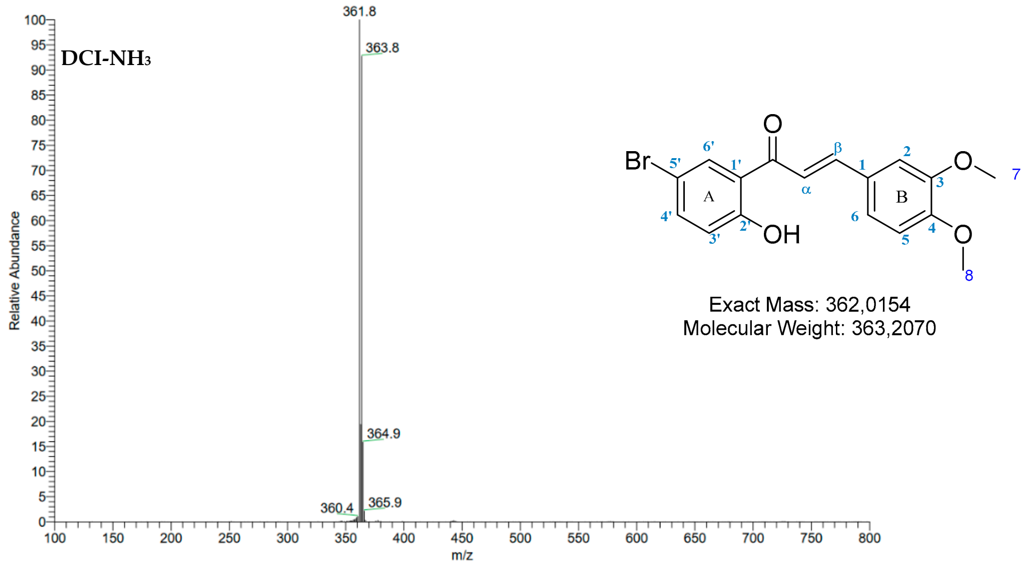

Figure S42 : MS of compound (10)

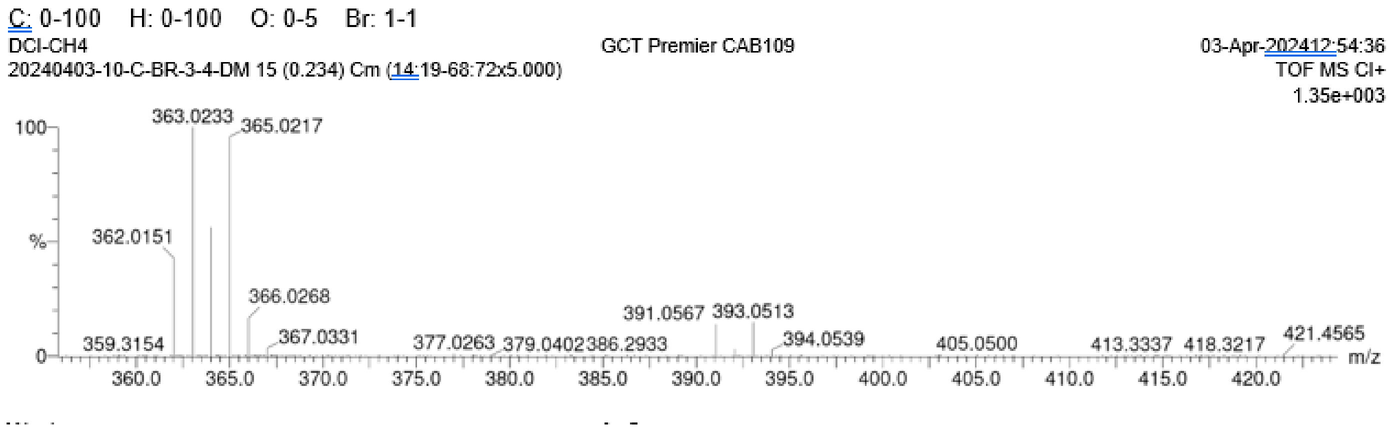

Figure S43 : HRMS of compound (10)

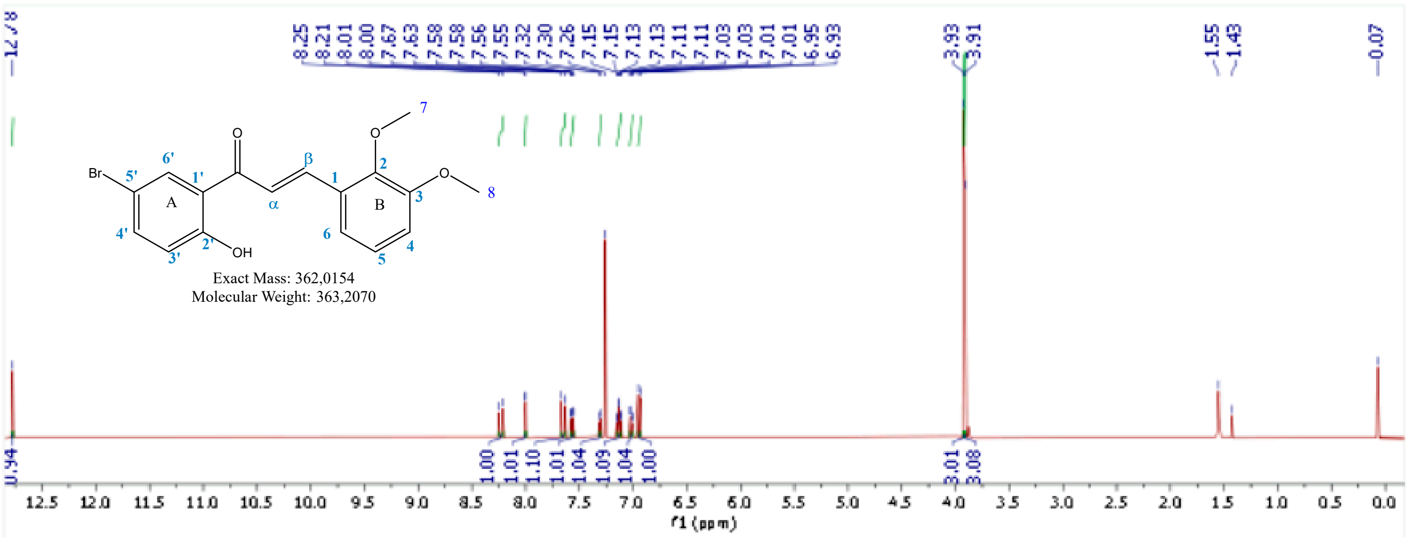

Figure S44 : ^{1}H NMR of compound (11)

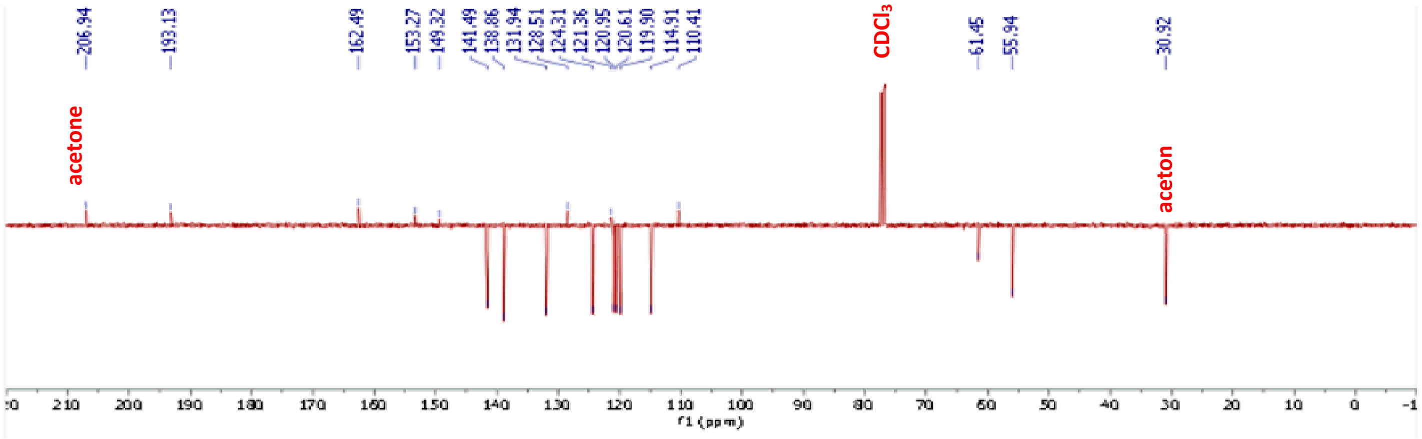

Figure S45 : ^{13}C NMR of compound (11)

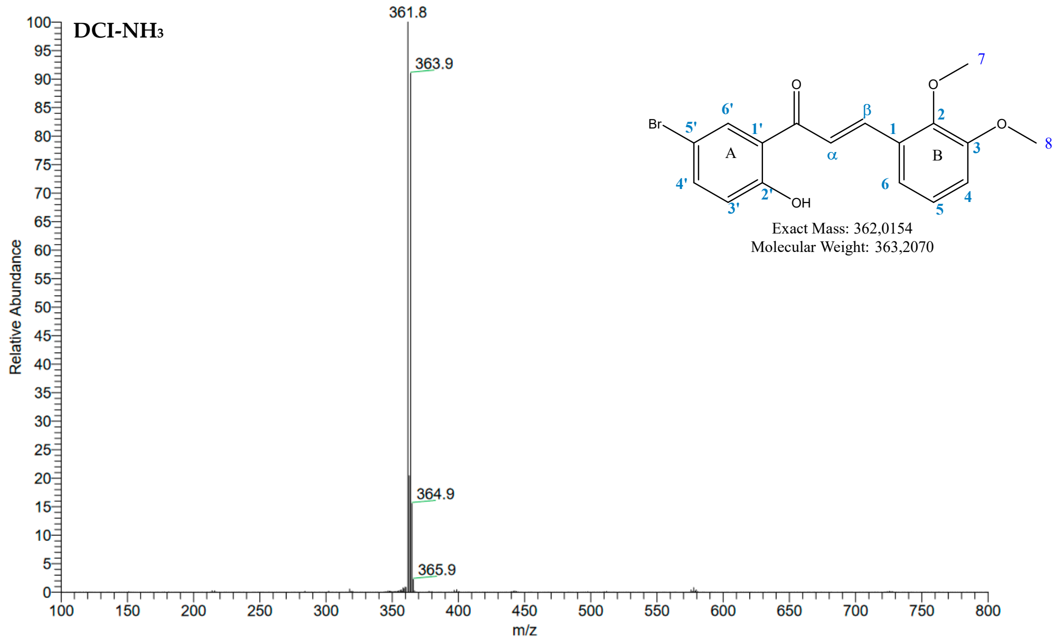

Figure S46 : MS of compound (11)

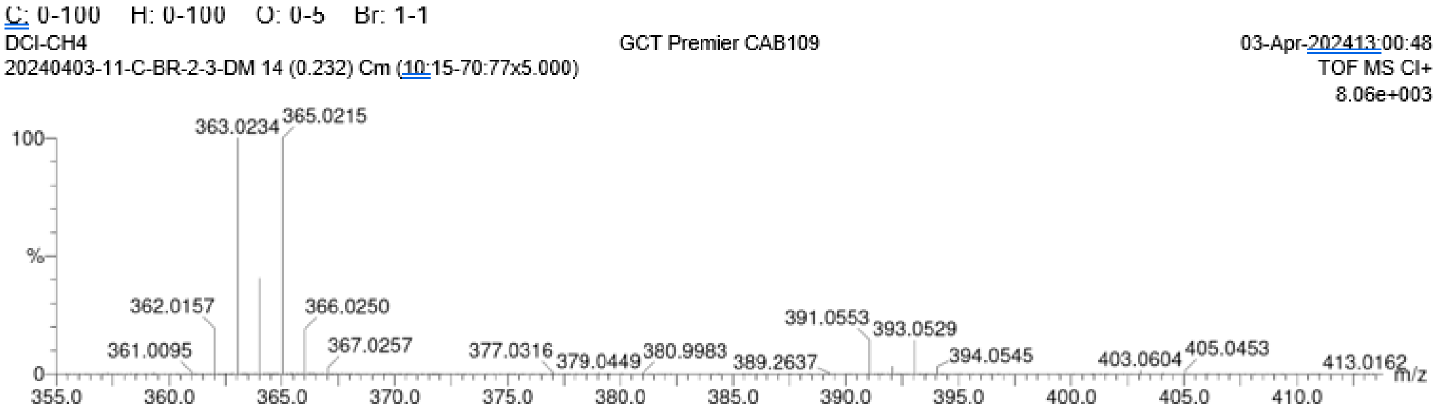

Figure S47 : MS of compound (11)

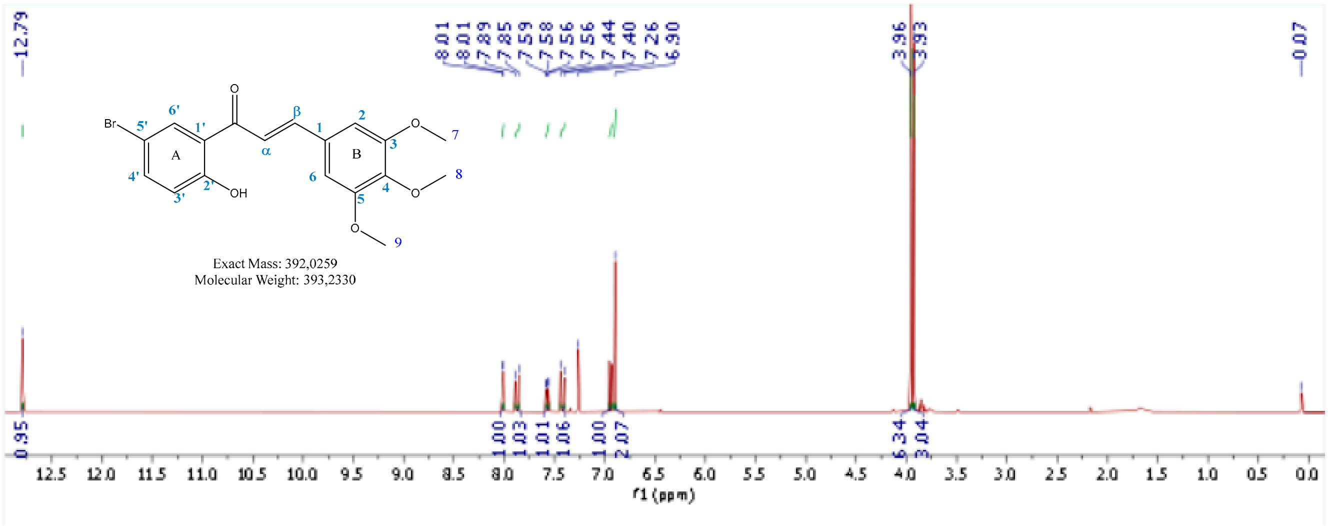

Figure S48 : ^{1}H NMR of compound (12)

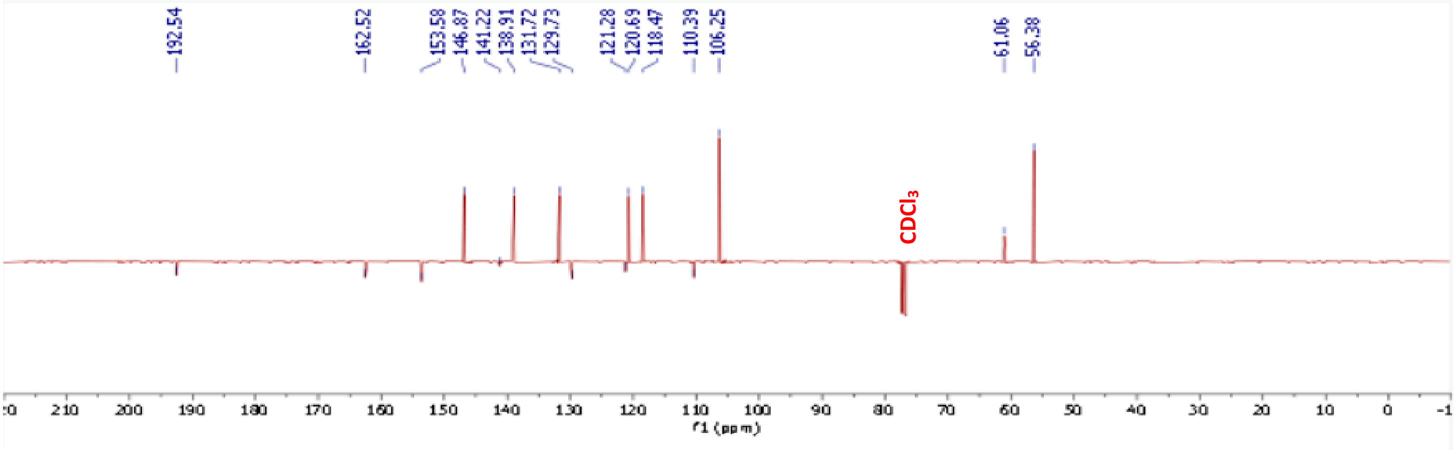

Figure S49 : ^{13}C NMR of compound (12)

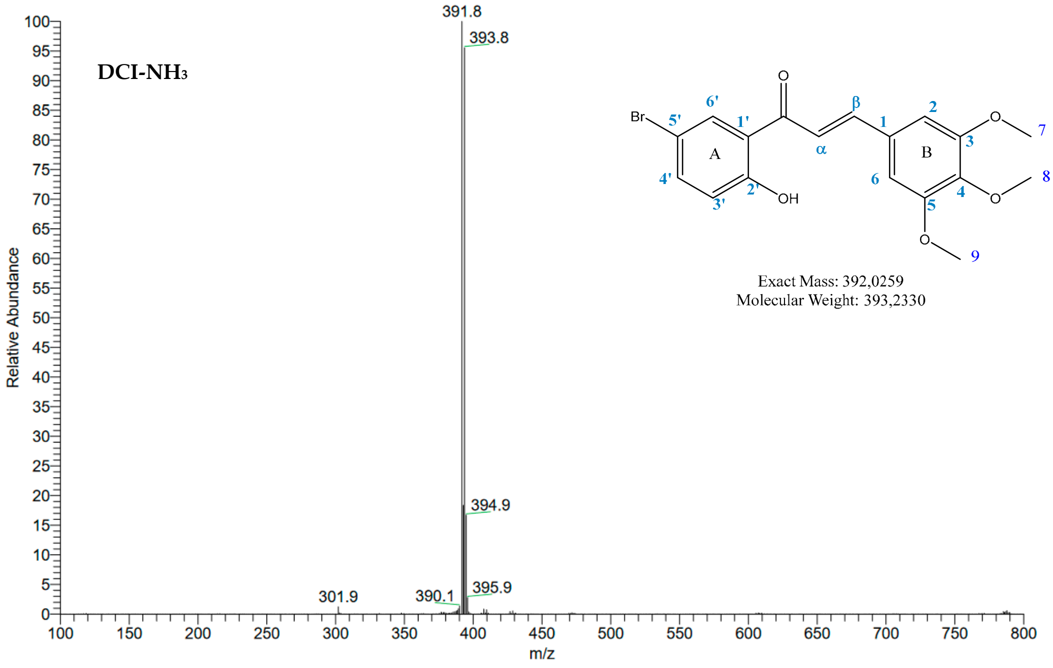

Figure S50 : MS of compound (12)

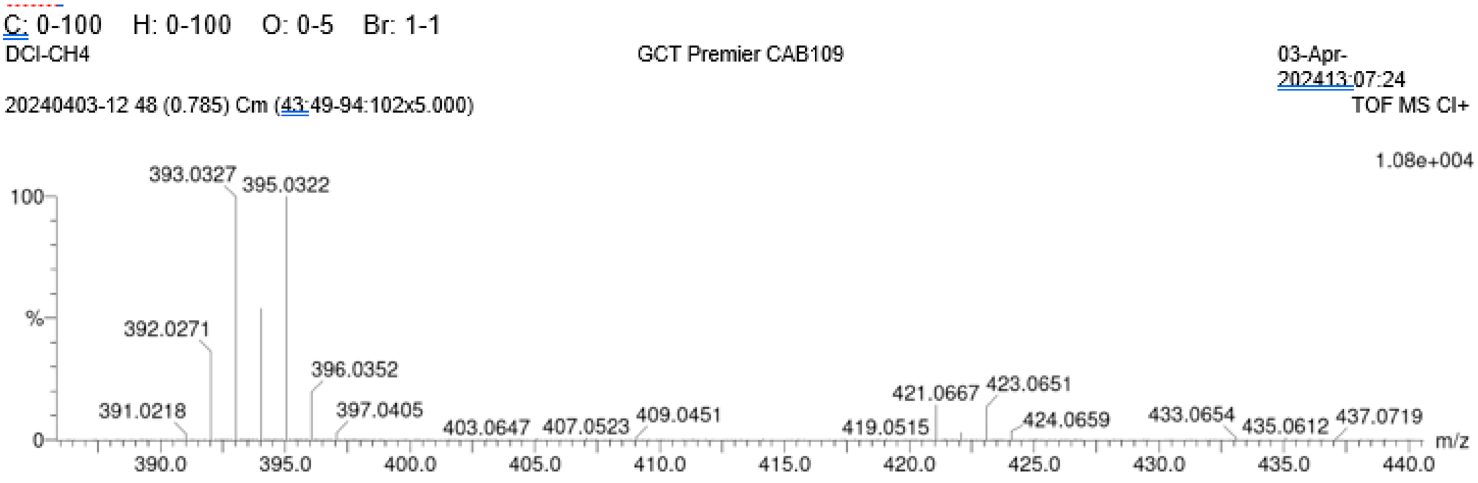

Figure S51 : HRMS of compound (12)

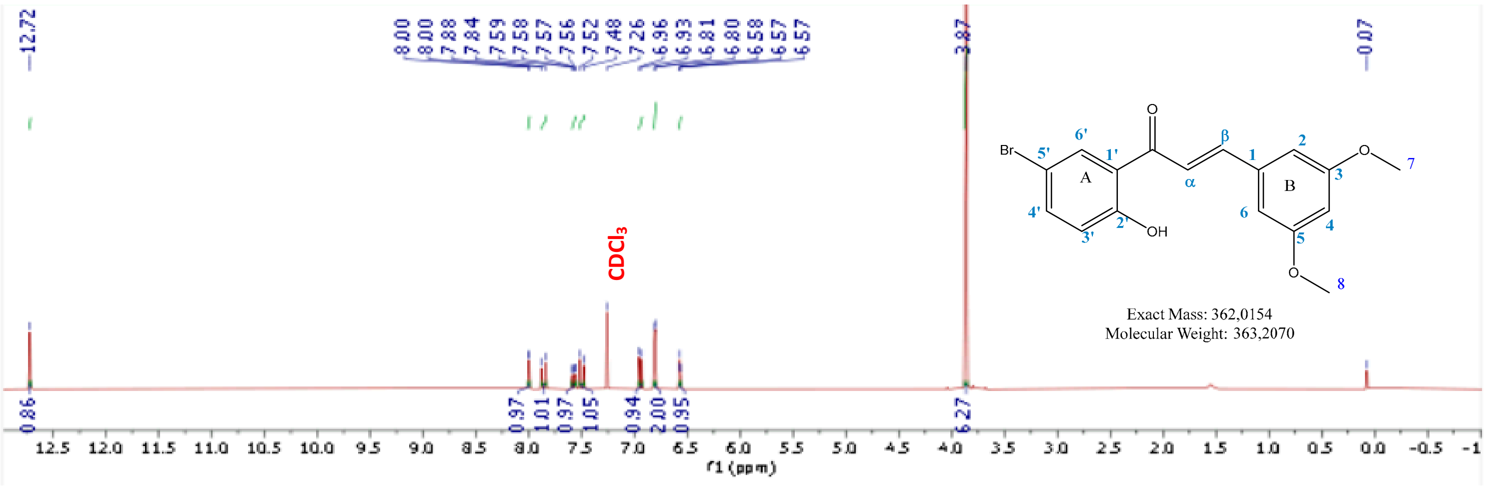

Figure S52 : ^{1}H NMR of compound (13)

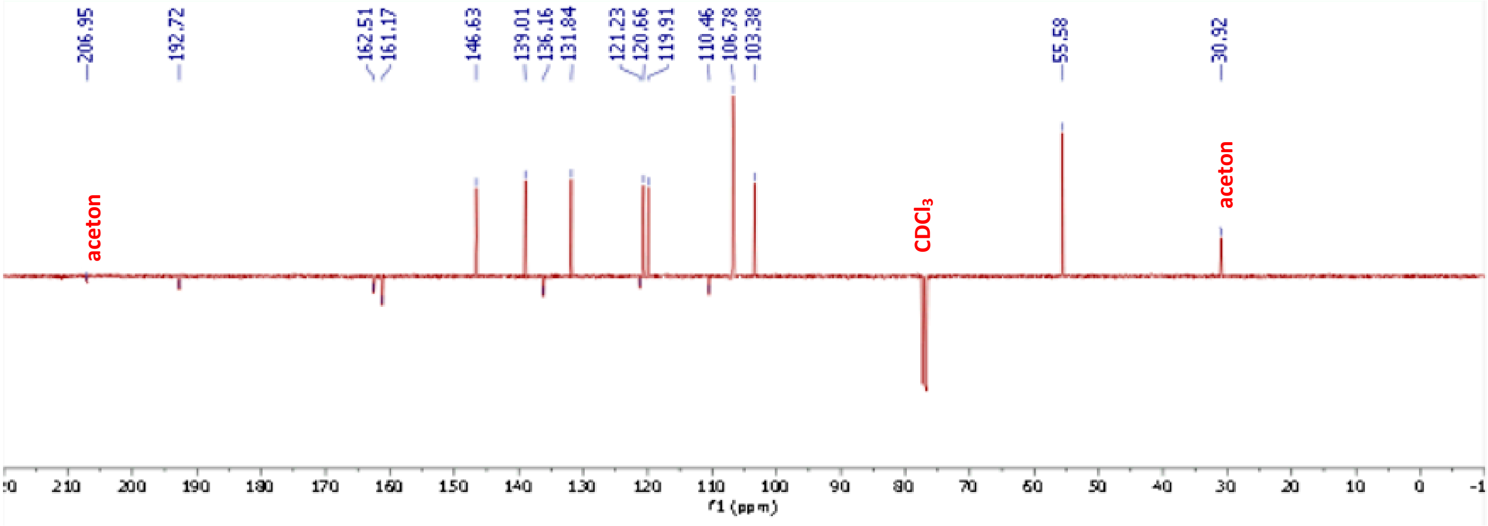

Figure S53 : ^{13}C NMR of compound (13)

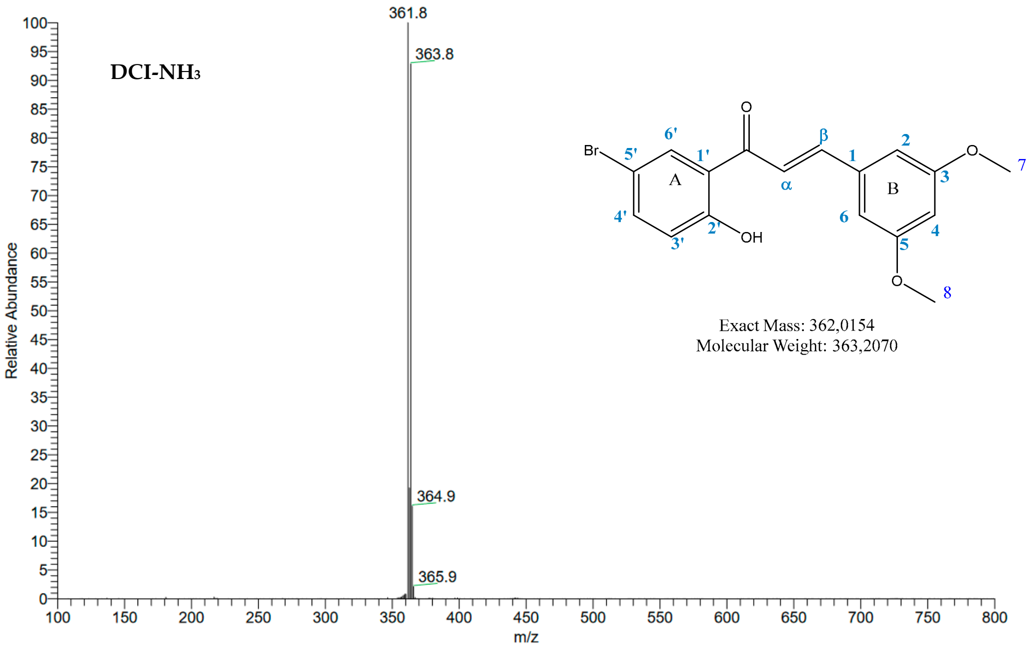

Figure S54 : MS of compound (13)

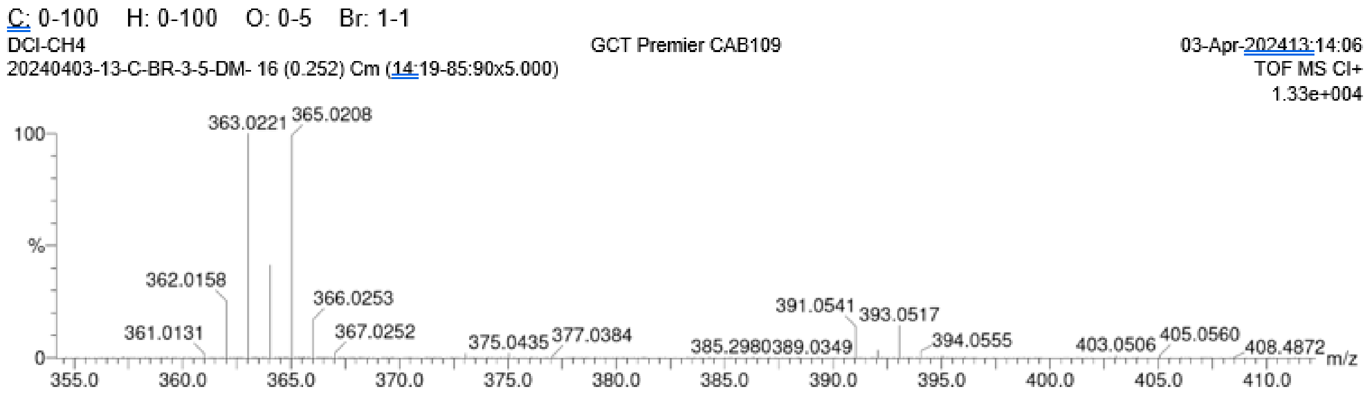

Figure S55 : MS of compound (13)

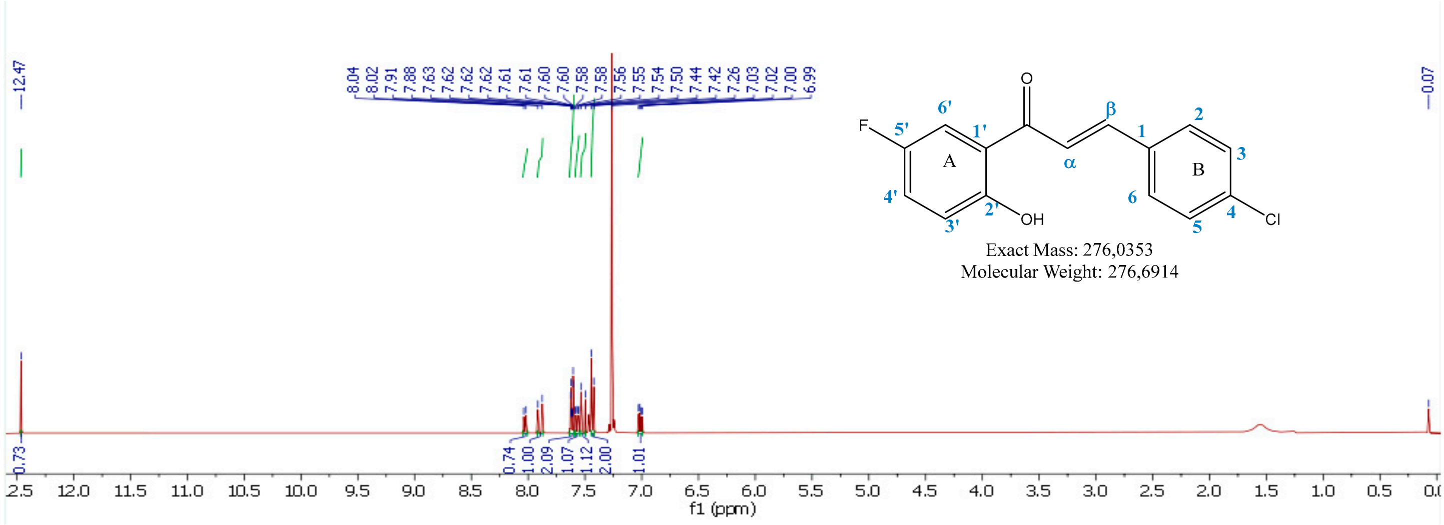

Figure S56 : ^{1}H NMR of compound (14)

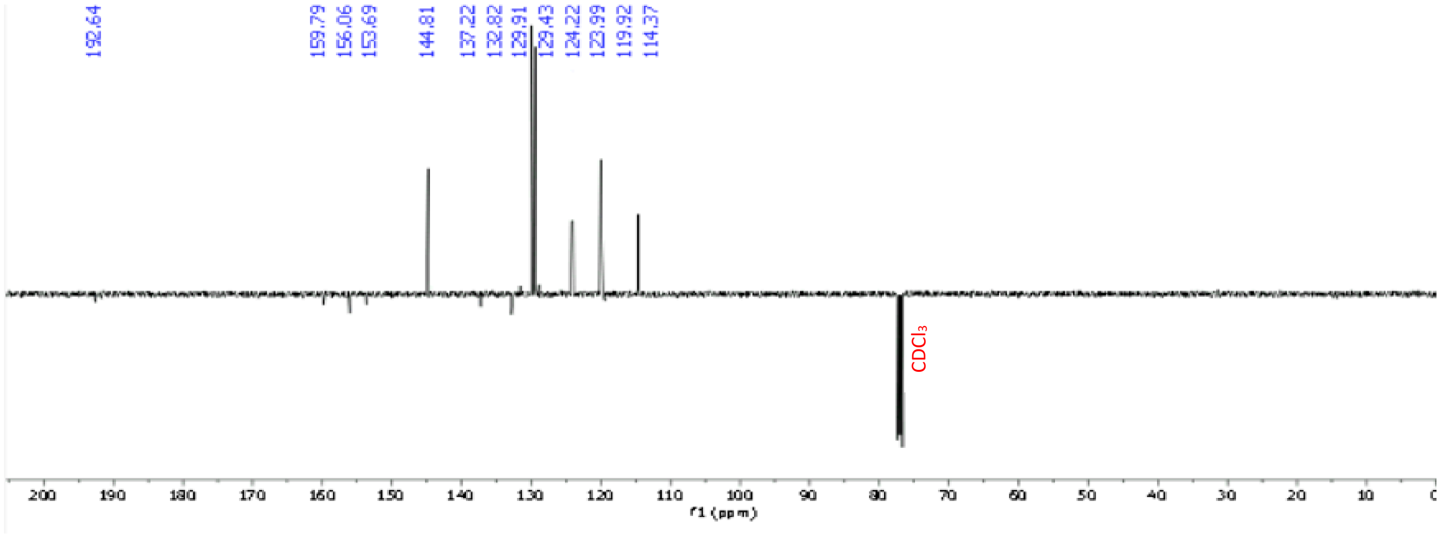

Figure S57 : ^{13}C NMR of compound (14)

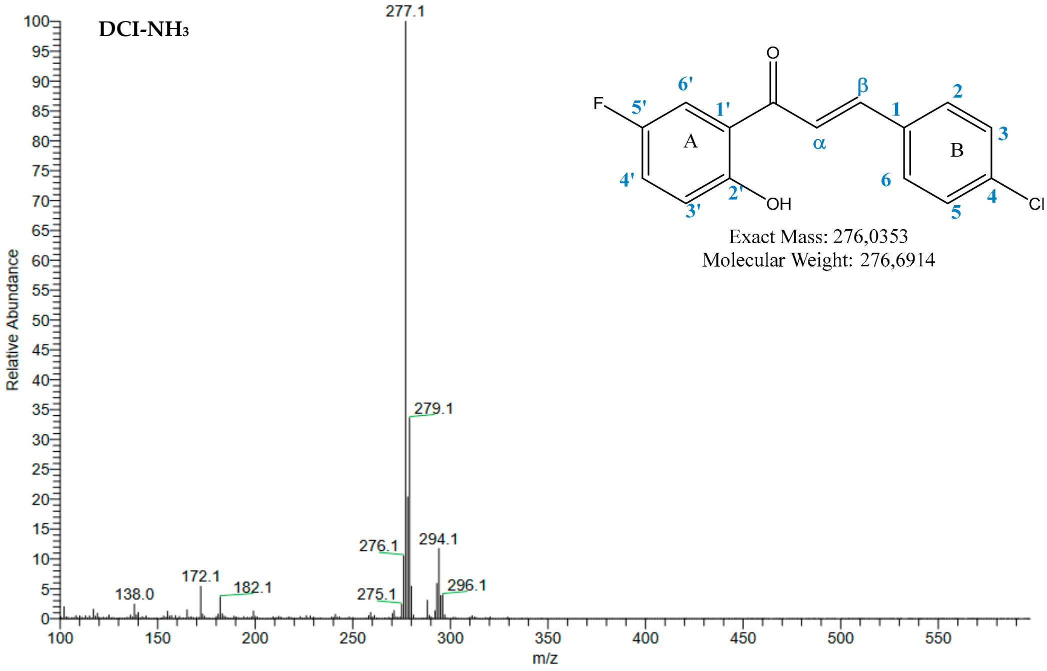

Figure S58 : MS of compound (14)

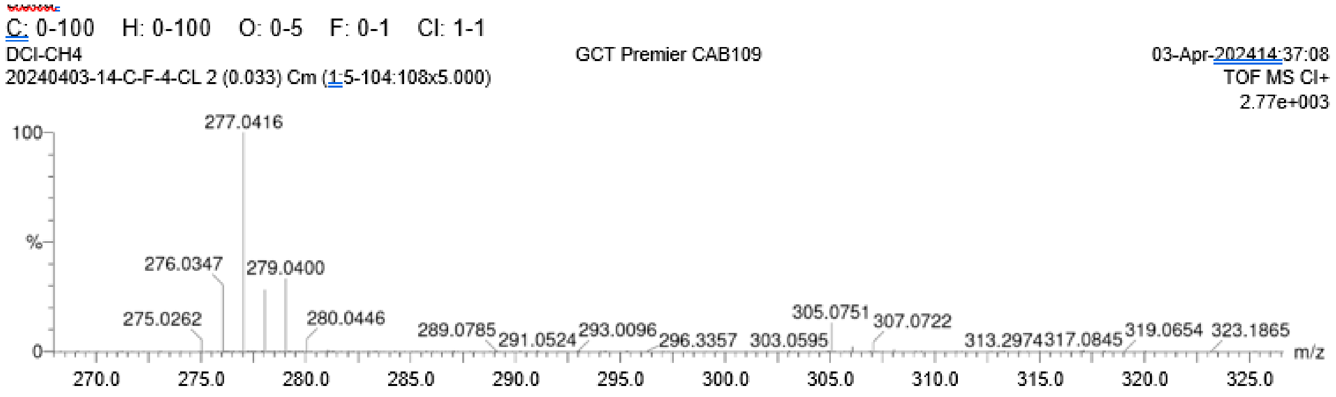

Figure S59 : HRMS of compound (15)

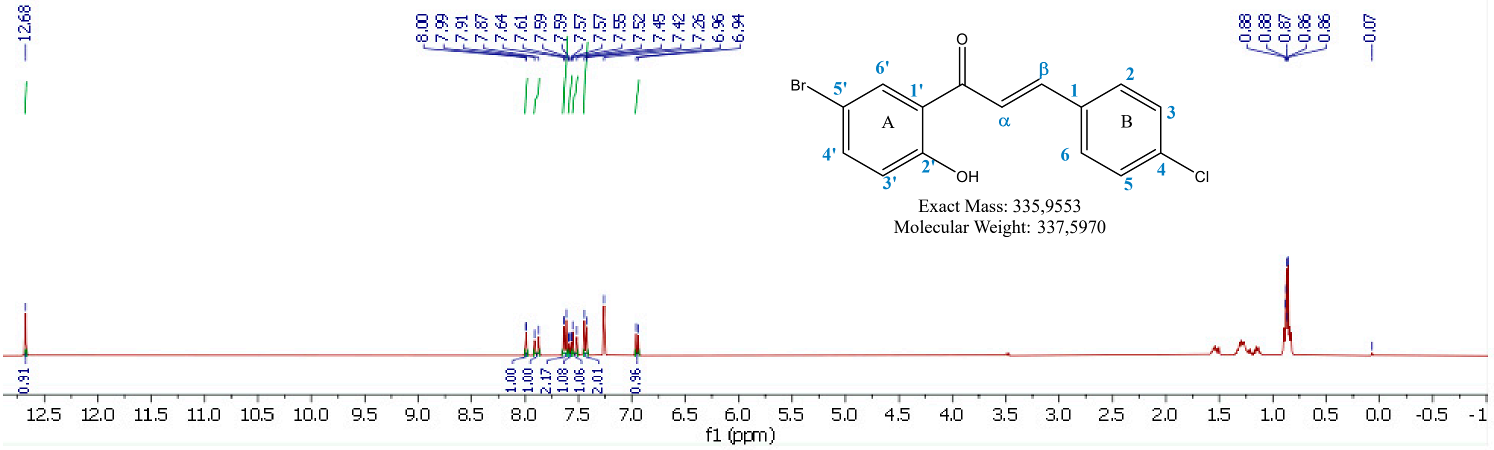

Figure S60 : ^{1}H NMR of compound (15)

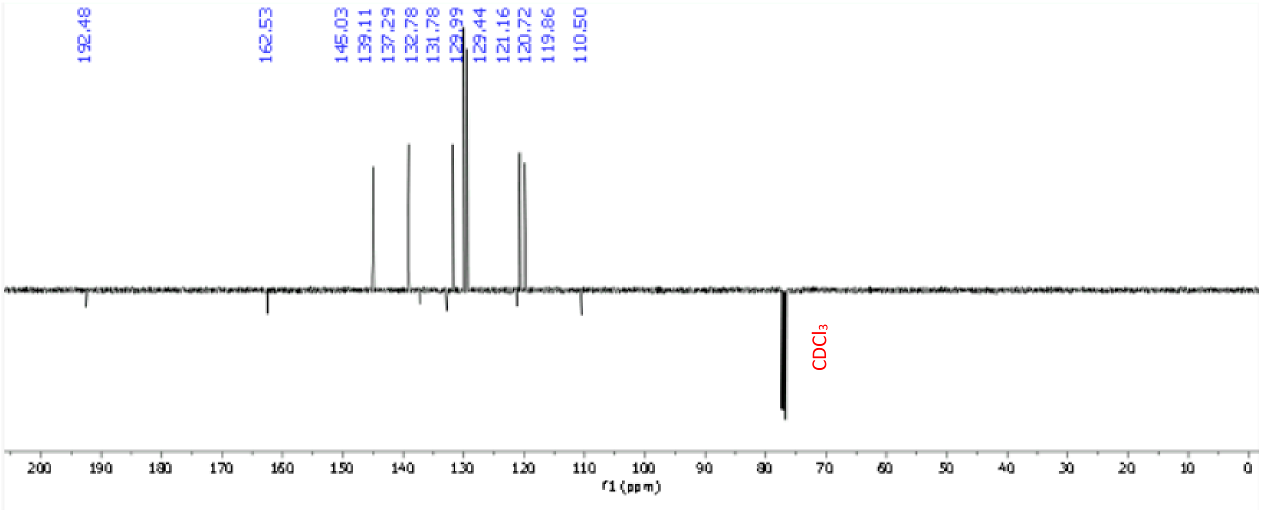

Figure S61 : ^{13}C NMR of compound (15)

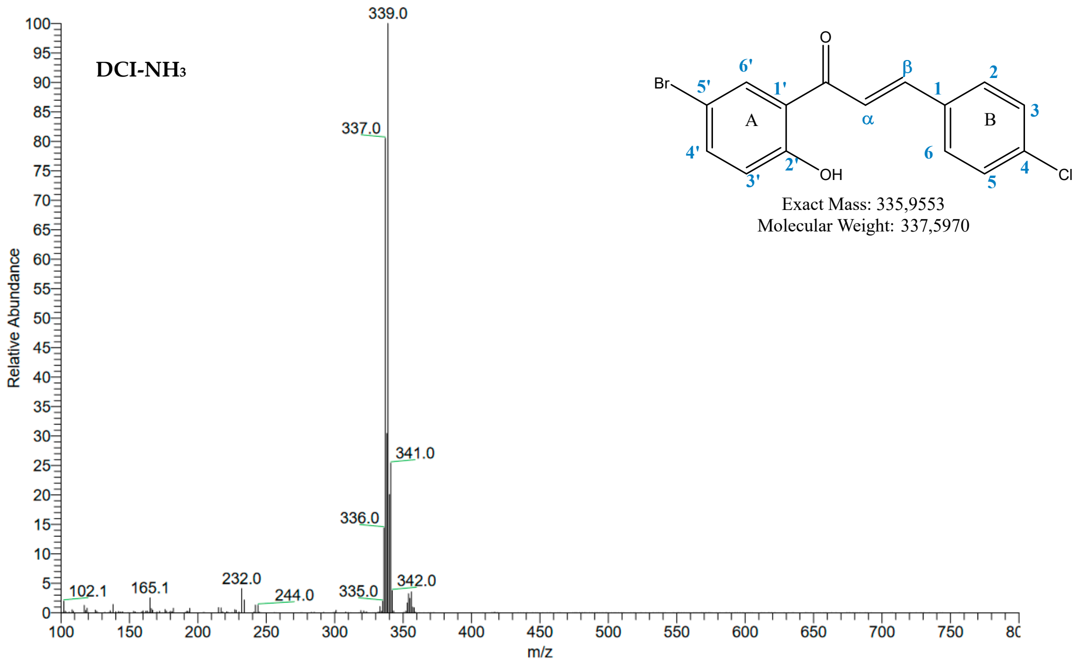

Figure S62 : MS of compound (15)

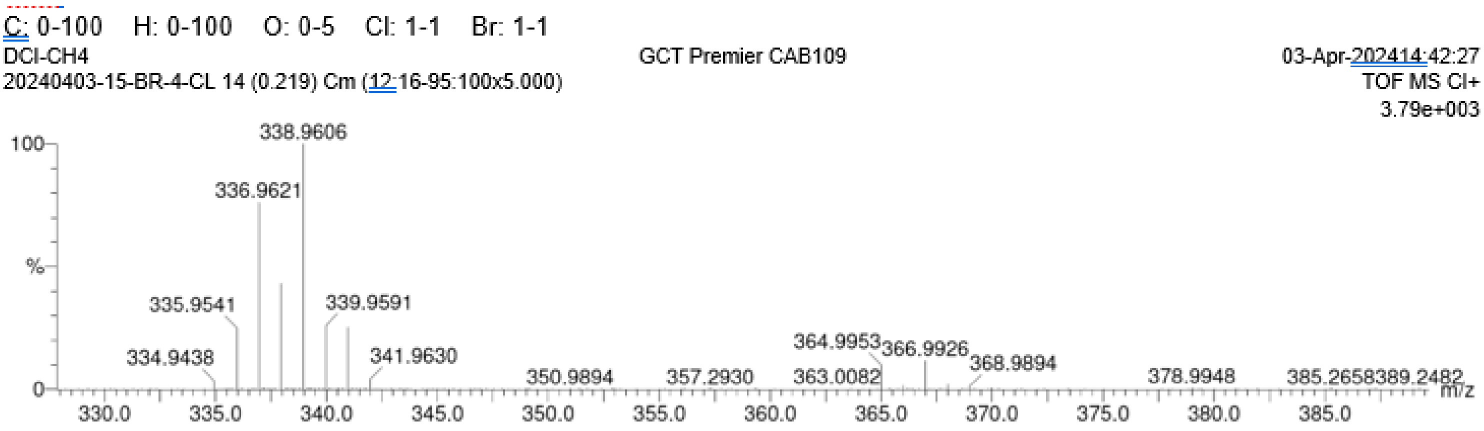

Figure S63 : HRMS of compound (15)

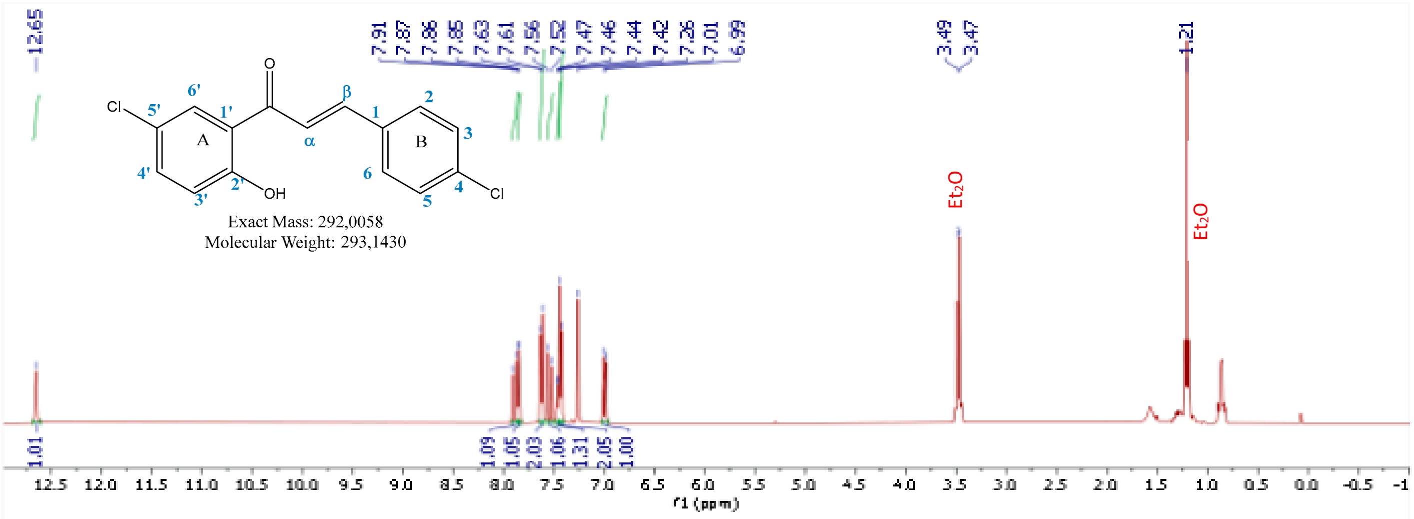

Figure S64 : ^{1}H NMR of compound (16)

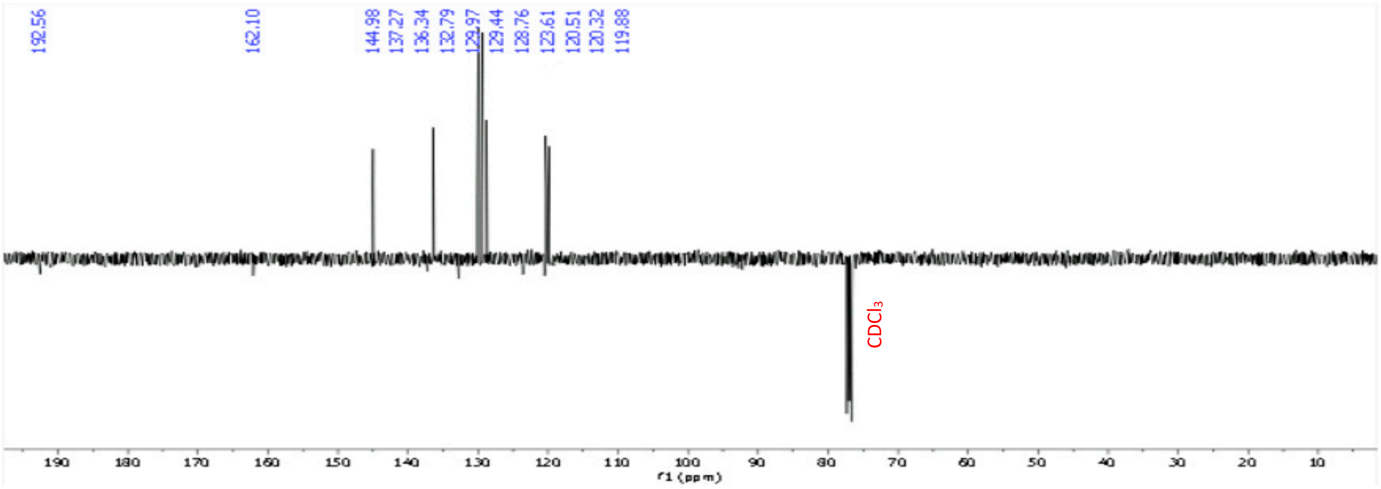

Figure S65 : ^{13}C NMR of compound (16)

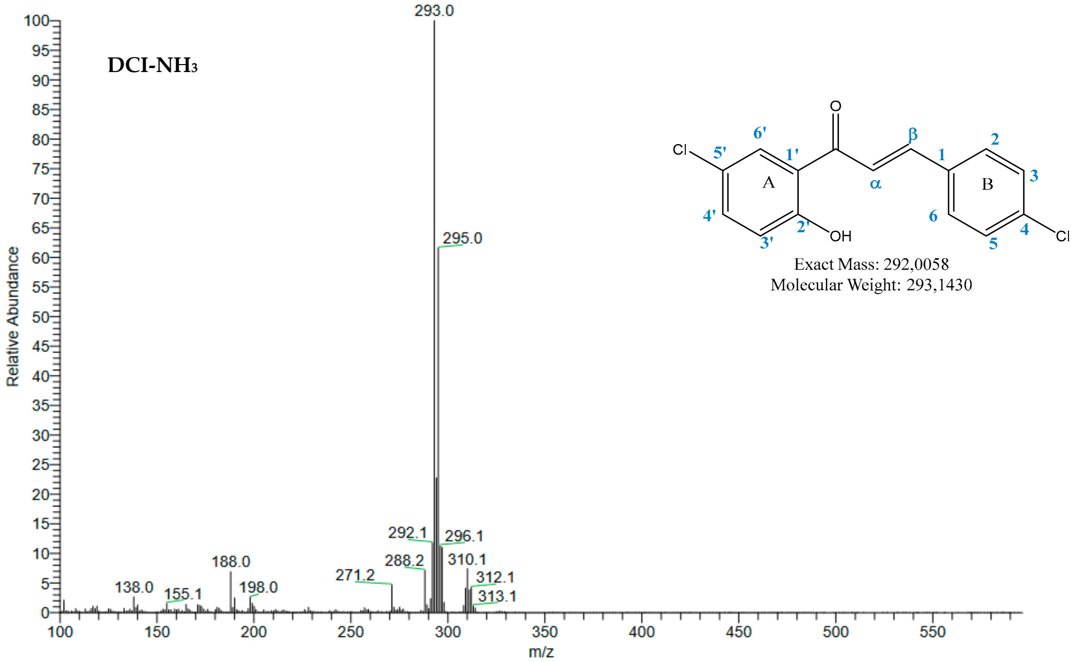

Figure S66 : MS of compound (16)

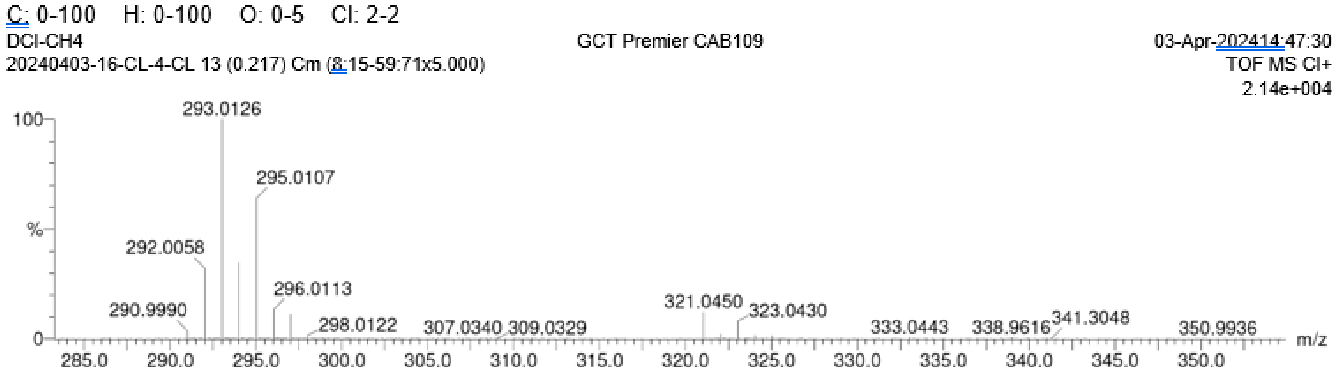

Figure S67 : HRMS of compound (16)

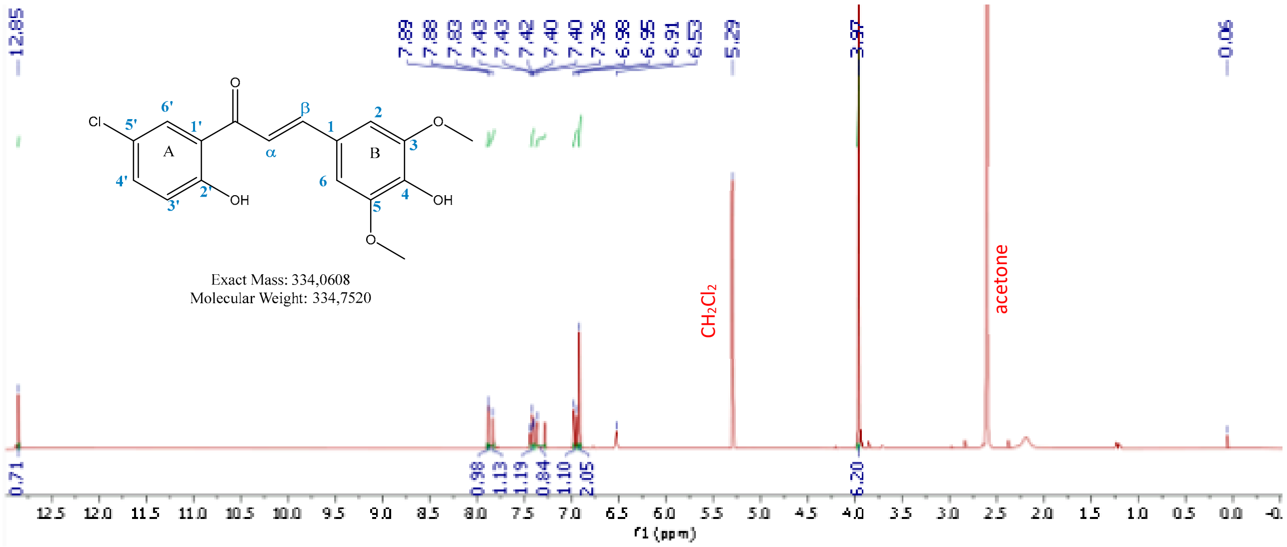

Figure S68 : ^{1}H NMR of compound (17)

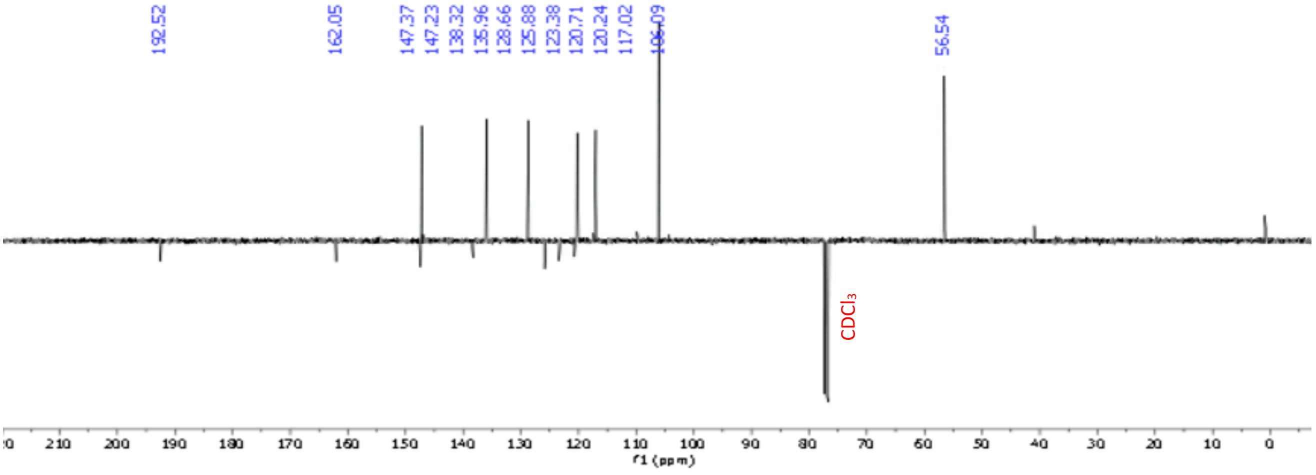

Figure S69 : ^{1}H NMR of compound (17)

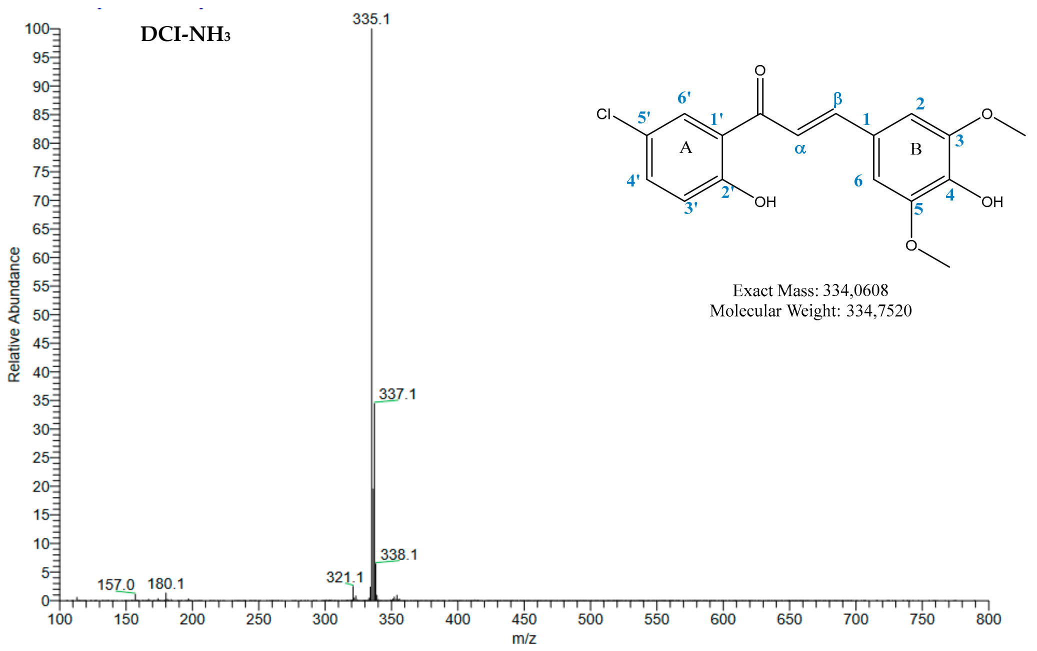

Figure S70 : MS of compound (17)

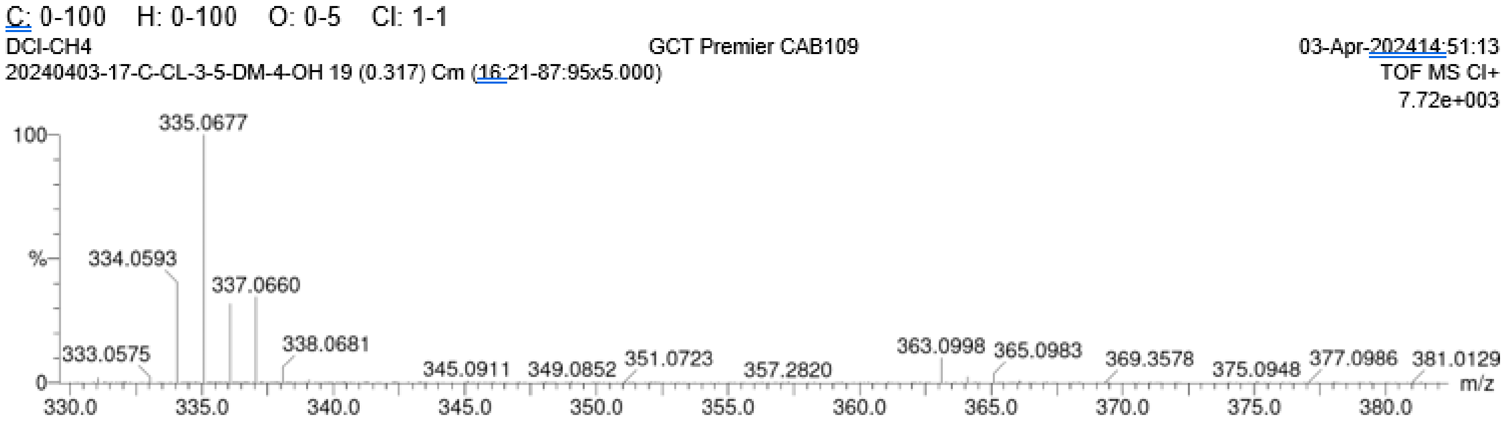

Figure S71 : HRMS of compound (17)

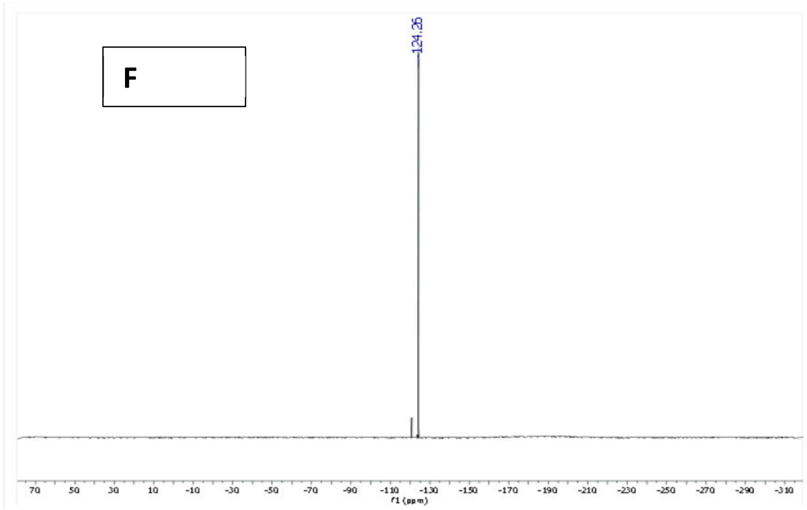

Figure S72 : ^{19}F NMR
